# Comprehensive review of surgical microscopes: technology development and medical applications

**DOI:** 10.1117/1.JBO.26.1.010901

**Published:** 2021-01-04

**Authors:** Ling Ma, Baowei Fei

**Affiliations:** a University of Texas at Dallas, Department of Bioengineering, Richardson, Texas, United States; b University of Texas Southwestern Medical Center, Department of Radiology, Dallas, Texas, United States

**Keywords:** surgical microscope, optics, illumination, mechanical, visualization, image-guided surgery, augmented reality, fluorescence imaging, optical coherence tomography, hyperspectral imaging

## Abstract

**Significance:** Surgical microscopes provide adjustable magnification, bright illumination, and clear visualization of the surgical field and have been increasingly used in operating rooms. State-of-the-art surgical microscopes are integrated with various imaging modalities, such as optical coherence tomography (OCT), fluorescence imaging, and augmented reality (AR) for image-guided surgery.

**Aim:** This comprehensive review is based on the literature of over 500 papers that cover the technology development and applications of surgical microscopy over the past century. The aim of this review is threefold: (i) providing a comprehensive technical overview of surgical microscopes, (ii) providing critical references for microscope selection and system development, and (iii) providing an overview of various medical applications.

**Approach:** More than 500 references were collected and reviewed. A timeline of important milestones during the evolution of surgical microscope is provided in this study. An in-depth technical overview of the optical system, mechanical system, illumination, visualization, and integration with advanced imaging modalities is provided. Various medical applications of surgical microscopes in neurosurgery and spine surgery, ophthalmic surgery, ear-nose-throat (ENT) surgery, endodontics, and plastic and reconstructive surgery are described.

**Results:** Surgical microscopy has been significantly advanced in the technical aspects of high-end optics, bright and shadow-free illumination, stable and flexible mechanical design, and versatile visualization. New imaging modalities, such as hyperspectral imaging, OCT, fluorescence imaging, photoacoustic microscopy, and laser speckle contrast imaging, are being integrated with surgical microscopes. Advanced visualization and AR are being added to surgical microscopes as new features that are changing clinical practices in the operating room.

**Conclusions:** The combination of new imaging technologies and surgical microscopy will enable surgeons to perform challenging procedures and improve surgical outcomes. With advanced visualization and improved ergonomics, the surgical microscope has become a powerful tool in neurosurgery, spinal, ENT, ophthalmic, plastic and reconstructive surgeries.

## 1 Introduction

### 1.1 Background

Various diseases, including cancer, require surgery as a prime treatment method.^1^^–^^7^ One key factor for surgeons to operate accurately is a clear visualization of the anatomical structures.^8^ However, this has never been easy. On the one hand, some anatomical structures are very small, varying from millimeters to microns,^9^ and they might have close proximity to other organs or tissue.^10^ A clear view of these structures requires a resolution well beyond that of human eyes.^11^ On the other hand, the lack of illumination in narrow cavities and deep channels, which are very common in neurosurgery, ear-nose-throat (ENT) surgery, and endodontics, results in a dim visualization with shadows.^10^^,^^12^^,^^13^ Poor visualization may lead to inappropriate operation on anatomical structures or a nearby organ, which will affect the surgical outcome, reduce organ preservation, or even cause life-threatening consequences.^14^ Therefore, sufficient magnification and proper illumination are vital for the success of surgery.

Before the advent of the surgical microscope, surgeons had been using various magnifying systems mounted on spectacles or headbands. These systems can be grouped into three categories, namely single-lens magnifiers, prismatic binocular magnifiers, and telescopic systems.^15^^,^^16^ Single-lens loupes used convex lenses for magnifying with a fixed magnification and a very short working distance. With the desire to have more magnification at a longer working distance, telescopic systems came into use. One of the first closed Galilean telescope systems had a 3× magnification and a working distance of 15 cm. The Keeler Galilean system introduced in 1952 had a 2× magnification at 25 cm. In addition, a set of five different telescopes, which could be separately fixed on a spectacle frame via screws, offered a choice of magnification from 1.75× to 9× and a working distance from 34 to 16.5 cm. The binocular loupe, which uses prism oculars and lenses to achieve stereopsis, was first developed by Westien and modified by von Zehender for the examination of the eye. Later, the Carl Zeiss company presented a binocular loupe with a working distance of 25 cm, which opened the door to modern microsurgery.^16^ However, a head-mounted magnifying system suffers from unstable focusing due to the absence of the supporting structure. In addition, increasing the magnification or adding a light source can also increase the size and weight of the system, making it less comfortable for surgeons to wear.

A surgical microscope, also known as an operating microscope, is an optical microscope specifically designed to be used in a surgical setting, especially requisite for microsurgery.^17^ Although the compound microscope had been invented in 1590^18^^,^^19^ and was used for examination of wounds and scars in the late 17th century,^16^ it had several limitations including the heavy weight, large size, and low image quality due to chromatic and spherical aberrations. Therefore, despite the high magnification, it was not widely adopted in clinical applications until the solutions to the aberrations were found. In the late 19th century, Ernst Abbe proposed numerical aperture and greatly enhanced the resolution of microscopes. Later on, the monocular and binocular microscopes were merged with tripods and attached light sources and were used for various examinations.^15^ However, it was not until 1921 that a monocular microscope truly entered the operating room for an aural surgery. One year later, this idea was modified using a binocular microscope.^20^ Ever since, surgical microscopes have been evolving with a wider range of magnification, longer working distance, better illumination, and more stable supporting structures. The benefits were soon acknowledged by otolaryngology surgeons and gradually recognized by surgeons in other fields.

Surgical microscopes of the time have been refined to a precision instrument with several appealing features.^19^ They have high-precision optics and high-power coaxial illumination, which provide surgeons with adjustable magnifications, proper working distance, and an unobstructed view of the entire operating field.^10^ The well-designed mechanical system offers stability and maneuverability, while the heads-up display improves ergonomics.^19^^,^^21^^–^^25^ Stereopsis provides the third dimension of the field of view (FOV) thereby increasing the safety for surgery.^26^ Multiple optical ports are available on the microscope for assistant observers or adaptation of video cameras. Moreover, contemporary surgical microscopes are enriched with various intraoperative imaging modules such as fluorescence imaging^27^^–^^35^ and optical coherence tomography (OCT),^36^^–^^42^ and they are open for adaptation of other imaging modalities including hyperspectral imaging (HSI), photoacoustic microscopy (PAM),^43^^–^^48^ and laser speckle contrast imaging (LSCI). Augmented reality (AR)^49^^–^^54^ has been actively evaluated on the surgical microscope and has offered huge convenience for surgery as an intraoperative diagnostic tool. Furthermore, high-definition (HD) display,^22^^,^^55^^,^^56^ image injection techniques,^50^^,^^57^^,^^58^ and three-dimensional (3D) display facilitate better visualization of both the surgical field and the multimodality images.

With numerous advantages such as clear and bright visualization, easy documentation and adaptation, stability, maneuverability, and improved ergonomics, surgical microscopes have been applied in various types of surgeries, including neuro and spine surgery,^8^^,^^10^^,^^50^^,^^59^^–^^61^ ENT surgery,^5^^,^^20^^,^^51^^,^^62^^–^^64^ dentistry,^11^^,^^13^^,^^65^^–^^77^ ophthalmology,^15^^,^^36^^–^^38^^,^^40^^–^^42^^,^^78^^–^^80^ and plastic and reconstructive surgery.^14^^,^^81^^–^^86^ For example, they have been used for brain tumor resection,^27^ aneurysm surgery,^87^ nasal surgery,^88^ head and neck cancer resection,^89^ corneal keratoplasty,^37^ vitreoretinal macular hole repair,^90^ root therapy,^91^ root coverage procedure,^92^ craniosynostosis surgery,^93^ and hepatic artery reconstruction.^94^ For different applications, microscopes are modified into slightly different optical configurations and equipped with specific imaging modalities. The end-users of surgical microscopes include hospitals, dental clinics, other outpatient settings, and some research institutes.^95^

### 1.2 Perspective on Surgical Microscopes

Surgical microscopes have gone through a long evolution and development. Because of numerous attractive features and new imaging modalities, surgical microscopes will not stop here but will continue to thrive. The limitations of large volume, high cost, and potential tissue damage by high-power illumination will be further addressed with robotic positioning, increasing utilization, and better light management. Three main future directions of the surgical microscope include being integrated with more advanced technologies, launching new ways for visualization, and being increasingly used in more clinical applications.

First, the integration of HSI, LSCI, PAM, and polarization imaging with surgical microscopes and the related imaging processing methods will become more mature and well-developed, to provide surgical guidance in addition to that of fluorescence imaging and OCT. HSI and LSCI are particularly promising since they are noncontact and label-free, not requiring any injection of any contrast or dye. They can be used anytime during the surgery without administration time and provide abundant quantitative diagnostic information in real-time. Meanwhile, both HSI and LSCI have a very simple system for adaptation and an easy interpretation of images. Therefore, it takes minimal effort for physicians to adopt these methods. The endoscopic tool in the newest robotic visualization system also opens opportunities for some imaging modalities that are not as easy to be adapted with conventional surgical microscopes, such as confocal microscopy and Raman spectroscopy. Second, the visualization of surgical microscopes will be expanded. It will not be limited to a clear view shared only by the team in the operating room. New ways of visualization will give surgeons the freedom to visualize the procedures anywhere through monitors, headsets, smartphones, and large screens in the conference room. With advanced communication technologies and well-developed AR-assisted platforms, a larger group will be able to participate in the procedures remotely. Finally, it is anticipated that surgical microscopes will be increasingly used in more applications such as orthopedic spine surgery and cataract surgery. In particular, surgical microscopes have brought a revolution in dentistry, but they were mainly used in endodontics. Therefore, it is promising for surgical microscopes to be more adopted in other dental applications such as periodontal surgery. In addition, the new endoscopic tool and the picture-in-picture visualization mode give users access to more deep structures, which greatly increases the competitiveness of surgical microscopes in various ENT surgeries.

### 1.3 Organization of the Following Sections

We aim to introduce and explain the surgical microscope and to give an overview of the literature on surgical microscope history, technologies, and applications. (1) We start with the history of the surgical microscope and give a timeline of milestones during the development of the surgical microscope. (2) We introduce the surgical microscope from technical aspects, including its optical system, illumination system, mechanical system, visualization system, and combination with other technologies, such as AR, fluorescence imaging, and OCT. (3) The section on applications refers to the available literature on how surgical microscopes are utilized in surgery. These applications mainly cover neurosurgeries and spine surgeries, ENT surgeries, ophthalmic surgeries, dental operations, as well as plastic and reconstructive surgeries. (4) We conclude with a discussion about the limitations and future directions of surgical microscopes.

## 2 History

### 2.1 Microscope Entering Operating Rooms

**Fig. 1:**
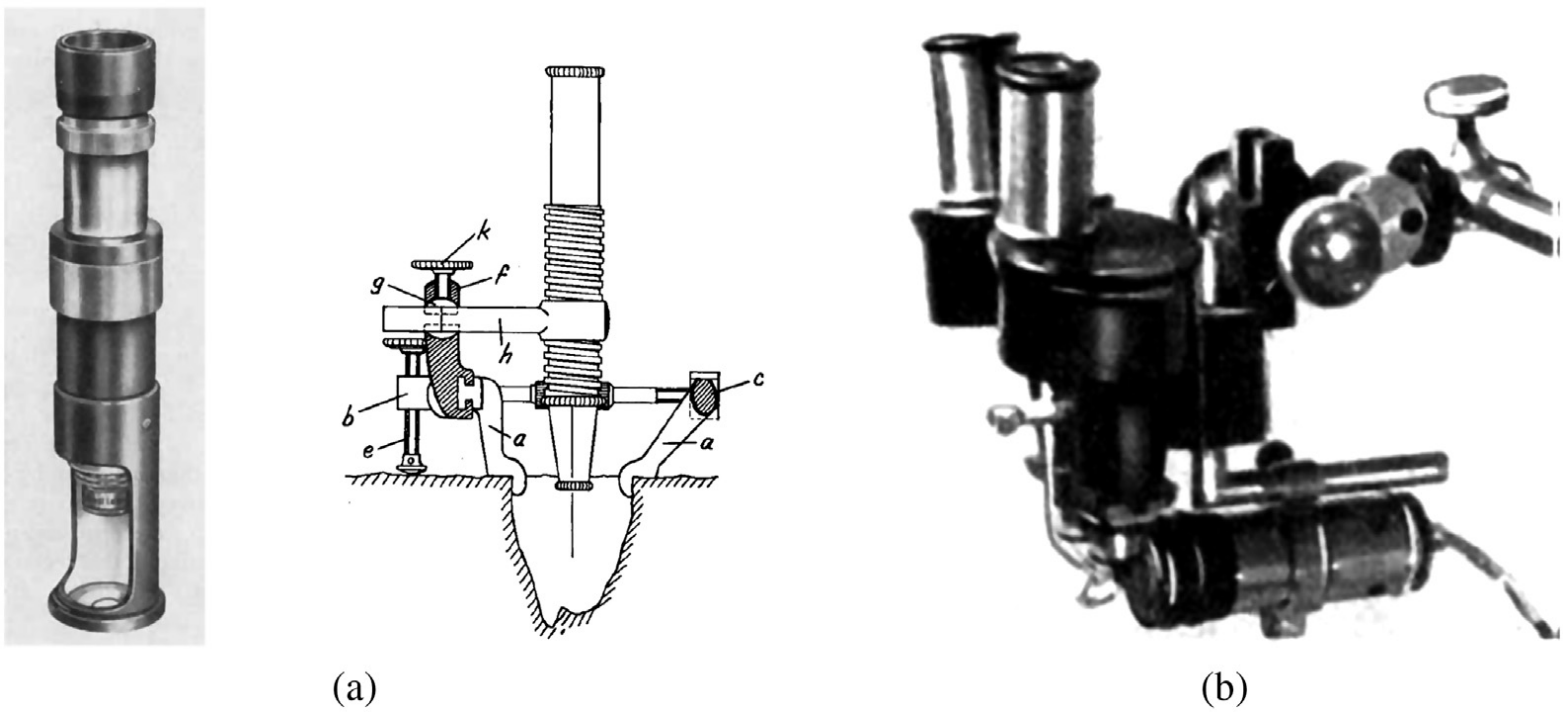
The earliest surgical microscopes in the operating room: (a) Brinell–Leitz monocular microscope used by Carl Olof Nylen and the modified Brinell microscope and (b) Zeiss binocular microscope adapted by Holmgren.^20^

In 1921, Carl Olof Nylén (1892–1978), a young otolaryngology surgeon at the University of Stockholm, used a monocular Brinell–Leitz microscope during surgery on a patient with chronic otitis.^20^^,^^96^ It was reported as the first surgical microscope in the operating room. One year later, Gunnar Holmgren modified this idea with a binocular microscope attached with a light source.^19^^,^^20^ The binocular microscope provided depth perception that was absent in a monocular microscope, and the attached light source overcame the dimness of the image with increased magnification.^19^^,^^20^^,^^97^ Figure 1 shows Nylén’s first surgical monocular microscope and the binocular microscope of Holmgren. After being used in ENT surgeries, the binocular surgical microscope was then introduced into ophthalmology by the ophthalmologic surgeon Richard A. Perritt in 1946.^98^ Although nowadays neurosurgeries form the leading market of surgical microscopes, this instrument was not introduced in the neurosurgical operating room until 1957, when Theodor Kurze at the University of Southern California, Los Angeles, removed a neurilemoma from cranial nerve VII in a 5-year-old patient.^19^ Kurze was inspired by House, who removed an acoustic neurinoma and published his experience in 1963.^10^^,^^16^ Two years later, Pool and Colton used the microscope for intracranial aneurysmal surgery.^10^ In 1955, Fritz Zöllner reported using a binocular surgical microscope in 120 attic-antrotomies with plastic operations.^99^ In 1959, Wolfgang Walz from Heidenheim (Brenz), Germany, reported his experience with microsurgical reconstructive surgery of an occluded fallopian tube.^16^^,^^17^ The introduction of the surgical microscope in endodontics was the latest. In 1978, the first dental surgical microscope (DSM) was produced by Apotheker and Jako,^100^ and in 1981 the design was incorporated into the first commercially available surgical microscope for dentistry.^101^

### 2.2 System Refinement

The advent of the surgical microscope and its early applications in aural surgeries offered better surgical outcomes, and the improved vision helped relieve other diseases in the temporal bone.^20^ Nevertheless, surgical microscopes in the early times had several weaknesses. The low stability of the tripod decreased the quality of images at high magnification,^19^ and the fixed magnification, small FOV, as well as insufficient illumination limited the effective vision of the surgical field.^20^ Furthermore, the old microscopes only allowed one surgeon to view the surgical field, which was inconvenient for assistance. In the following years, refinements have been made for surgical microscopes and made it a real precision and fundamental instrument in the operating room.

The first modern surgical microscope tripod was built in 1938 when P. Tullio and P. Calicetti at the University of Parma constructed a heavy tripod with counterweights for the optical unit. The tripod was able to hang the optical unit freely above the surgical table and stabilize the image, thus it offered comfortable distance and mobility during the procedures.^16^ In 1951, the V. Mueller & Co. started to market a microscope with a suspension system consisting of a weighted table stand, then a ceiling-mount microscope suspension system was designed by Joaquin Barraquer in 1956.^19^ In addition, Horst L. Wullstein from Gottingen, Germany, built a microscope mounted on a stand equipped with a rotating arm. This idea greatly improved the mechanical flexibility of the microscope and was employed in the “Zeiss OPMI 1” (Zeiss Operating Microscope 1),^19^^,^^102^ which was a milestone in the history of the surgical microscope.

In addition to the heavy tripod, Tullio and Calicetti’s invention in 1938 also included mounting prisms between the oculars and objectives, so that the identical view of the surgical field was successfully shared to an assistant surgeon.^16^ And in 1964, Littman adapted a beam splitter to a Zeiss microscope to allow a second surgeon to assist and named the microscope “Diploscope.”^19^

The working distance of a surgical microscope gives a surgeon space to handle surgical instruments. The working distance of the first monocular microscope was 60 mm, and the first binocular microscope had a working distance of 75 mm. They were acceptable but shorter than the ideal length of 200 mm proven by most otology operators at that time.^20^ In 1946, the available working distance was improved to 228 mm, and 250 mm in 1948. Zeiss OPMI 1 came out in 1953 and had a working distance of 100 to 405 mm.^19^ Since then, working distance has been improving to meet the need of different types of surgeries varying from 200 to 500 mm.^10^^,^^103^^–^^105^

**Fig. 2:**
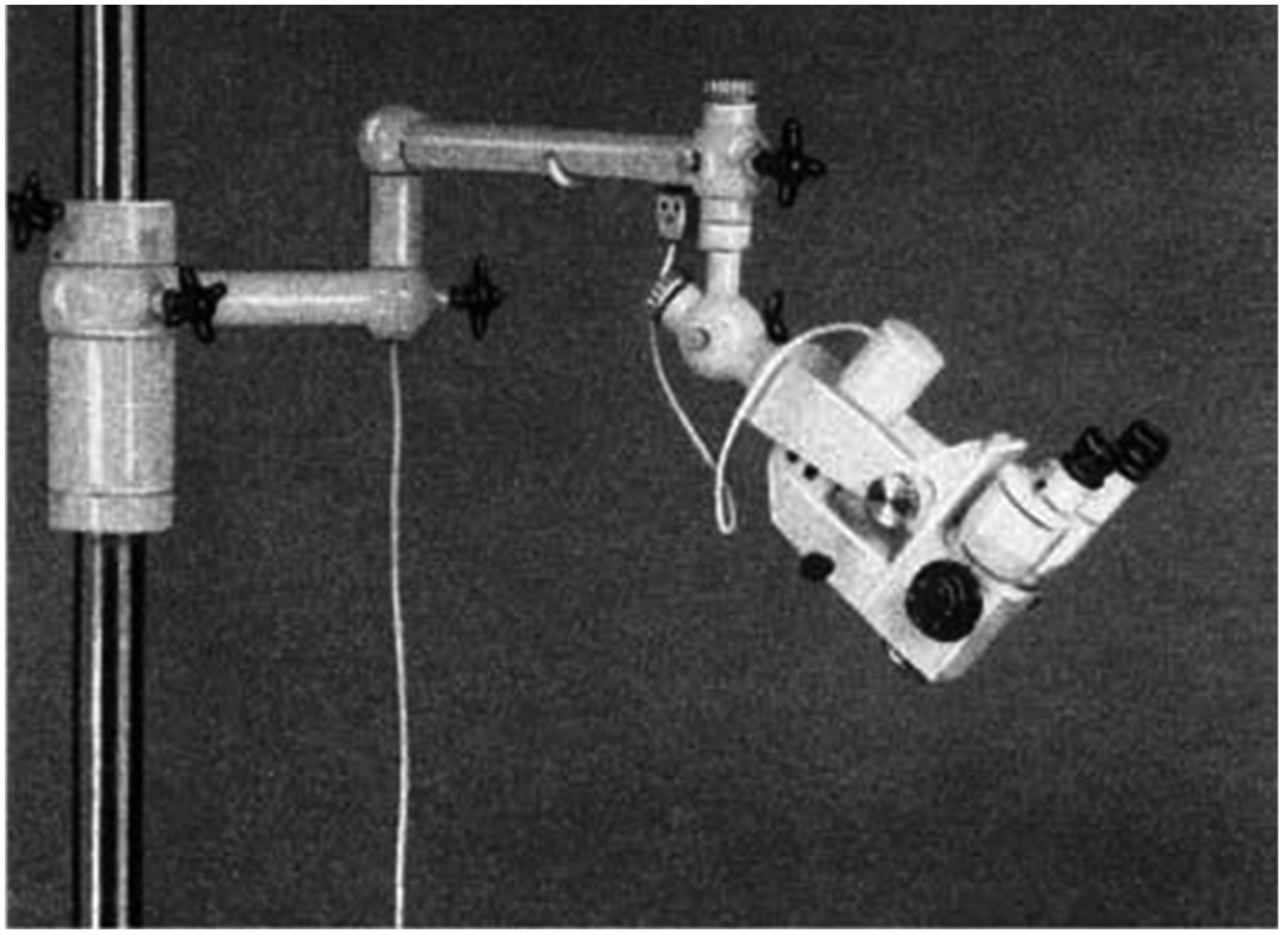
Zeiss-Opton microscope by Hans Littman had various magnification options and one working distance.^20^

A changeable magnification, which is a primary feature of modern surgical microscopes, was first achieved in 1948 by changing the eyepieces on a modified Bausch & Lomb slit-lamp microscope.^19^ This microscope had a working distance of 127 mm and variable magnifications of 3×, 5×, 7×, or 10.5×. In 1953, Hans Littman (1907–1991) invented the microscope that was capable of changing magnifications without changing the focal length.^19^^,^^20^ The so-called “Zeiss-Opton” microscope (Fig. 2) with a working distance of 200 mm and magnification options of 4×, 6×, 10×, 16×, 25×, 40×, and 63× was a start of a new era.

### 2.3 Evolution of Handling and Practical Operation

The invention of Zeiss OPMI 1 in 1953 was a momentum in the development history of surgical microscope.^19^ Its coaxial illumination had superior performance than other contemporaneous microscopes.^18^ Afterward, lots of refinements have been made to improve the operation of the surgical microscope including stability, flexibility, documentation, and share of view.

**Fig. 3:**
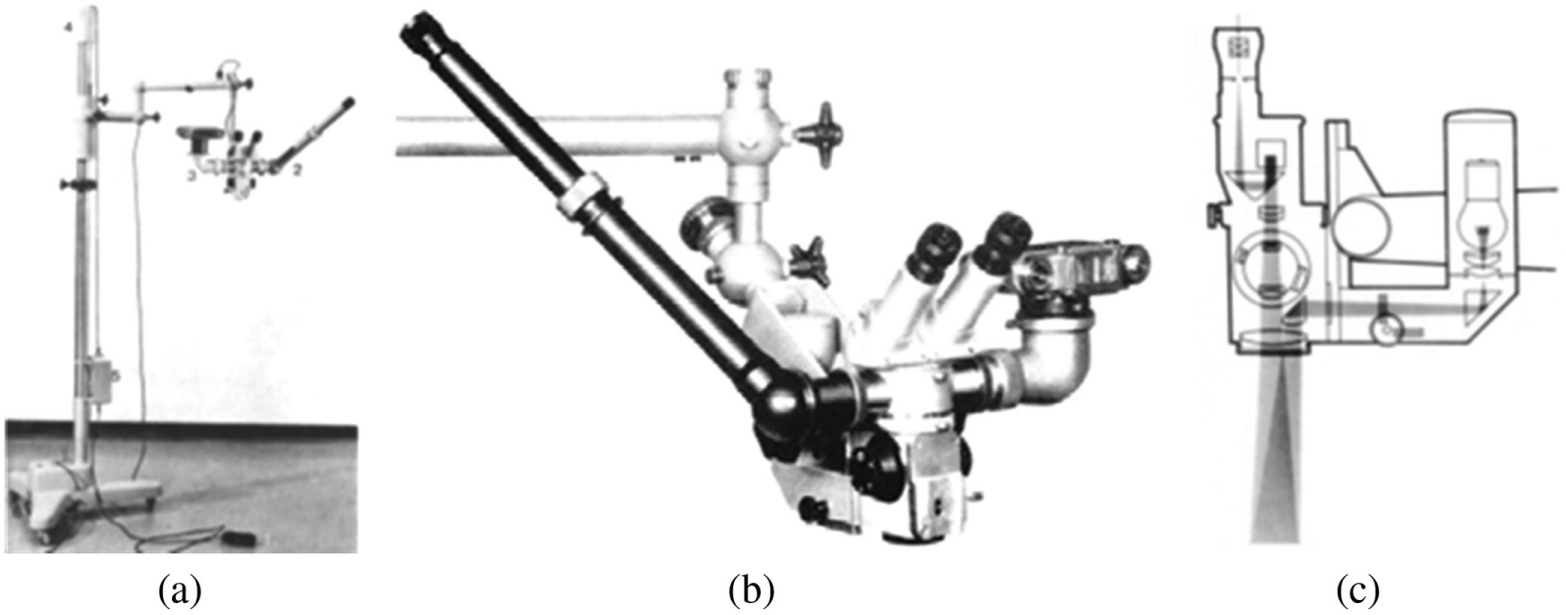
Surgical microscope Zeiss OPMI 1: (a) Zeiss OPMI 1 on its stand with motorized head, (b) Zeiss OPMI 1 with a camera, and (c) Zeiss OPMI 1 optical diagram.^10^^,^^26^

The OPMI 1 microscope had a detachable binocular tube that could be replaced by an angled binocular tube. For the stand, which contained a counterbalancing weight and rotating arm, Littman adopted Wullstein’s idea but achieved better stability and operability. Later, an electric motor was added to the stand to provide up-and-down motion with a foot pedal. The microscope was capable of being attached with cameras or a second eyepiece with a beam divider between the optical head and the binocular tube. In the same year, three ophthalmological surgeons used this model for surgery.^102^ Figure 3 shows the photo and optical diagram of Zeiss OPMI 1.

**Fig. 4:**
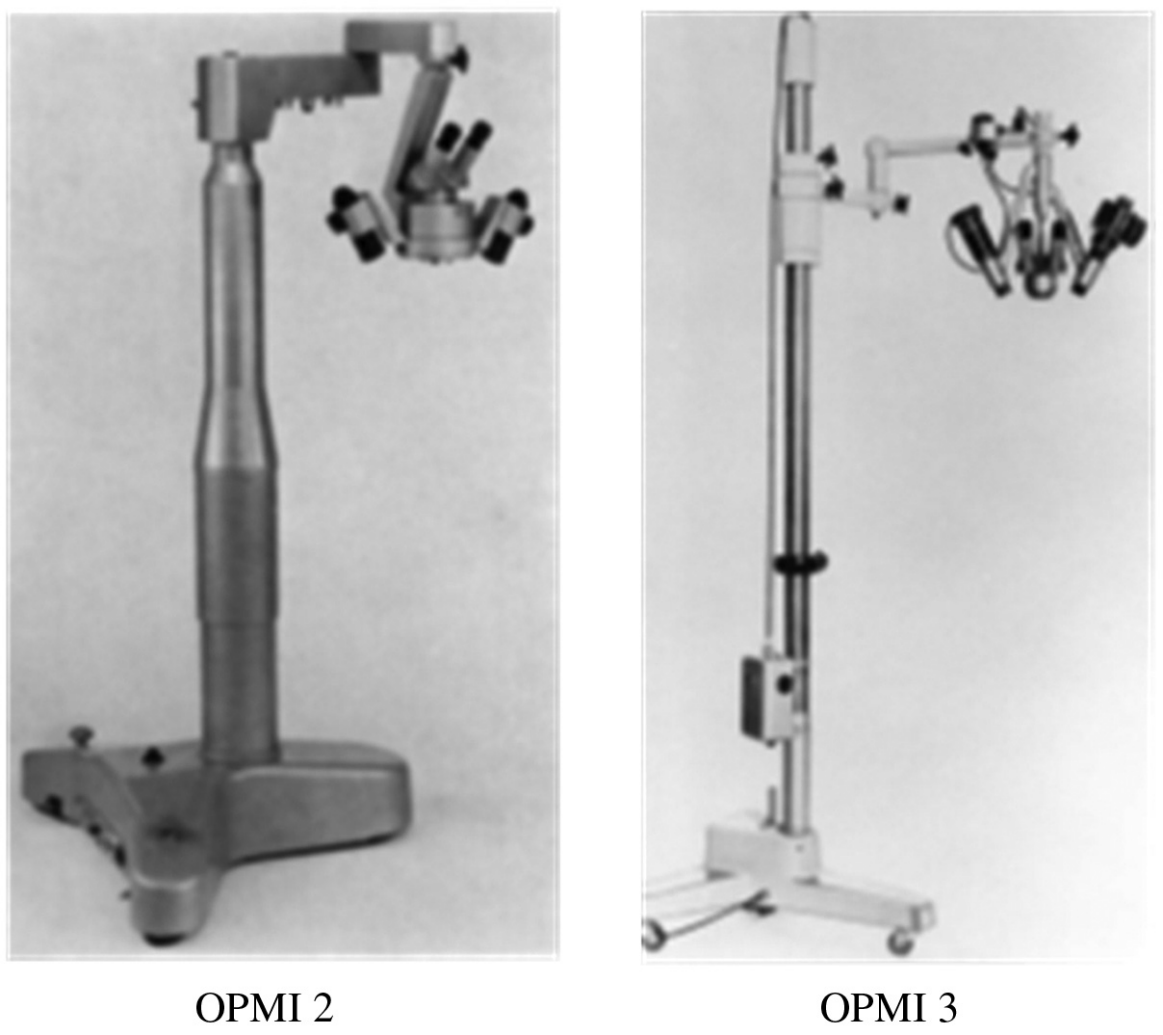
Surgical microscope OPMI 2 and OPMI 3.^10^^,^^15^

Zeiss OPMI 2, which featured motorized zoom and focus, was manufactured in 1965.^19^ The components of OPMI 1 and OPMI 2 were interchangeable. One year later, OPMI 3 was produced,^15^ and then several accessories were attached to it, including a measurement scale in the slit, a rotating prism, suturing reticules in the eyepiece, the rotary Galilean device of the OPMI 1 for magnification switch, and a device to sterilize the microscope.^19^ Figure 4 shows the OPMI 2 and OPMI 3 microscopes.

The OPMI 4 featured a deeper field focusing and 16-mm motion picturing, while OPMI 5 was produced in 1966 to overcome the large size of the Zeiss Diploscope.^19^ The OPMI 7P/H was capable of allowing three surgeons to work simultaneously with its stereoscopic co-observer accessory.^97^ With the increase of observer amount, the light that goes to each observer decreases. Therefore, the OPMI 7P/H applied a high-intensity light source to prevent dimness of the images.

### 2.4 Further Developments

In the later generations of microscopes, a trend of integrating a navigation system and advanced imaging techniques became popular. In 1989, a frameless navigation system based on AR was developed for the surgical microscope in neurosurgery.^106^ In 1994, a frameless navigation device, namely the multicoordinate manipulator,^107^ came into use as an accessory of the OPMI ES neurosurgical microscope. In addition, another navigation system called Multivision was equipped in OPMI Neuro in 2000. OCT as a noncontact optical imaging technique was evaluated with an ophthalmic surgical microscope in 1996.^108^ A surgical microscope was modified for fluorescence imaging in 1997.^6^ In the following 20 years, the AR-based navigation systems and various imaging techniques were gradually transferred into microscope-integrated modules, which have greatly facilitated image-guided surgery, such as fluorescence-guided brain tumor removal,^109^ indocyanine green (ICG)-based intraoperative angiography,^32^ and OCT-assisted keratoplasty.^42^ Images from intraoperative imaging modules or preoperative magnetic resonance imaging (MRI) and computed tomography (CT) images can be displayed in oculars or on monitors to help surgeons to make fast and accurate decisions.^50^^,^^110^ Meanwhile, more imaging modalities such as HSI^43^ and photoacoustic imaging^44^ have been evaluated with the surgical microscope.

**Fig. 5:**
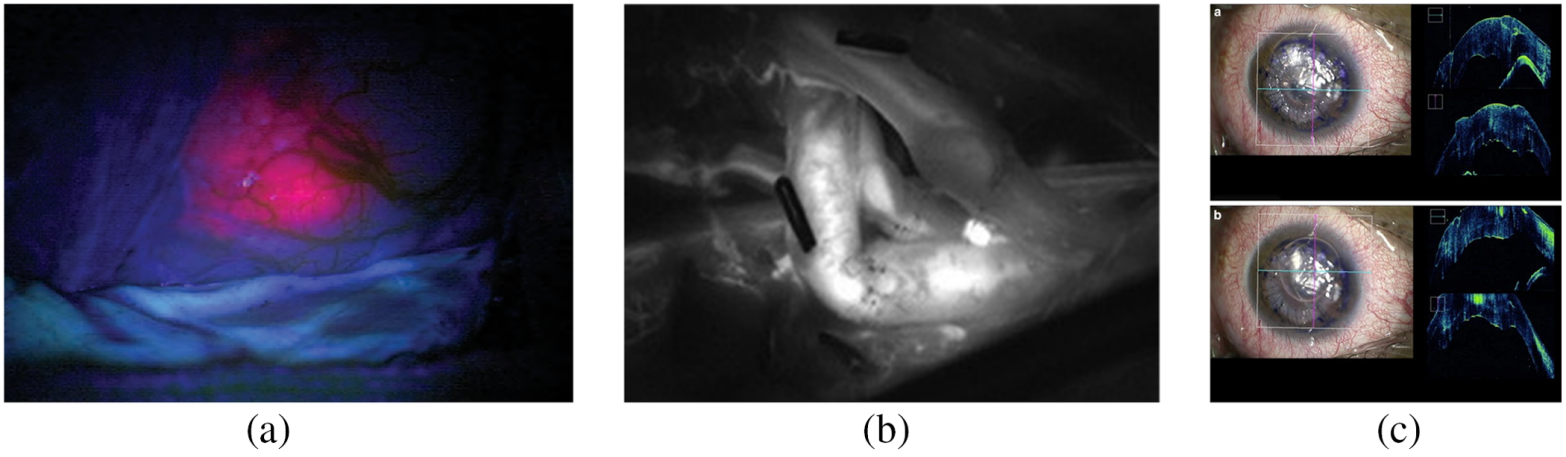
Microscope-integrated (MI) imaging for image-guided surgery: (a) 5-ALA fluorescence-guided tumor resection,^111^ (b) ICG fluorescence for vessel anastomosis,^112^ and (c) microscope-integrated OCT during keratoplasty.^42^

Contemporary microscopes have wide magnification options, sufficient illumination, satisfying balance and stability, and multiple choices of documentation. They are integrated with high-precision automation, as well as sophisticated imaging capabilities, some of them as Fig. 5 shows. All these developments led to a terminology evolution as a “robotic visualization platform,” which indicates the system with significantly more functionalities than a conventional surgical microscope.^60^ The new system uses a camera to capture the whole surgical field and replaces the direct interrogation of the light path by a high-resolution, all-digital way of visualization. This gives the surgeon additional freedom of movement and enables the whole operating room team to appreciate the detailed structures. It is particularly beneficial in minimally invasive robotic surgery so the surgeon can be reassured to operate standing by the robot. In this system, endoscopic assistance with a micro-inspection tool is integrated, which helps the surgeon to observe the deep structures and resection cavities and identify blind spots. Moreover, the surgeon-controlled robots make it possible to “bookmark” a position of the surgical field as well as to visualize the same structure at different angles, providing advantages in time, functionality, and ergonomics. The advent of this new system not only enriches the concept of a surgical microscope with multiple cutting-edge technologies but also unlocks many other improvements and new potential technologies. For example, the simplification of the optical head increases the working distance, providing more space for the use of various microsurgical instruments and the adaptation of imaging modules. The absence of oculars reduces the amount of light required for the assistant observers, thus lowering the intensity of illumination, which in current surgical microscopes may cause damage to underlying tissue. The endoscopic micro-inspection tool offers possibilities for the adaptation and intraoperative use of more imaging modalities such as confocal microscopy and Raman spectroscopy. Moreover, robotic surgery is able to overcome the preexisting limitations of minimally invasive procedures and has led to the possibility of remote surgery. However, the cost of robotic systems greatly limits the popularization of remote surgery. An AR platform developed for virtual surgical collaboration has enabled the cost-effective AR-assisted remote surgery.^113^ If integrated with such platforms, this digital visualization system may promote remote surgery in more clinical applications.

## 3 Technical Aspects of Surgical Microscope

A surgical microscope can be roughly divided into a microscope body, a light source, and a supporting structure,^72^^,^^73^^,^^114^ and each of these is vital for the performance of the microscope. Besides these three conventional parts, modern microscopes have adopted advanced technologies to facilitate visualization and surgical navigation.

The microscope body has all the high-precision optics that provide a clear magnified image with the minimum distortion.^115^ The binoculars mounted on the microscope head offer stereopsis.^116^ Multiple optical ports are open for adaptation of imaging devices such as video cameras or for assistants to share the identical FOV.^25^^,^^117^

The light source is installed away from the microscope to avoid heating the microscope optics or the surgical site.^8^ Commonly used light sources for surgical microscope are xenon light bulbs, halogen light bulbs, or light-emitting diodes (LED).^118^^–^^120^ Illumination from the light source is transmitted to the microscope through a fiber guide, then passes through the objective lens and illuminates the surgical site at a distance that is subject to the focal length.^115^ A good illumination arrangement, such as coaxial illumination, overcomes the shadow and dimness of the FOV.^74^^,^^114^ Meanwhile, the advanced light management ensures the stability of illumination as well as the safety of tissue.

Based on the configuration, there are four types of surgical microscopes: (i) on casters, (ii) wall mounted, (iii) table top, and (iv) ceiling mounted. The on-caster stand is the most popular supporting structure due to its better mobility, but a ceiling mount or wall mount can help with space management.^73^^,^^95^ The supporting structure of a modern microscope has precision motorized mechanics so the microscope can be balanced easily and adjusted flexibly to the right position. It is also a fundamental task for supporting structure to keep the microscope stable. Various controlling methods have been developed for “hands-free” operation, and the improved ergonomics reduces surgeons’ strain during long surgery hours. Furthermore, some new microscope stands have HD display and documentation devices that facilitate the sharing of the operation process.

The adoption and modularization of advanced technologies for image-guided surgery have been actively evaluated in recent decades. On the one hand, the intraoperative imaging modalities have been evaluated with surgical microscopes to provide real-time diagnostic information. The imaging modalities utilize certain properties of human tissue and reveal information that is beyond what human eyes can see, even the deeper structures beneath the tissue surface. To apply these imaging modalities, certain system adaptations have been done for the microscope. The goal of system adaptation is to enable and disable these imaging functions easily without interrupting the surgical workflow or decrease the performance of the microscope. On the other hand, AR has been playing an important role in new generation microscopes, especially with the development of minimally invasive surgery. It helps surgeons relate the preoperative two-dimensional (2D) images with the real 3D surgical site intraoperatively for navigation. AR can work with various image modalities, either preoperative or intraoperative, and overlay the images onto the surgical site so the surgeons do not need to switch their sight between the surgical site and images. In addition, the overlay of images reveals not only the 3D model but also the anatomical structures beneath the patient’s skin. With proper system adaptation, accurate calibration and registration, and convenient visualization methods, AR could greatly aid in the clinic for surgery.

In this section, we provide detailed technical descriptions of a surgical microscope, including its optical system, illumination, mechanical system, and visualization. Advanced technologies employed with surgical microscopes for image-guided surgery will be explained, namely the AR, intraoperative fluorescence imaging, microscope-mounted OCT, HSI, and photoacoustic imaging. The purpose of this section is to provide a comprehensive explanation of the principle of the surgical microscope and how advanced technologies are adopted. It provides references for microscope selection and system development.

### 3.1 Optical System

The optical system of the microscope is the main determinant of the imaging quality that a system can achieve. It is basically a binocular (with eyepieces on top) with a close-up lens, namely the optical components including the objective lens and the magnification changer (or zoom changer).^26^^,^^115^ The focal length of the objective lens fully determines the value of working distance, which is the distance from the objective lens to the point of focus of the optical system. The zoom changer is either a series of lenses moving in and out of the viewing axis or a system that changes the relative positions of lens elements.^115^ The binocular is equivalent to two telescopes hinged together, wherein prisms are used for a compact size of the unit. Stereopsis, which introduces the depth information into the surgeon’s vision, is an important feature brought by binocular and will be discussed in the visualization section.

#### 3.1.1 Magnification

Clinicians from different fields have recognized the usefulness of magnification.^10^^,^^25^^,^^62^^,^^72^^,^^82^^,^^103^^,^^114^^,^^121^

The total magnification ($M_{\text{total}}$) of a surgical microscope is determined by all the four optical components in the microscope, namely the focal length of the objective lens ($f_{\mathrm{OBJ}}$), zoom value ($M_{\mathrm{ZOOM}}$), the focal length of binocular ($f_{\mathrm{TUBE}}$), and the magnifying power of eyepieces ($M_{\mathrm{EP}}$),^115^ as Eq. (1)

$$M_{\text{total}}=\frac{f_{\mathrm{TUBE}}}{f_{\mathrm{OBJ}}}\times M_{\mathrm{EP}}\times M_{\mathrm{ZOOM}}.$$ (1)

Magnification of modern surgical microscopes varies from 4× to 40× ^10^^,^^73^^,^^122^ and is usually selected through a manual or motorized magnification changer. The zoom value is usually 6:1 but can be as high as 8:1.^123^ For some microscopes, an additional magnification multiplier is applicable, which provides 40% more magnification.^124^ Resolution measures the acuity improved by magnification. It is the ability of an optical system to distinguish two separate entities.^74^ Human eyes have an inherent resolution of 0.2 mm^125^ but with 20× magnification, it can be increased to 0.01 mm.^126^ This can add more confidence to surgeons, enhance the advanced surgical skills, and enable the use of many fine surgical instrumentations when they operate on fine anatomical structures.^121^

#### 3.1.2 Optics

The design of optics is vital to the image quality of a surgical microscope. Aberration is an inherent property of optical systems, and it causes the blur or distortion of images, which is adverse to the desire for a clear view. Monochromatic aberrations such as spherical aberration, coma, and astigmatism can be corrected but usually only for one color.^127^ Chromatic aberration is a failure of a lens to focus all colors to the same point, because of which images show color fringes and lose sharpness. Chromatic aberration correction is necessary for optics in a surgical microscope not only because of the wide-band light source used but also due to the image enhancement in cameras, such as sharpening and edge enhancement, which enhances the image edge as well as the color fringes.^128^ Achromatic lens, which is a combination of converging and diverging lens elements, was employed in early surgical microscopes to correct the primary spectrum, leaving the secondary spectrum being the main factor limiting the image quality.^16^^,^^19^ The apochromatic lens is the answer to that problem. It not only corrects for two wavelengths (red and blue) to reduce spherical aberration but also utilizes the exceptional quality optical materials that have unusual and desirable characteristics to reduce chromatic aberration for three wavelengths (red, green, and blue).^19^^,^^129^

#### 3.1.3 Focusing

Focusing is essential for a clear view. Surgeons would want the surgical site to be in focus throughout the surgery. However, the shape of organs or the deep cavities makes it impossible for the whole surgical site to be perfectly on the focal plane. Depth of focus, in other words, depth of field (DOF), is a term that indicates the area in front of and behind the point of perfect focus where the sharp focus is maintained. It depends on many factors, including but not limited to the quality of optical design, the size of objective lens aperture relative to the focal length of the objective lens, and the magnification of the object, and it is reciprocal of the resolution.^115^ A good surgical microscope should have an adequate depth of focus without sacrificing too much resolution to keep the scene sharp. Another important term is parfocal, which means an optical system can stay in focus even with magnification changes.^130^ Due to the need of switching magnification during surgery, a surgical microscope being parfocal saves surgeons from repeated refocusing.

Microscopes need to be well focused before the operation, and when the position of the microscope is adjusted during surgery, refocus is needed. A fast focusing capability can save setup time for surgery. Various methods have been proposed for the automatic focusing of the surgical microscope. Nohda^131^ proposed an automatic focusing device for a stereoscopic microscope, which detects the position of the reflected image of infrared LED (IR-LED) using a focusing screen. The positions of the IR-LED and the focusing screen are conjugate with the in-focus position of the sample; hence, the reflected image of the infrared diode is at the center of the focusing screen when the sample is in focus. Jorgens and Faltermeier^132^ proposed using the interaction of an active light-projecting system and a passive video system to focus on both covered and uncovered objects illuminated by the transmitted and reflected lights. Vry et al.^133^ proposed a high-precision optical arrangement for stereomicroscope autofocusing, where a cylinder optic is employed to project a bar-shaped mark onto the object. Many current microscopes are equipped with fast autofocusing optics, which uses two laser beams acting as a focusing reference to find a focus point rapidly. The focus point works for not only the main viewing position but also the assistant position and camera. Furthermore, methods have been developed to maintain the surgical microscope in focus at different viewing points. For example, Heller^134^ proposed a mechanical control unit for a surgical microscope support stand, and the unit constrains the microscope to move along a spherical surface so the focusing status can be maintained.

### 3.2 Illumination System

Illumination is another key factor besides the optical system for the imaging quality of a microscope. Successful surgical illumination has four key factors, namely the luminance, shadow management, volume of light, and heat. A bright view of the whole surgical site throughout the surgery is always desired. The original illuminator in the earliest surgical microscopes was an independent bulb externally mounted on the side of the microscope. Light transmitted to the surgical site likely creates shadows, and thus illumination of deep cavities was hardly possible.^20^^,^^115^ Modern microscopes have adopted high-power light sources with stable light intensity and close-to-sunlight color temperature.^73^^,^^122^^,^^135^ With the built-in coaxial illuminator, light is rerouted to the viewing axis and projected down through the objective lens.^115^ It is beneficial to remove shadows in cavities and complex structures and especially cause a red glow of the retina that assists cataract surgery. Light management methods have also been developed to guarantee a stable and relatively safe illumination regardless of any change of magnification or working distance. In addition, some modern surgical microscopes offer an option to set up various lighting profiles for different tissues, so by controlling the light sensitivity of the integrated recording camera, a change of color temperature may appear as images of the surgical field are displayed on the monitor.

Despite numerous advantages of surgical microscope illumination, it is still worth noting that many up-to-date neuro and spine surgical microscopes use light sources of the highest intensity to provide the best brightness and clearness for human eyes regardless of magnification and working distance. However, the high power can damage the underlying tissue. Though manufacturers of surgical microscopes provide safety warnings of possible damage, specific settings of the illumination are not regulated.^136^ Nevertheless, the International Organization for Standardization (ISO) 10936-2 standard and the American National Standards Institute (ANSI) Z80.38 standard have set requirements for the maximum retinal exposure limit and the stability of light intensity of ophthalmic surgical microscopes.^137^^,^^138^ Besides, the International Electrotechnical Commission (IEC) has set general requirements for the characteristics of surgical lighting, including a central illuminance of 40,000 to 160,000 lux, a color rendering index between 85 and 100 Ra, and a color temperature of 3000 to 6700 K. This standard does not apply to the lights for surgical microscopes since they are excluded as “special purpose lights with different conditions of use,” but these requirements may offer a general idea for the requirement of surgical illumination.

#### 3.2.1 Light source

Except for the traditional incandescent bulbs used in old surgical microscopes, there are mainly three types of light sources, i.e., xenon lamp, halogen lamp, and the LED. LED can provide illumination in the visible wavelength range with good brightness, good stability, longer life, less power consumption, and extremely low heat; therefore, it is preferred in many ophthalmic and ENT microscopes.^139^ However, LED as a surgical light source also has disadvantages: the higher color temperature and narrower wavelength range make the light not as close to sunlight; its spectrum is insufficient for fluorescence-guided applications especially ICG imaging, where an excitation light in the NIR range is needed; moreover, it is not easy to replace.

Xenon lamp and halogen lamp are two options to address these needs. Xenon lamp emits light with a broad spectrum from ultraviolet (UV) (185 nm) to infrared (2000 nm). The spectrum is relatively smooth in the visible range, but it has some spikes in the near-infrared (NIR) range. Xenon light has a color temperature of 4000 to 6000 K, which is similar to sunlight. Therefore, the bright-white light is able to offer a naturally colored view of the anatomy. Halogen lamp also covers a wide and continuous spectrum including visible and NIR light, but it has a slightly lower color temperature (3200 to 5000 K), which means the light does not look as “white” as xenon light. Both xenon and halogen lamps can provide a stable illumination with DC regulation power employed. Nevertheless, the surgical microscopes do not use all the wavelength range of xenon lamp or halogen lamp. Actually, UV light and infrared light above ∼1100  nm are filtered out for surgical microscopes to avoid various possible damages to the patient’s skin or eye caused by exposure in this wavelength range.^140^^,^^141^ Xenon and halogen light sources are commonly used in neurosurgical microscopes because of the need for intraoperative fluorescence imaging. They are also utilized in some ophthalmic and plastic microscopes. For example, some ophthalmic microscopes may employ a dual-illumination system combining LED and halogen for Red Reflex and normal illumination. Both halogen lamps and xenon lamps emit much heat. Therefore, in a surgical microscope, the light source is installed away from optics, and a fiber guide is used to transmit light from the light source to optics without carrying the heat.

#### 3.2.2 Illumination arrangement

**Fig. 6:**
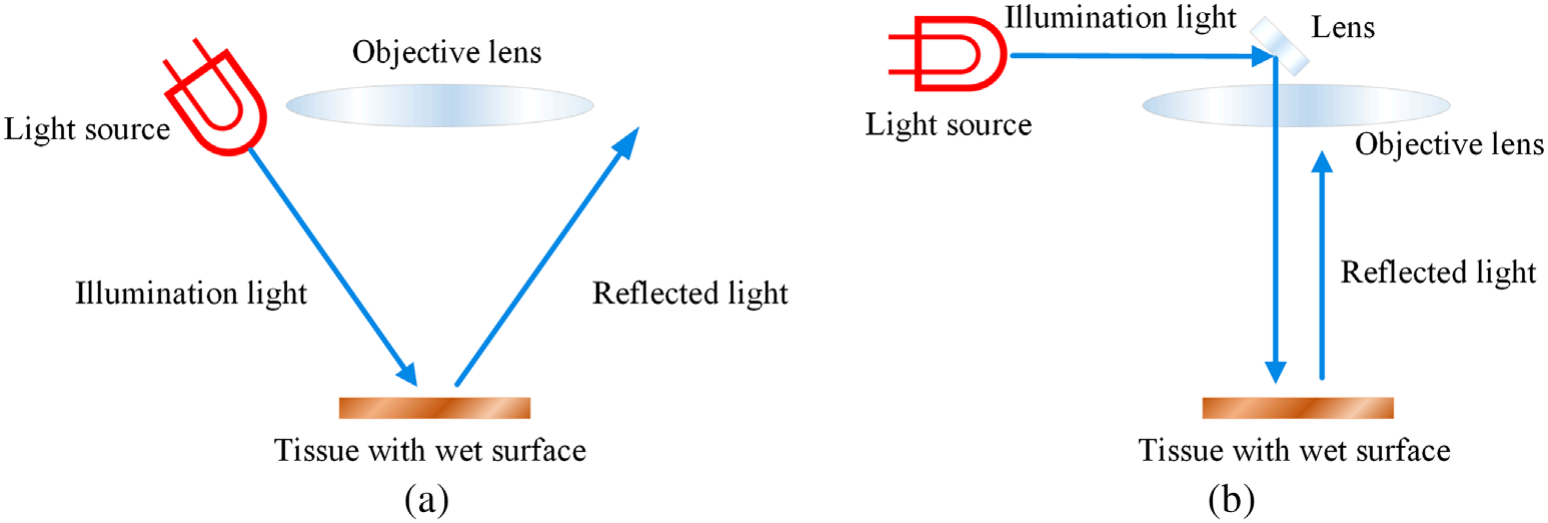
Illustration of coaxial illumination and comparison with side illumination: (a) side illumination and (b) coaxial illumination.

The tissue surface being viewed under a surgical microscope during operation is usually wet and highly reflective. The light that comes from an angle can be easily reflected away and cause a dark view, as Fig. 6(a) shows. Coaxial illumination is the solution to this situation. Different from lateral illumination where light comes from the side, coaxial illumination matches the optical axes of illumination and visualization (lens).^142^^,^^143^ Illumination from the light source that locates on the side is diverted and projected almost parallel to the axis of the lens, as shown in Fig. 6(b). Therefore, light vertically illuminates the tissue surface and is reflected directly to the lens, not having much loss. Coaxial illumination reduces the diameter of the illuminated area,^144^ moreover, it can be directed into narrow and deep cavities, which is helpful for neurosurgery, ENT surgery, and endodontics.^72^^,^^144^

**Fig. 7:**
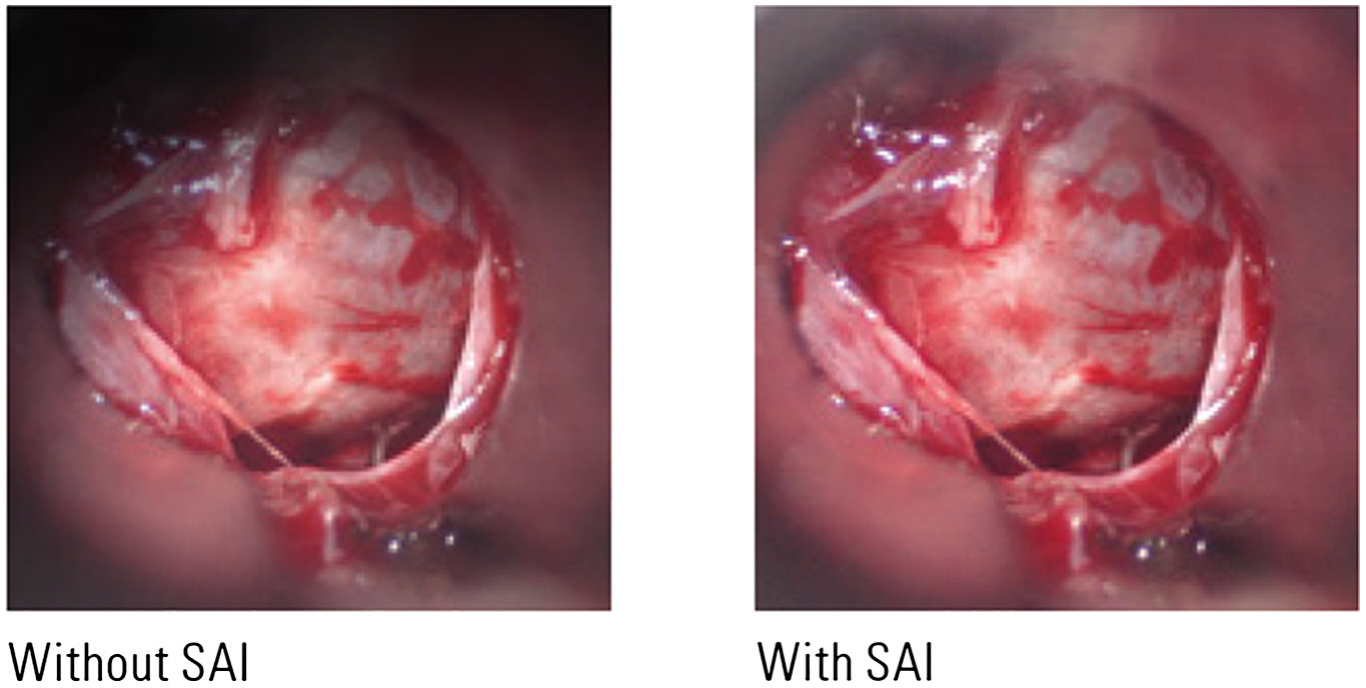
Comparison of illumination effect with and without SAI.^124^

The light path for coaxial illumination in nonophthalmic surgical microscopes, such as neuro or ENT microscopes, usually forms a small angle with the observation axis in the range of 6°.^144^^–^^146^ In some contemporary surgical microscopes, it is called small angle illumination (SAI),^124^ which provides a concentrated and evenly distributed light beam, a bright view, and an improved depth perception, as Fig. 7 shows. With SAI, the shadow that appears at the edge of the viewing field is significantly reduced. Illumination with an even smaller angle is important when it comes to certain ophthalmic interventions especially cataract operations, where the vertically impinging light gets diffusely reflected by the fundus and the pupil under operation shines reddish, which is called red reflex.^144^^,^^145^^,^^147^ The production of red reflex requires a small angle between the illumination beam path and observation beam path, in the range of 0 deg to 2 deg.^144^^,^^146^ Although the red reflex helps under certain circumstances, it does not help as much in revealing good plasticity without the shadows on the structures in the interior eye.^145^ Therefore, both types of illumination, namely the 6 deg and the 0 deg illumination, are usually equipped in ophthalmic surgical microscopes.

#### 3.2.3 Light management

**Fig. 8:**
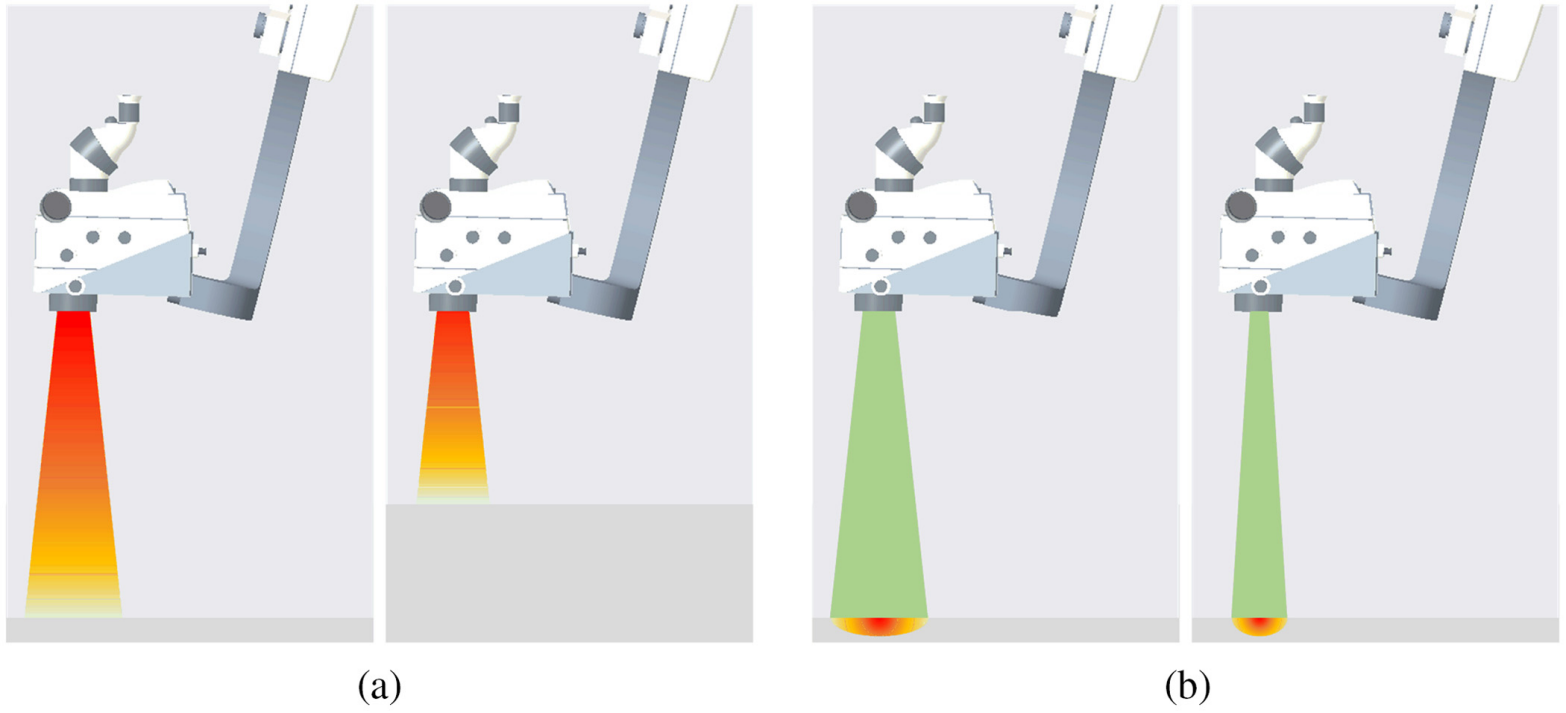
Light management related to working distance and spot size: (a) automatic adaption of light intensity with decreased working distance and (b) automatic adjustment of the illuminating area with increased magnification.

A desirable illumination for a surgical microscope should provide a stable brightness for the viewing area regardless of the change of working distance or magnification. In fact, irradiance (irradiation of a surface, $W/m^{2}$) of a microscope light source increases with decreasing spot size and decreasing working distance.^136^ With an unchanged illumination setting, the increased working distance can cause insufficient irradiance, while the decreased working distance excessive irradiance. Insufficient irradiance makes the view unclear, while excessive irradiance may cause soft tissue burns.^148^ Similarly, decreased magnification, which enlarges the FOV, may lead to the dimness, while increased magnification may burn tissue outside of the FOV. To address this issue, many contemporary surgical microscopes are equipped with smart light management, which adjusts light intensity automatically with the change of working distance [Fig. 8(a)] or magnification [Fig. 8(b)].

### 3.3 Mechanical System and Automation

The structure of the whole surgical microscope system can be delicate and complicated. It assembles every part of the system and makes them work together harmoniously. A well-designed system can assist surgeons with good stability, sterility, easy operation, as well as comfort. Mechanical stability is the second most important criterion in selecting a surgical microscope.^130^ The drift or vibrating of a microscope after positioning distracts surgeons’ focus on the surgical site. Therefore, superior suspension and balancing mechanisms are important. Microscope draping is a necessary requirement for sterilization in the OR. A good draping design saves the setup time for the microscope and avoids the effect of glare.^149^ Various controlling methods have been developed to enable hands-free operation for surgeons.^134^^,^^150^^–^^152^ Moreover, different parts of a surgical microscope have been designed to improve its ergonomics and maneuverability.^60^^,^^153^ This section will discuss some important features that affect the operation of surgical microscopes involving its mechanical design and electrical automation.

#### 3.3.1 Balancing and positioning

As is known, surgical microscopes should be quick and effortless to move and remain stationary once the position is established.^130^ Balancing of the forces and moments from all directions should be achieved, otherwise, brakes or bracing devices are needed to hold the microscope in its position.^154^ Many suspension structures and balancing apparatus have been developed for a fast and reliable balancing of microscope.^154^^–^^160^ Modern surgical microscopes have made it an easy and time-saving process to balance. All six axes can get fully balanced with two pushes of a button, and intraoperative rebalance can be quickly and accurately accomplished with a single push of button on handgrip.

In recent years, a robotic autopositioning feature has been added to state-of-art surgical microscopes.^161^^–^^163^ The robotic ability enables the microscope to orient the angle or change its focal length so surgeons can target a specific point of interest, which is probably identified in a preoperative imaging study. Oppenlander et al.^162^ developed the automatic positioning movement control with three options. The first option is “auto lock current point,” which makes surgeons lock onto a target by changing the angle and focal length of the microscope to keep it in focus at one point while being manually moved. The second option is “align parallel to plan,” which positions the microscope to a preset angle and focus without any need to adjust the microscope. The last one is “point to plan target,” which automatically adjusts the focus on a predefined target. In a newly developed robotic visualization system, two robotic positioning features, namely “point lock” and “position memory,” have brought many advantages in time, functionality, and ergonomics.^60^ With “point lock,” the microscope head stays in focus when being manually or automatically moved during surgery, so the surgeon can visualize different angles of the same structure. “Position memory” makes the system able to “bookmark” positions and transit quickly and smoothly back to these positions with no need to rediscover. In some circumstances where the scope needs to be moved around to observe different structures or be temporarily removed to get an x-ray, “position memory” can save plenty of time getting the scope back to the same position. Previously, it was reported that around 40% of surgical duration was spent on adjusting the microscope. But with all these robotic positioning features, the surgical duration can be greatly reduced.^60^

#### 3.3.2 Draping

Microscope drape is a very thin, transparent, and heat-resistant plastic film that houses the whole surgical microscope, and it includes a transparent optical lens enclosing objective lens and ocular-housing extensions.^164^^–^^166^ The drape is seamed for sterile packaging to assure the microscope sterility during surgery.^167^ To save the setup time of a surgical microscope, instant readiness is required. Meanwhile, the drape must have adequate ocular pockets, not reduce the working distance, not interfere with surgeons’ operation or obstruct the view. Bala^168^ invented a microscope drape assembly, which has four ocular pockets for different needs and does not affect the working distance or visualization by locating the objective lens window support within the objective lens barrel.

Glare is one problem that comes with draping and illumination. As light passes through the objective lens and illuminates the surgical field, some of the light would be reflected by the lens cover on the drape, which can cause chromatic and spherical aberrations.^149^ Removing the cover, however, can cause the contamination of surgical instruments. A dome-shaped objective lens cover^169^ can not only reduce the reflection but also compromises the magnifying performance. Surgical microscope manufacturer has brought up a solution by replacing the sterile lens cover with a slanted one, and another attempt solution is to include the slanted lens cover in the sterile microscope drape. Both methods can eliminate glare, with the price of increased costs or system complexity. Langley^149^ has invented a glare elimination device for surgical microscopes. The device includes three parts: the first part is to be attached to the surgical microscope, the second part is for the sterile drape, and the third part is a body to connect the other two and provide an angular offset. The device can be semipermanently attached to a surgical microscope with more convenience. Weaver et al.^170^ proposed an apparatus that provides a secondary holder for a cover to be applied to the objective lens barrel. The additional cover can be rocked and rotated easily to a position where the view is not affected by glare.

#### 3.3.3 Control

Surgical microscopes can be controlled in various ways to facilitate easy use of the microscope and to free surgeons’ hands during surgery. Contemporary surgical microscopes are often equipped with footswitch devices^171^ for generating control commands, touch-screen^58^^,^^172^^,^^173^ for operation mode selection or switching images intraoperatively, or joystick control^174^ for highly precise micropositioning. Mouth switch^175^^–^^177^ is a commonly employed controlling method. Surgeons can use the mouth switch to change signals simply by holding the levers with a mouth, in which way operation errors can be reduced, even with a large number of functions to control.^175^ Eye controlling is another trend for surgical microscopes. Charlier et al.^178^ proposed an eye-controlled surgical microscope, which used an IR-LED to illuminate the surgeon’s eye and a charge-coupled device (CCD) sensor to detect the reflected infrared light from the surgeon’s eye for movement tracking. Similarly, Roduit et al.^152^ proposed an eye-guided controlling technology, which used a CCD camera mounted on the right ocular of a microscope and continuously monitored the surgeon’s eye. With the eye-guided control function, surgeons can use their eyes to perform multiple tasks including access to built-in data display, laser aiming, and control of autofocusing. Voice control is a sterile remote control that facilitates operator in either sterile or nonsterile region and does not require the operator to take action.^151^ Furthermore, Pitskhelauri et al.^150^ developed a device named Mari, which allows hands-free utilization of surgical microscopes. The device was attached to the eyepieces of a surgical microscope, and operators can use the joystick and electric switch to do multifunction control of the microscope.

#### 3.3.4 Ergonomics and maneuverability

**Fig. 9:**
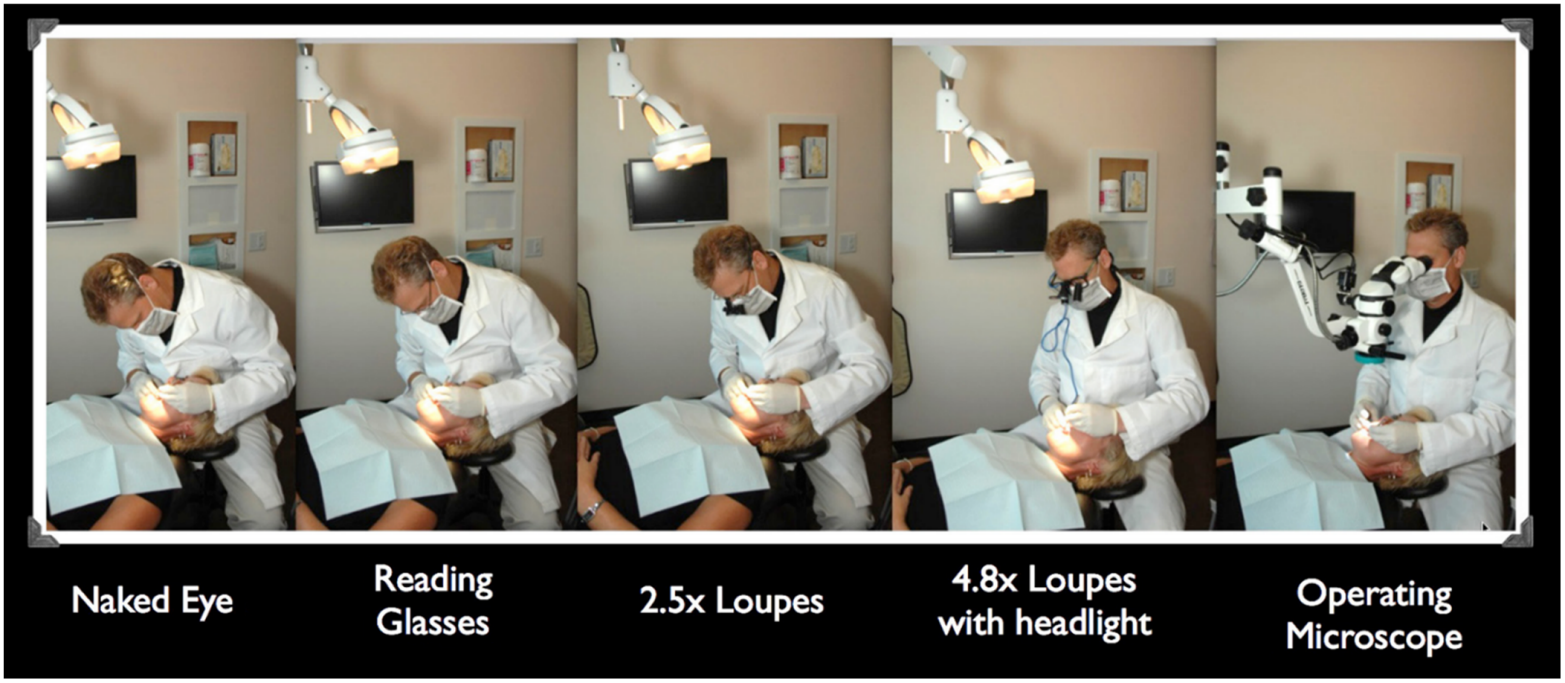
Illustration of improved ergonomics with surgical microscope.^181^

Except premium optics, good illumination, and various image-guided surgical functions, one nonnegligible benefit of surgical microscope compared with traditional loupes is the ergonomics,^25^^,^^73^^,^^130^ which guarantees a comfortable and flexible working position for surgeons and reduces the risk of back and neck musculoskeletal injuries.^25^ Meanwhile, maneuverability is valued for the simplification of microscope operations.^130^ Therefore, the microscopes of the time are equipped with a full range of movement and tilt of the optics carrier, as well as a selection of binoculars with full 360-deg rotation for different heights and positioning needs. Some microscopes have large HD monitors so that surgeons can all work with an upright position. Eye-to-object distance^115^ indicates the distance from the observer’s eye to the focus point of the microscope. Surgeons are likely to be more comfortable with a longer eye-to-object distance. In addition, new designs of surgical microscopes are trying to provide longer working distances up to 600 mm^179^ to offer better ergonomics, easy maneuvering, and more working space that allows long instruments. For example, Horizontal Optics Technology, which is employed in the state-of-art surgical microscope, enables a compact optics carrier and further ergonomics.^180^ Figure 9 shows how a surgical microscope can improve ergonomics for an endodontic surgeon as an example.

### 3.4 Visualization System

Clear and bright visualization of the surgical site is the ultimate goal of using a surgical microscope. Except the good image quality provided by high-precision optics and sufficient illumination, the stereoscopic view that offers depth information is another non-negligible benefit of the binocular surgical microscope. Despite that stereopsis is the result of optical design, it influences how surgeons obtain information from and feel about what they see.

Users of surgical microscopes can observe the surgical site in various ways. A microscope head usually has one main observation port and one rear or lateral port for co-observers, who can be assistants, students, or trainees. Cameras^182^ or other imaging systems^183^ can also be adapted to these optical ports for video recording or photography of the ongoing surgery. All optical ports offer an identical FOV, which beats surgical loupes and enables “cosurgery.”^25^^,^^184^ With the image injection technique, not only the white-light image of patient tissue but also pre- and intraoperative images can be overlaid accurately with the white-light image for navigation.^57^^,^^58^ HD display^105^^,^^185^ and 3D display^22^ have been employed in the surgical microscope for sharing of the view with high resolution and enlarged stereoscopic images. Other visual methods, such as using smartphones for recording and virtual reality (VR) headsets for visualization, have also been developed.^186^ With the advanced technologies applied, surgical microscopes can help surgeons see much easier than ever before.

#### 3.4.1 Stereopsis

Stereopsis is a key feature of binocular surgical microscopes. While the monocular depth cues lie in perspective projection, occlusion, size, shading, and motion parallax, the stereoscopic depth is based on the slight disparities between two images presented to two eyes.^187^ Stereo microscopes use two afocal relay zoom lens systems for the two channels of a binocular tube, and their axes are parallel to and offset from the axis of the objective lens.^188^ The light coming out of the objective lens is divided into two parts and forms two slightly different images into two channels. In surgery, especially when working with magnification, perspective, and size cues may be lost; therefore, the stereopsis brought by binocular is essential to provide a 3D impression of the surgical field. The depth information can aid the detection of diagnostically relevant shapes, orientations, and positions of anatomical features, especially when monocular cues are absent or unreliable.^187^ For example, it is vital for dentists to construct 3D structures in patients’ mouth^74^ and for neurosurgeons to understand complex volumetric relationships of neuroanatomical structures.^60^

An optical design that enhances stereo visualization for surgical microscopes is FushionOptics technology,^189^ which sets two separate beam paths in the optical head, providing the DOF and high resolution, respectively. The two paths are then merged in the observer’s brain into a single, optical spatial image. Because of this combination of depth and resolution, the interruptions for refocusing can be avoided.

#### 3.4.2 Share of view

There is usually more than one observer during surgery, which makes the “share of view” an important and necessary feature for surgical microscopes. In some procedures, meaningful assistance has to be given by a cosurgeon sharing the same view with surgical microscope.^25^^,^^117^^,^^190^ It aids not only assistance but also teaching and participation of trainees.^25^ The simplest way to share the identical surgical view is to use an optical splitter to split the light into two eyepieces.^142^ Nowadays, surgical microscopes can have multiple optical ports for the main observer, assistant observer, and external cameras. Some models have integrated HD cameras and monitors so the whole team can share the view on the screen.^19^^,^^25^^,^^117^^,^^191^

#### 3.4.3 High-definition display and 3D visualization

Many new surgical microscope models, especially neurosurgical microscopes and ophthalmic microscopes, are equipped with HD video cameras and large HD monitors, so subtle details can be viewed more clearly and shared by the whole team.^56^^,^^58^ In addition, 3D screens, which employ passive linear polarization technology, have been brought to the operating room to deliver depth perception.^22^^,^^192^^–^^194^ Observers need to wear goggles to have a real-time 3D view, which gives a realistic appraisal of certain features. It was reported that screens possibly offer better contrast of the visual field than eyepieces and image injection in some cases.^58^ Moreover, utilizing screens enables the heads-up display, which is beneficial for surgeons’ spinal health during long procedures.

**Fig. 10:**
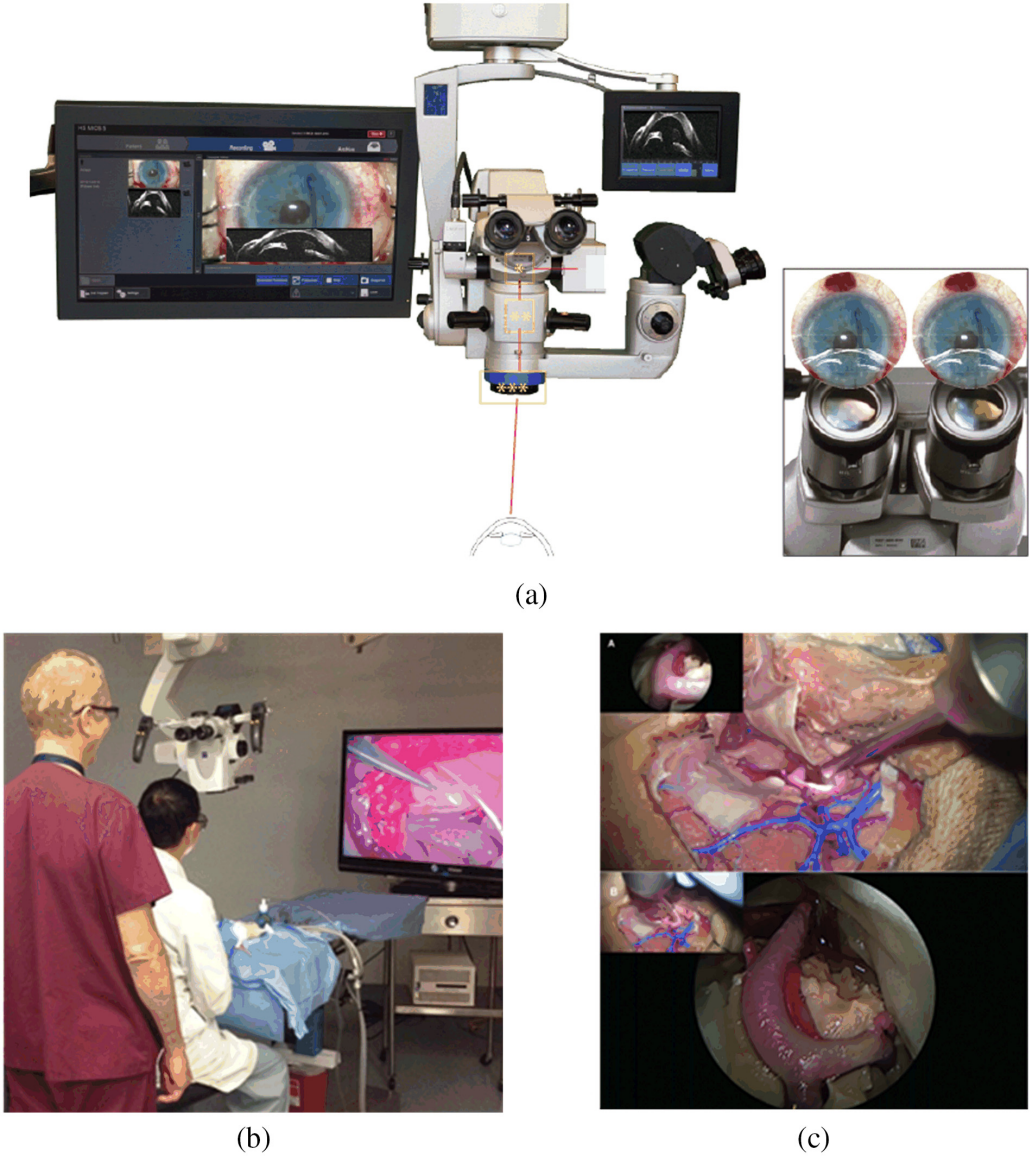
Screens for visualization during surgery: (a) intraoperative OCT images shown separately on 6.5-in screen, with white-light image simultaneously on 21.5-in screen, and injected in oculars,^58^ (b) surgeons using the 3D display in a seated position with goggles,^22^ and (c) picture-in-picture 3D visualization of endoscopic assistance.^60^

A screen can show not only the white-light image of the surgical site but also other images, such as intraoperative OCT images, for surgical guidance. The images can be shown separately, overlaid on the white-light image,^58^ or even in picture-in-picture endoscopic assistance view^60^ for endoscopic microinspection tools, as shown in Fig. 10.

### 3.5 Augmented Reality

During surgeries, especially neurosurgeries, image-guided surgical navigation systems are critical for surgical outcomes.^195^^,^^196^ Although surgeons can obtain the knowledge of anatomical structures of patients via preoperative images such as CT images, they have to work with radiologists to build up the anatomical structure model in mind preoperatively.^197^ Moreover, there is difficulty for them to relate the preoperative x-ray information to the appearance of the surgical view.^63^ Surgical navigation systems that only display 2D images on screens require that surgeons perform the 2D-to-3D transformation themselves in mind, and surgeons need to switch views constantly between screen and patient, which disturbs the surgical workflow.^196^^,^^198^^,^^199^

AR can be very helpful with preoperative planning and intraoperative surgical navigation. It provides the visualization of anatomical structures beneath human skin intraoperatively by overlaying segmented preoperative images to the corresponding area on the human body. Attempts to apply AR in neurosurgery, general surgery, orthopedic surgery, maxillofacial surgery, otolaryngology, and cardiovascular and thoracic surgery have been proved successful and promising.^200^ The concept of AR is to overlay real-life structures with artificial elements.^196^^,^^197^^,^^201^ Not only the 3D model but also the detailed anatomical structures can be illustrated by the overlaid image. The images to be overlaid with the real-life environment can be CT, MRI, and angiography,^52^^,^^63^^,^^198^^,^^199^^,^^202^^–^^207^ ultrasound,^199^^,^^201^ NIR fluorescence,^54^^,^^208^^,^^209^ or OCT images,^51^ depending on the operation target. The representation of virtual images can be surface mesh, transparency, texture map, or wireframe.^203^^,^^206^^,^^210^ AR differs from VR, with which the user is surrounded by a virtual world (immersion) and interacts with the virtual world (presence).^211^ In surgery, VR refers to a virtual patient on a physical model of the pathology, surgical instruments, and connectors of all VR–reality interfaces.^197^

There are three core components of AR.^212^ The first one is a virtual image or environment, which refers to the computer-generated 3D reconstruction of a subsurface target with color or texture-coded differentiation between anatomical structures. The other two core components are the registration of the virtual environment with real space, and the display technology to combine the virtual and real environment, respectively. In clinical use, the overlaid images can be displayed on many surfaces: monitors,^213^^–^^216^ optics (i.e., microscope),^50^^–^^52^^,^^57^^,^^63^^,^^199^^,^^205^^,^^217^^,^^218^ head-mounted devices (i.e., smart glasses),^219^^–^^222^ semitransparent surfaces,^223^^–^^225^ and the patient.^207^^,^^226^^–^^228^ Using AR with a surgical microscope facilitates navigation with multiple magnifications and would not require additional AR system cost since surgical microscopes are available in most modern operating rooms.^210^ In this review, we focus on the allocation of AR with surgical microscopes.

#### 3.5.1 Augmented surgical microscope

The microscope-based AR systems have been found particularly useful in neurosurgery, which is the earliest adopter of AR.^210^^,^^212^ The majority of applications for recent neurosurgical AR is tumor resection, followed by neurovascular surgery and spinal procedures.^7^ For tumor resection, AR with the segmented CT or MRI image helps with the margin definition during surgery, AR overlay of volumetric CT/MRI data with no additional surgical time or complications reduces both intensive care unit and hospital length of stay.^229^ AR for vascular neurosurgery has focused on the augmentation of stereomicroscopes,^212^ with either fluorescence from intraoperative ICG angiography or segmented preoperative CTA/MRA/digital subtraction angiography (DSA).^57^^,^^209^^,^^230^^,^^231^ The overlay of the target vasculature optimizes craniotomy placement, dural opening, and skin incision.^57^^,^^212^^,^^230^^,^^231^ Moreover, it is significant that the microscope-based AR system does not require the bayonet pointer typical for common neuronavigational systems.^210^

The first augmented monoscopic surgical microscope,^106^ which was used for cranial surgery, was proposed in 1985. The segmented 2D preoperative CT images were displayed in monocular and were registered to the operating table using an acoustic localizer system, but this system was unable to track tools in real time. The first augmented stereoscopic operating microscope was proposed in 1995.^63^ It achieved multicolor displaying of segmented 3D cross-sectional MRI/CT data into both microscope oculars via a beam-splitter, either as solid or wire-mesh overlays. Fiducials on the skin surface were used for patient registration. The overlay accuracy was 2 to 3 mm, and the interactive update rate was 2 Hz. The system used an LED-based 3D localizer for calibration, microscope pose tracking, and patient tracking. Therefore, the microscope was capable of free movement while maintaining the overlay accuracy. Later in 2000,^232^ several improvements were achieved to the system, including automated calibration, the bone-implanted fiducial added for registration, and the locking acrylic dental stent for patient tracking. The clinical overlay errors were 0.5 to 1.0 mm on bone fiducials and 0.5 to 4.0 mm on target structures. In 1996,^233^ the S.M.N. system developed by Carl Zeiss (Germany) as a neuronavigational system was first installed at Rennes Pontchaillou Hospital. The system was integrated with an OPMI ES surgical microscope, and it utilized a 3D optical localizer for tracking, which was comprised of infrared emitters and three linear 1D cameras. Fiducial markers such as bone or skin markers were used for registration. The virtual images were either MRI/CT slices or 3D rendered images, and they were both injected into the microscope ocular and displayed on the monitor. Inside of the microscope ocular, surgeons had access to menus, so they could interact with the user interface.

#### 3.5.2 Technical implement of augmented surgical microscopes

The procedures of an augmented surgical microscope include calibration of the optical system, tracking, registration, and display.^232^ The AR accuracy mostly depends on the accuracy of the tracking technology, the registration procedure, the camera calibration, and the image scanning device (e.g., CT or MRI scanner).^198^ Registration is the process of relating two or more data sets to each other to match their content.^234^ It is essential for augmented surgical microscopes because the system is very sensitive to misalignments of the virtual image and the real environment (the patient tissue). Calibration of binocular optics and registration limit application accuracy mostly.^232^ The term tracking for AR refers to the pose estimation of objects in real time.^234^ The display of an augmented surgical microscope mostly refers to the image injection into microscope oculars but also the monitors that are equipped in some new-generation surgical microscopes.

Early image overlay in a microscope-based neuronavigational system injects 2D contours into one eyepiece.^202^ Therefore, surgeons needed to either scroll through different image planes and merge them in mind or look away from the microscope for a 3D impression.^205^ The conceptual description of the AR idea of 3D stereoscopic overlay of the operating field in a surgical microscope appeared in 2003 proposed by Aschke et al.^205^ The process is divided into two phases, namely the preoperative phase and the intraoperative phase. In the preoperative phase, calibrations under different magnifications are accomplished to obtain lens error values. Patient image data, e.g., from MRI, fMRI, or ultrasound image are segmented manually and a reference model is generated. Then, the image data and the reference model are matched to patient’s anatomy by the registration process. In the intraoperative phase, new intraoperative image data such as intraoperative MRI are registered to the reference model, and the coordinates of intraoperative image data are utilized to segment edges.

**Fig. 11:**
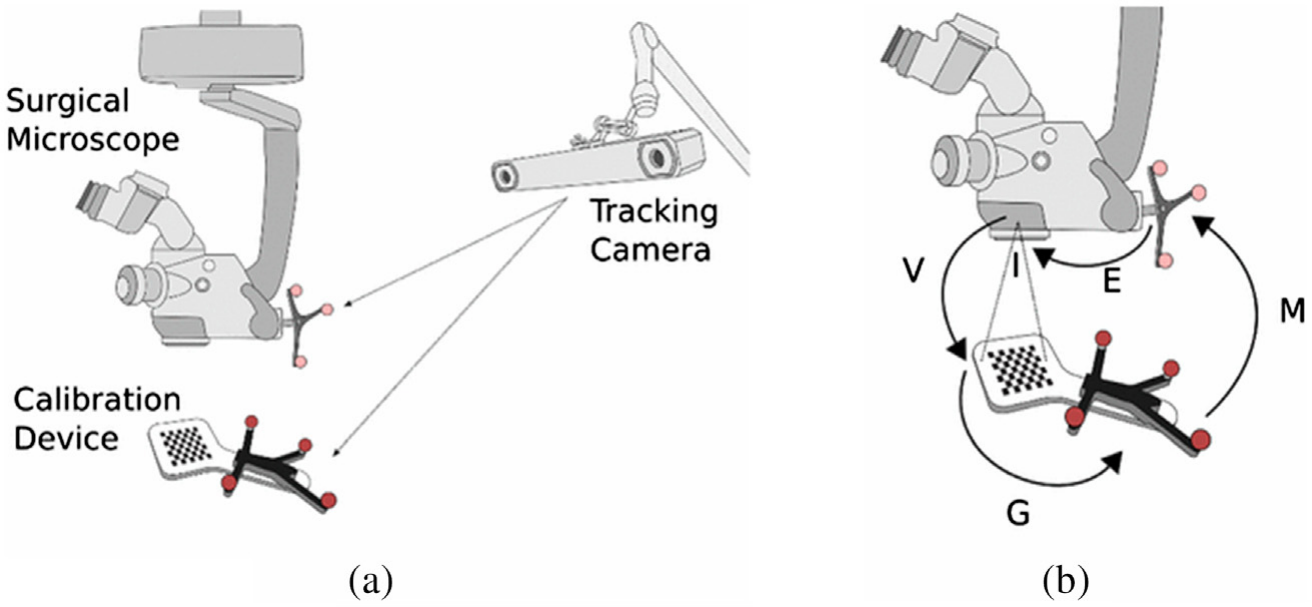
Illustration of the calibration method using checkerboard pattern: (a) system setup and (b) various transforms involved with the calibration method.^236^

The aim of calibration is to produce a projection matrix that will give the pixel position in the injected image of any 3D point relative to the frame of reference of the microscope.^63^ The process determines all camera parameters including the correction of optical errors generated by nonperfect lenses.^205^ Usually, several calibrations need to be done under different magnifications because the lens error values change with zooms,^199^^,^^205^^,^^235^ and the preoperative calibration process takes about 10 to 20 min.^57^ Edwards et al.^63^ used a calibration object that consisted of a number of localizer LEDs within the working region to enable the calculation of room coordinates. Mun et al.^221^ proposed a calibration algorithm that uses image intensity rather than fiducials and is adaptable for different lens distortion models. In some recent papers, the checkerboard patterns were used to calibrate the stereo camera integrated into the microscope head, as Fig. 11 shows.^218^,^236^

**Fig. 12:**
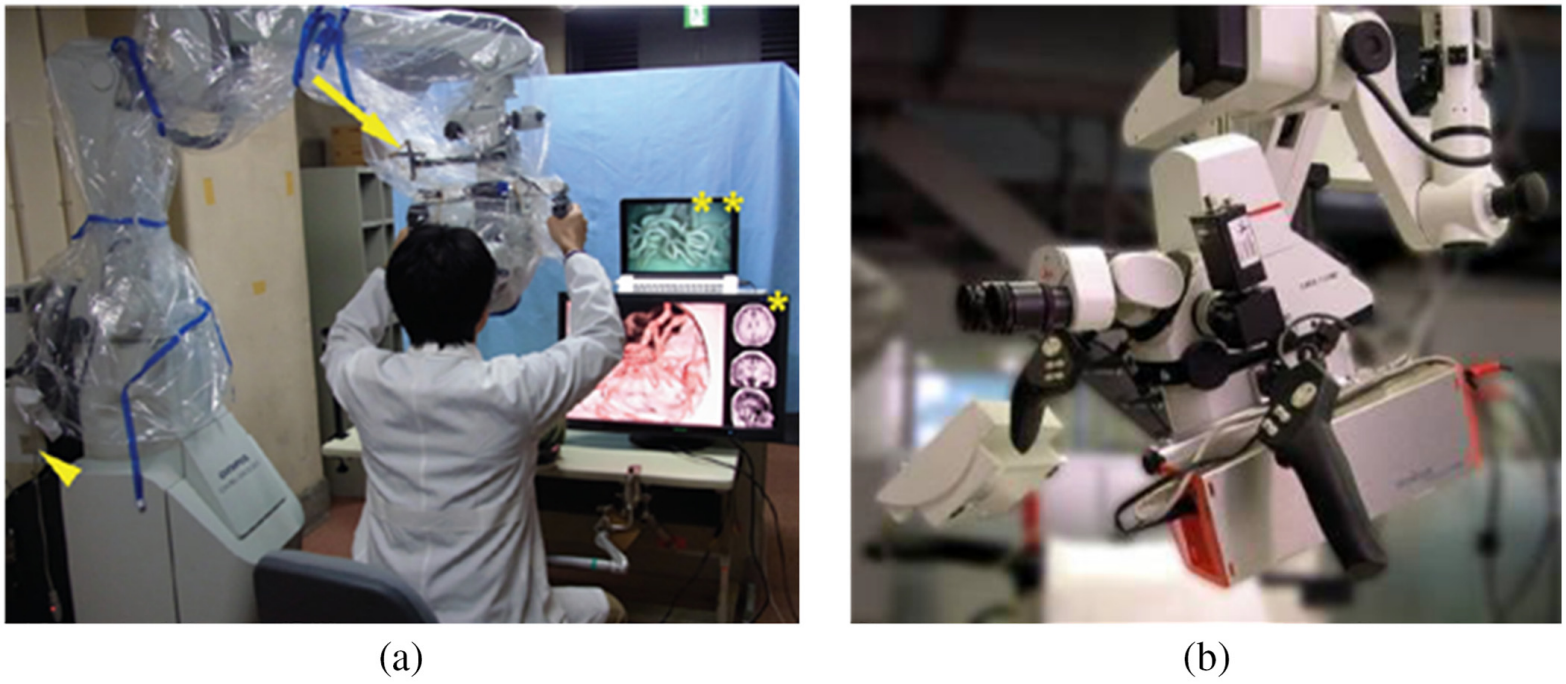
Tracking methods: (a) surgical microscope with optical tracker (yellow arrow)^235^ and (b) surgical microscope with integrated tracking camera.^199^

There are several tracking methods based on different devices. Friets et al.^202^ used an ultrasound range finding system, while Doyle^237^ used a magnetic field digitizer instead. However, optical tracking is the most popular way^63^^,^^199^^,^^206^^,^^210^^,^^232^^,^^233^^,^^235^^,^^238^ and has been in use in modern operating rooms for intraoperative navigation. It is facilitated by the widely available cameras, and it does not require any wire connection between the system and the tracked object. LEDs, IR-LEDs, or other optical trackers can be attached to both the microscope and the patient, as shown in Fig. 12(a), and a 3D optical localizer is utilized to track these light points.^63^^,^^206^^,^^235^ An acrylic dental stent with imaging and physical locators was developed and attached firmly to the patient’s upper teeth so the tracker was brought close to the volume of interest, and it allowed free movement of the patient’s head within the line-of-sight.^232^ Garcia Giraldez et al.^199^ integrated a 3D tracking camera onto the housing of a surgical microscope tube, and the camera tracked the movements of surgical tools and the patient, as shown in Fig. 12(b). Gard et al.^218^ used an image-based method to track a green-colored instrument tip under a surgical microscope for trajectory creation in tympanoplasty.

Registration is most commonly accomplished with fiducial markers,^57^^,^^207^^,^^235^ skin surface,^63^ and manual procedures^238^ as well as skull-implanted or dental-fixed fiducials.^239^^,^^240^ It is reported that fiducial markers or skin surface registration are the easiest, fastest, and most accurate ways with respect to manual registration,^210^ and they are less invasive and laborious than skull-implanted or dental-fixed fiducials.^210^ Overall, the registration errors for clinical AR range from 0.3 to 4.2 mm, mostly 2 to 3 mm.^199^^,^^206^^,^^207^^,^^212^^,^^218^^,^^232^^,^^235^^,^^241^

**Fig. 13:**
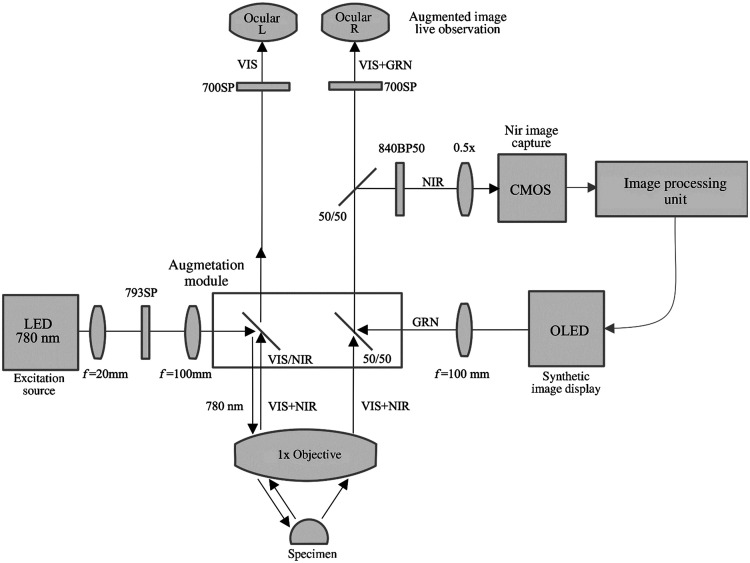
Schematic of the optical pathway in an augmented microscope, augmentation provided in the right ocular.^208^

**Fig. 14:**
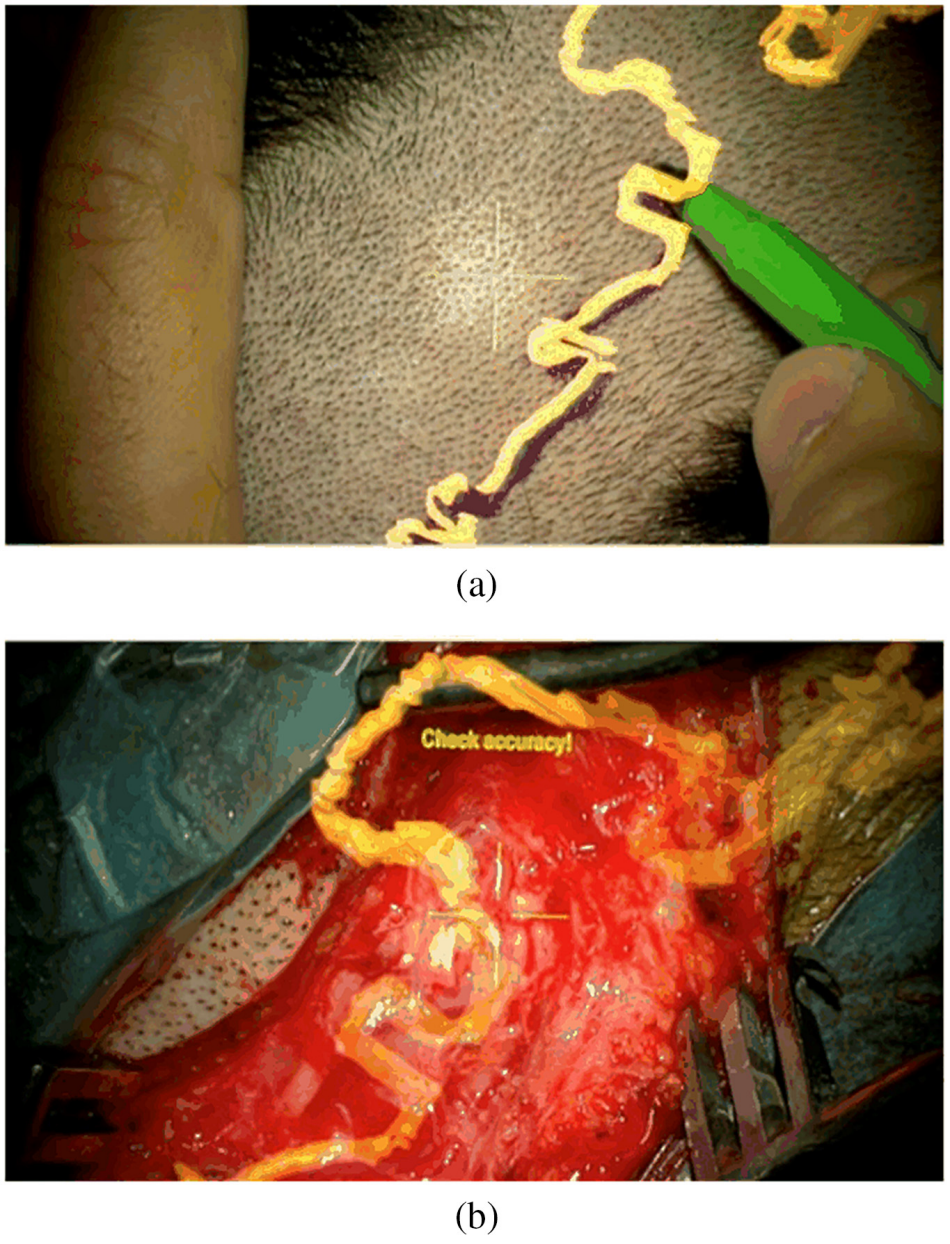
Image injection of the occipital artery allows detailed appreciation of its sinuous course. (a) This image overlay indicates where the incision should be performed and guides the dissection of the artery. (b) The accurate superposition of the real and virtual arteries.^230^

When combined with surgical microscopes, the display method of AR is usually image injection^57^^,^^208^ into either one microscope ocular or both. Displaying the bright-field image and the augmented image in two separate oculars may cause strain and fatigue since there is no cue for spatial coregistration of two types of images. Two embodiments of an augmented stereoscopic microscope using ICG fluorescence images were proposed by Romanowski et al.^242^ In the single-channel embodiment, the objective lens receives both NIR and visible bright-field images of the examined object simultaneously. The augmentation module (beam splitter between the objective lens and ocular lenses) separates the NIR image from the visible image, and then the NIR image is processed to generate a synthetic image. The synthetic image is directed into an eyepiece, while the visible image is shown in two eyepieces with different light intensity proportions. In the dual-channel embodiment, two cameras capture the NIR image and the visible bright-field image, respectively. After image processing, the ICG fluorescence image is overlaid onto the optical image of the same FOV, and the combined image can be directed into both eyepieces. Figure 13 shows the schematic of a single-channel design of an augmented surgical microscope for NIR fluorescence imaging. Furthermore, the three ways for 3D data representation^234^ are slice rendering (one slice of the volume data), surface rendering that shows the transition between structures, or volume rendering. Figure 14 shows the overlay of injected 2D and 3D models during bypass surgery.

#### 3.5.3 Challenges

Although AR has been a fast-developed technology, challenges still exist for it in clinical use, such as the registration error from compounded sources, the visual and tactile temporal asynchrony caused by system latency, the inattentional obstructed view by virtual component, and the time consumption for registration and verification.^210^ Injection of 3D images into microscope oculars alleviates the visual fatigue caused by the accommodation-divergence discrepancy, but the focal plane of the virtual image remains incongruent with that of the target.^212^ The impaired depth perception has been a problem for all AR systems.^243^^,^^244^ Binocular cues, partially offered by 3D stereoscopic displays, are not always sufficient for inferring the spatial relationships between objects in a 3D scene.^210^ Another challenge is the organ and tissue shift, such as the brain shift when the arachnoid is opened, which compromises the reliability of the virtual model of the AR system and navigation. Furthermore, in some surgeries such as arteriovenous malformation (AVM) resection, the role of AR is still limited because arterial feeders in most AVMs are not resolvable with current systems.^212^ The future direction of clinical AR involves the improvement of the AR system, the refinement of virtual models, and the adaptation of new imaging techniques such as magnetoencephalography and transcranial magnetic stimulation.^210^

### 3.6 Fluorescence Imaging

In neurosurgeries, it is difficult to distinguish different types of tissue, even with outstanding optics. For diseases such as malignant gliomas, MRI would suggest it as well-circumscribed tumors but fail to detect the wide margin of infiltrating tumor cells around. Moreover, the goal of removing all contrast-enhancing tumor on MRI is only achieved in <30% of cases.^245^^,^^246^ Fluorescence is an important method to aid visualization. Because different types of tissue absorb different amounts of fluorophores when exposed to the excitation light, the surgical field will appear in certain colors with an observation optical filter, and different tissues can be separated by human eyes due to the contrast provided by exogenous fluorescent dyes.

Before the fluorescence system was integrated into surgical microscopes, it had to be used individually. The surgical microscope had to be removed to place the fluorescence system, and the long-pass optical filter for fluorescence observation needed to be held manually.^247^ In this way, it was inevitable to interrupt the operation, and the use of intraoperative fluorescence became complicated. With the combination of intraoperative fluorescence imaging and surgical microscope, information of the surgical field can be provided in real time with accustomed magnification and no concern of organ shift.^248^ There are certain drawbacks of fluorescence imaging. For example, the limited dimension (2D information only) reduces the stereopsis of the FOV, and fluorescence can be obscured by overhanging tissue or blood.^248^ Nevertheless, the outstanding contrast and the lower cost compared with MRI or CT still make it popular in multiple surgical applications. Nowadays, with the various commercial modules available for integration with contemporary surgical microscopes, microscope-integrated intraoperative fluorescence has been widely used for tumor demarcation or video angiography.

#### 3.6.1 Fluorescence dyes

Although endogenous fluorescence (autofluorescence) of human tissue has attracted much attention in the biomedical imaging field, it requires UV-visible or NIR light source and probably needs to be combined with other techniques such as single-photon detection.^249^^,^^250^ On the other hand, various fluorescent dyes have been invented for different surgical applications and put into use. Most invented dyes generate visible fluorescence, so that with the help of visible exogenous fluorescence, surgeons are provided with a high contrast of visualization, and they are able to separate different tissues with human eyes. Besides the visible exogenous fluorescence, some dyes that emit fluorescence in the NIR wavelength range, such as the ICG, are also helpful during surgery.

**Fig. 15:**
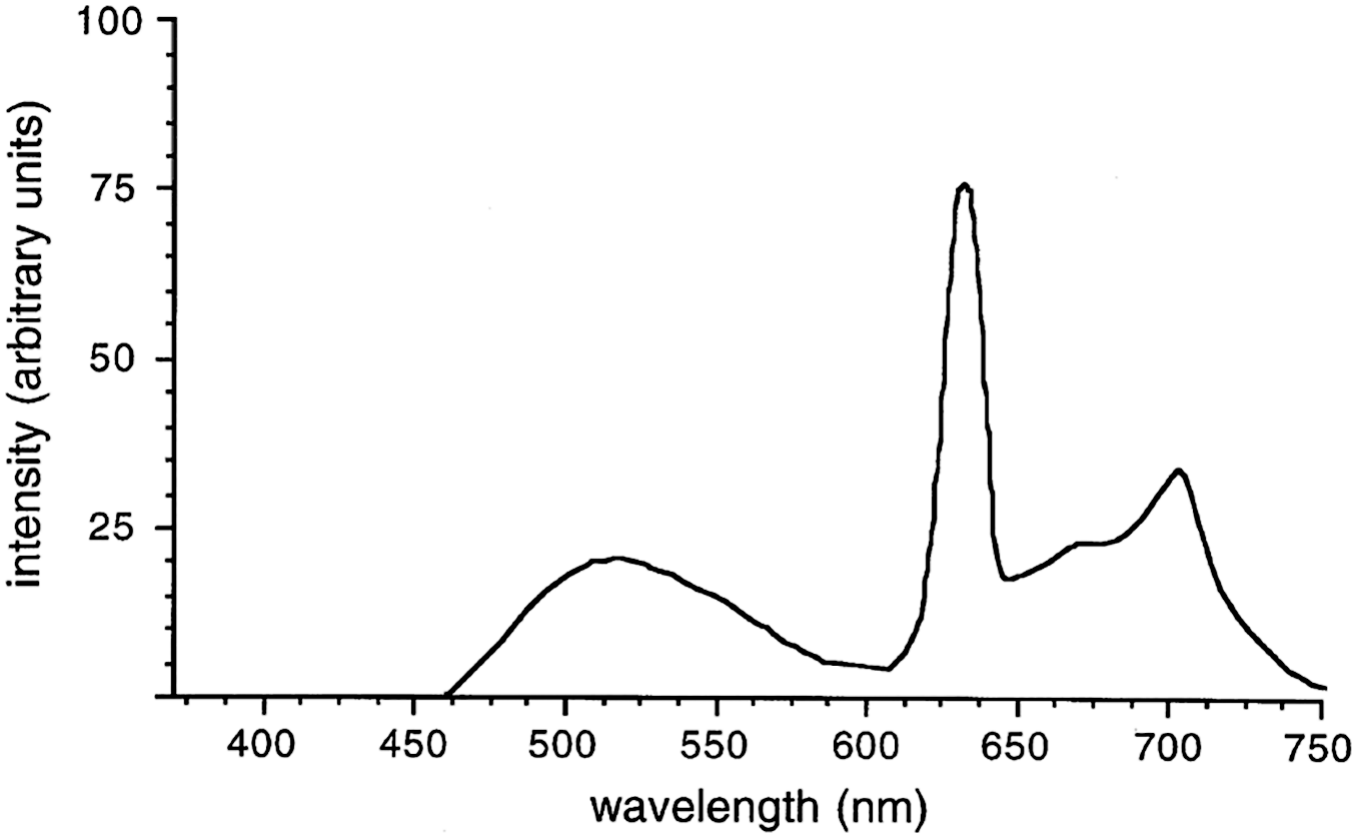
Fluorescence spectrum of malignant glioma superimposed with protoporphyrin IX spectrum.^247^

5-aminolevulinic acid (5-ALA) is a polar molecule to which the blood–brain barrier is virtually impermeable.^251^^,^^252^ It induces accumulation of protoporphyrin IX (PpIX), which is a kind of porphyrin, in the malignant tissues.^247^ The accumulated PpIX has a strong absorption band in the violet spectral range (380 to 420 nm) and emits red fluorescence (620 to 710 nm) with peaks at 635 and 704 nm in tissue.^31^^,^^253^^–^^255^ At the same time, there exists a broad band of endogenous fluorescence peaking at 520 nm. The excited fluorescence and endogenous fluorescence are superimposed, composing a spectrum, as Fig. 15 shows. Although the use of 5-ALA might cause minor side effects such as skin sensitization,^109^^,^^256^^–^^259^ it has been proved to be helpful for brain tumor resection. ^31^^,^^260^

Fluorescein sodium is an older compound that has been receiving new attention. It is a kind of fluorescent dye that accumulates in tumor tissues via disrupted blood–brain barrier.^27^^,^^248^ The fluorescence is based on passive permeability,^248^ and it is usually used in brain surgeries to distinguish the bright yellow-stained tumor and pinkish brain tissue. Although fluorescein can cause some adverse reactions, the use of sodium fluorescein (Na-Fl) in fluorescence-guided brain surgery has been reported as safe and effective.^27^^,^^109^^,^^261^

ICG is a cyanine dye that absorbs light ranging from 600 to 900 nm with an absorption peak above 800 nm.^262^^,^^263^ The fluorescence is also based on passive permeability,^248^ and it has a very wide spectrum in the NIR range. Because of the longer wavelength of fluorescence, ICG angiography is capable of achieving a deeper imaging depth than fluorescein angiography. Furthermore, it can be eliminated from the body by the liver within several minutes, thus it is safe to use. Nowadays, ICG is commonly used in cardiac, circulatory, hepatic, and ophthalmic conditions.^54^^,^^264^^–^^269^

#### 3.6.2 Integration of fluorescence imaging and microscope

**Fig. 16:**
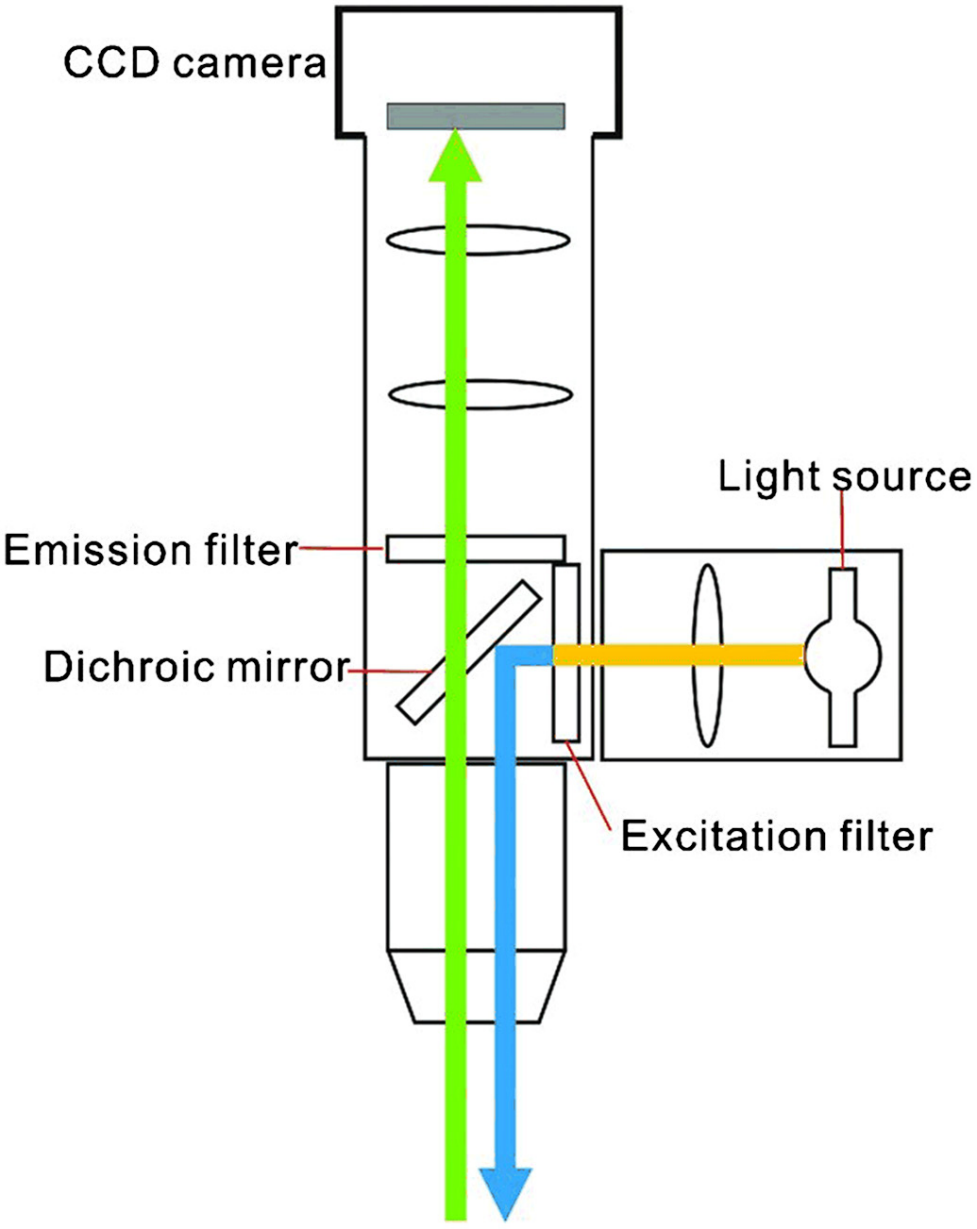
Schematic of a surgical microscope integrated with an intraoperative fluorescence imaging device.^270^

The integration of the intraoperative fluorescence system and surgical microscope frees surgeons’ hands and achieves an uninterrupted operating flow. The fluorescence system usually consists of an excitation light source, one short-pass filter for excitation light, one long-pass filter for fluorescence signal, and a camera to capture the fluorescence (for image-injection observation or recording), as Fig. 16 shows. The excitation filter is positioned in front of the light source, and the barrier filter is in the observation optical path. The fluorescence signal that passes the barrier filter can either go to the eyepieces and be observed by surgeons or be captured by a CCD camera mounted on the surgical microscope. One basic requirement is that surgeons can switch from white to excitation light (i.e., violet-blue) illumination directly under the microscope without interrupting the operation.^247^ Moreover, a certain level of excitation light irradiance is needed to ensure the adequate intensity of the fluorescence signal. It is said that irradiance of $40\;\mathrm{mW}/{\mathrm{cm}}^{2}$ is sufficient with an individual light source that is close to the human tissue.^247^ However, when the light source is integrated into the microscope, it has a distance of ∼250  mm to the surgical field, which reduces the excitation light irradiance that falls in the surgical field; the fluorescence observed by the main surgeon can also be weakened due to the share of view.

**Fig. 17:**
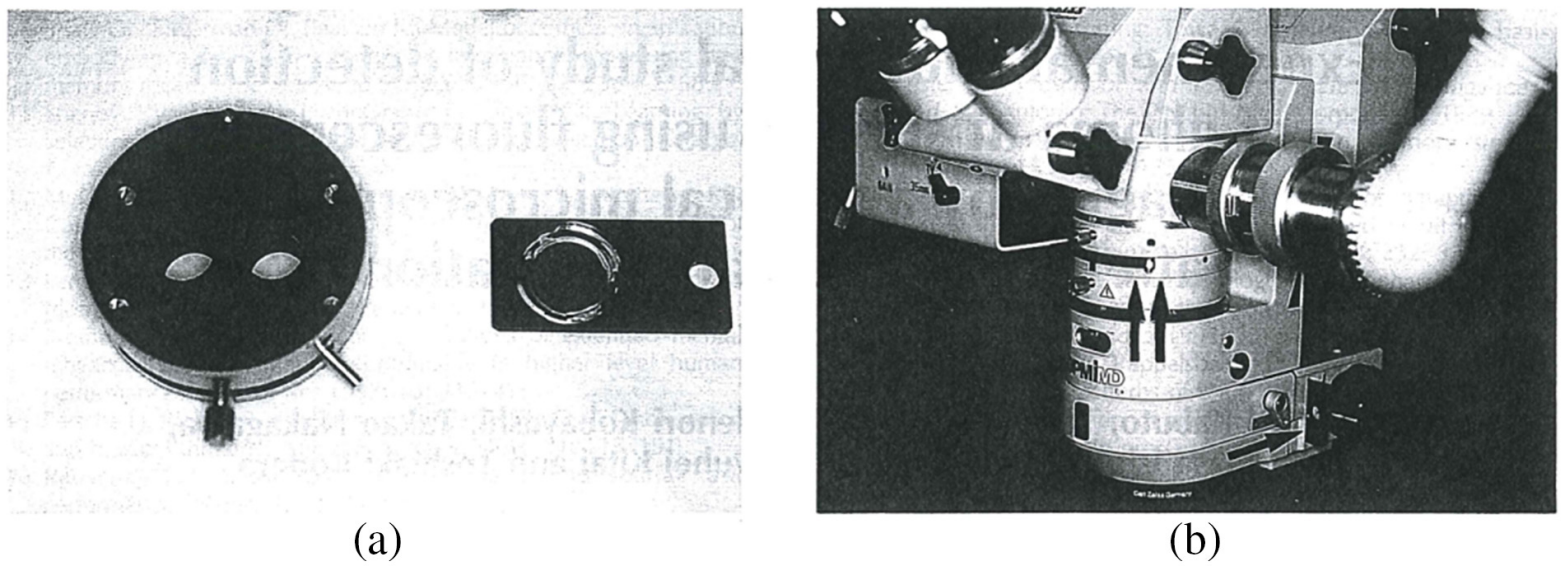
Two optical filters and their installation in the surgical microscope: (a) One (right) is a 380- to 500-nm wavelength band-pass filter (blue) for the excitation of fluorescein, and the other (left) is a 520-nm wavelength long-pass filter (yellow) for emission. (b) The surgical microscope can be easily modified for fluorescent microscopy simply by inserting the blue filter (arrow) in the pathway of the light from a xenon or halogen lamp and inserting the yellow filter (double arrows) in the pathway of the reflected and excited light.^6^

Kabuto et al.^6^ evaluated the effectiveness of fluorescence imaging by a surgical microscope with sodium fluorescein. A 380- to 500-nm band-pass filter and a 520-nm long-pass filter were applied for excitation and emission, respectively. The surgical microscope could be easily modified for fluorescent microscopy simply by inserting the blue filter [arrow in Fig. 17(b)] in the pathway of the light from a xenon or halogen lamp and inserting the yellow filter [double arrows in Fig. 17(b)] in the pathway of the reflected and excited lights. The optical filters and their installation are shown in Fig. 17.

**Fig. 18:**
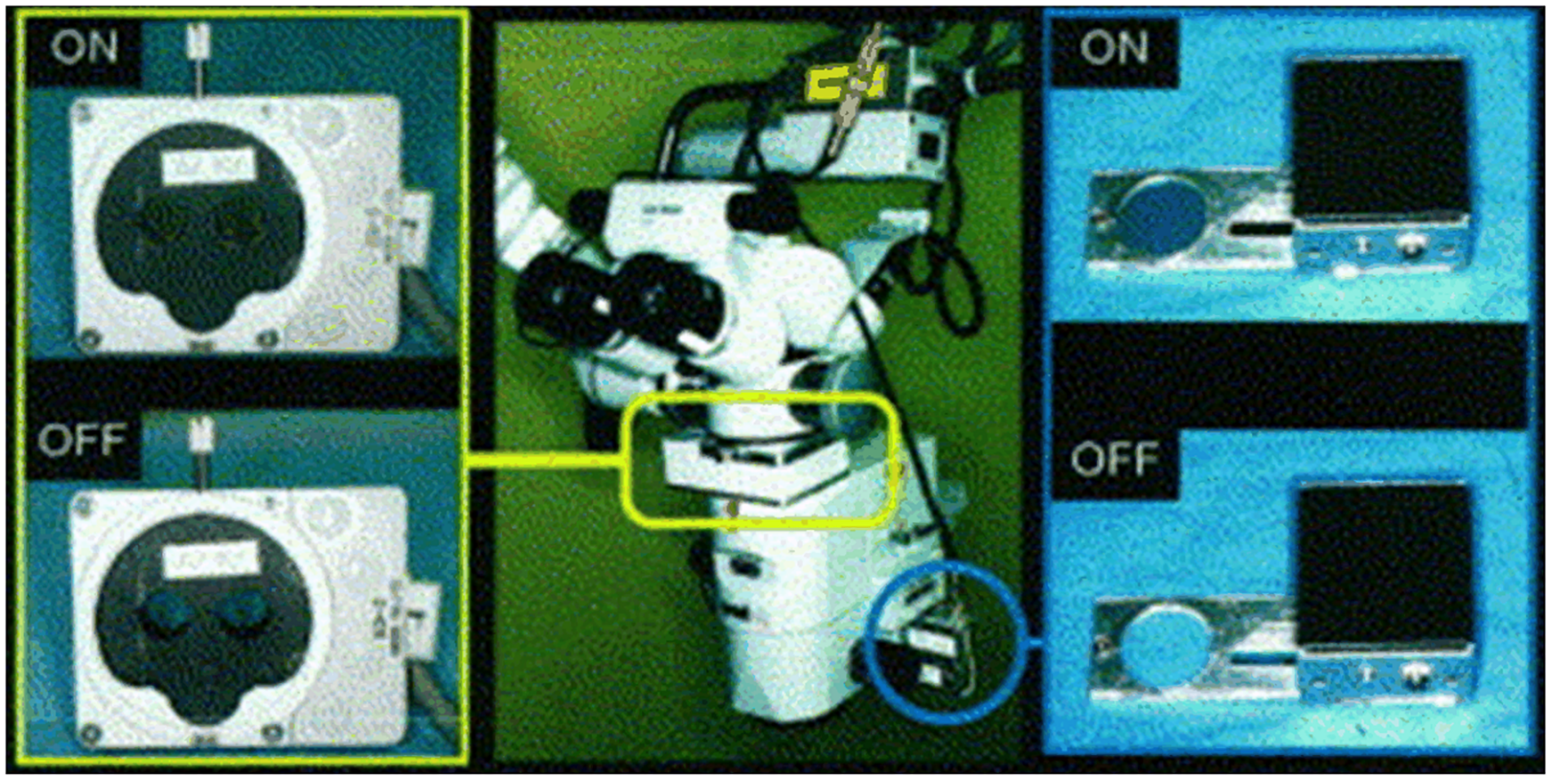
Photograph and diagram of fluorescein surgical microscope system.^271^

Kuroiwa et al.^271^^,^^272^ demonstrated a surgical microscope system, in which the microscope (Zeiss OPMI CS-NC or Zeiss OPMI MD) was equipped with a glass interference filter as excitation filter and a Kodak Wratten No. 12 filter as a barrier (observation) filter. The switch between white light and fluorescence modes was realized via a switching apparatus in which the filters can be inserted. The system, as Fig. 18 shows, was proved to be useful during malignant glioma surgeries.

Similarly, Stummer et al.^247^ demonstrated a Zeiss OPMI CS-NC surgical microscope with a modified varioscope for fluorescence observation. The violet-blue illumination irradiance was $40\;\mathrm{mW}/{\mathrm{cm}}^{2}$ at a working distance of 250 mm. An electromagnetic filter switcher was employed to introduce the long-pass observation filters into the two observer light paths of the stereo surgical microscope. Because beam splitters were used in both light paths for a second observer or camera recording, the main surgeon could obtain 50% of the fluorescence signal.

**Fig. 19:**
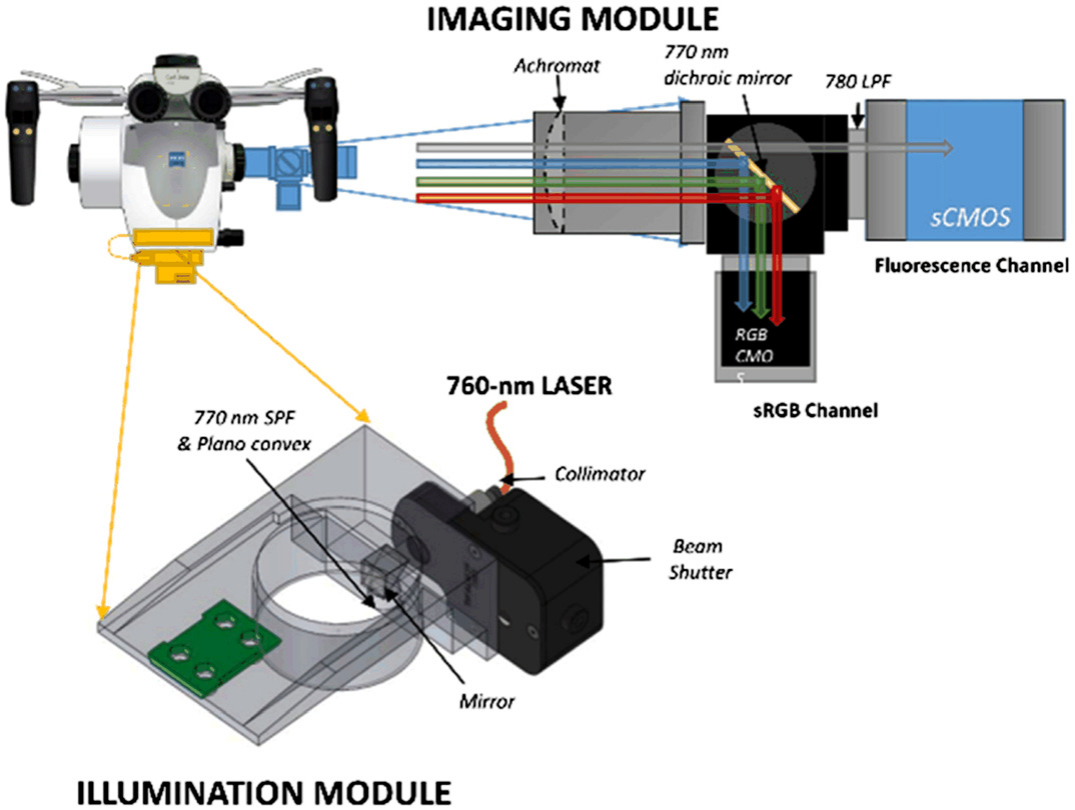
The detection module (blue) and the illumination model (yellow) are attached to the Zeiss Pentero OPMI head and can be used without affecting the standard operation of the microscope.^273^

Elliott et al.^273^ customized an illumination and imaging module enabling picomolar-sensitive NIR fluorescence imaging on a commercial Zeiss OPMI Pentero surgical microscope, as Fig. 19 shows. A continuous-wave 760-nm laser diode was employed as the excitation light source, and a dichroic mirror is used in combination with a long-pass filter to allow only light above 780 nm to enter the fluorescence-channel camera sensor. The customized system was tested with a new fluorescent agent named ABY-029 in live craniotomies in orthotropic tumor-bearing rats. Furthermore, Richter et al.^274^ combined fluorescence spectroscopy-based hand-held probe (HHF-probe) with fluorescence-guided resection surgical microscope (FGR-microscope) for tumor identification and resection. The surgical microscope adopted in this system was Leica M720 OH5 with the FL400 fluorescence module. The HHF-probe system included an excitation laser of 405 nm and a spectrometer operating in the range of 240 to 850 nm. The HHF-probe and FGR-microscope were used for the detection of 5-ALA-induced PpIX fluorescence in brain tissue. During surgery, the probe could be held very close to tissue. Clinical results show a better sensitivity of the probe and a better specificity of the microscope-integrated fluorescence module, and the combination of HHF-probe and FGR-microscope was proved to be beneficial especially in the tumor marginal zone.

**Fig. 20:**
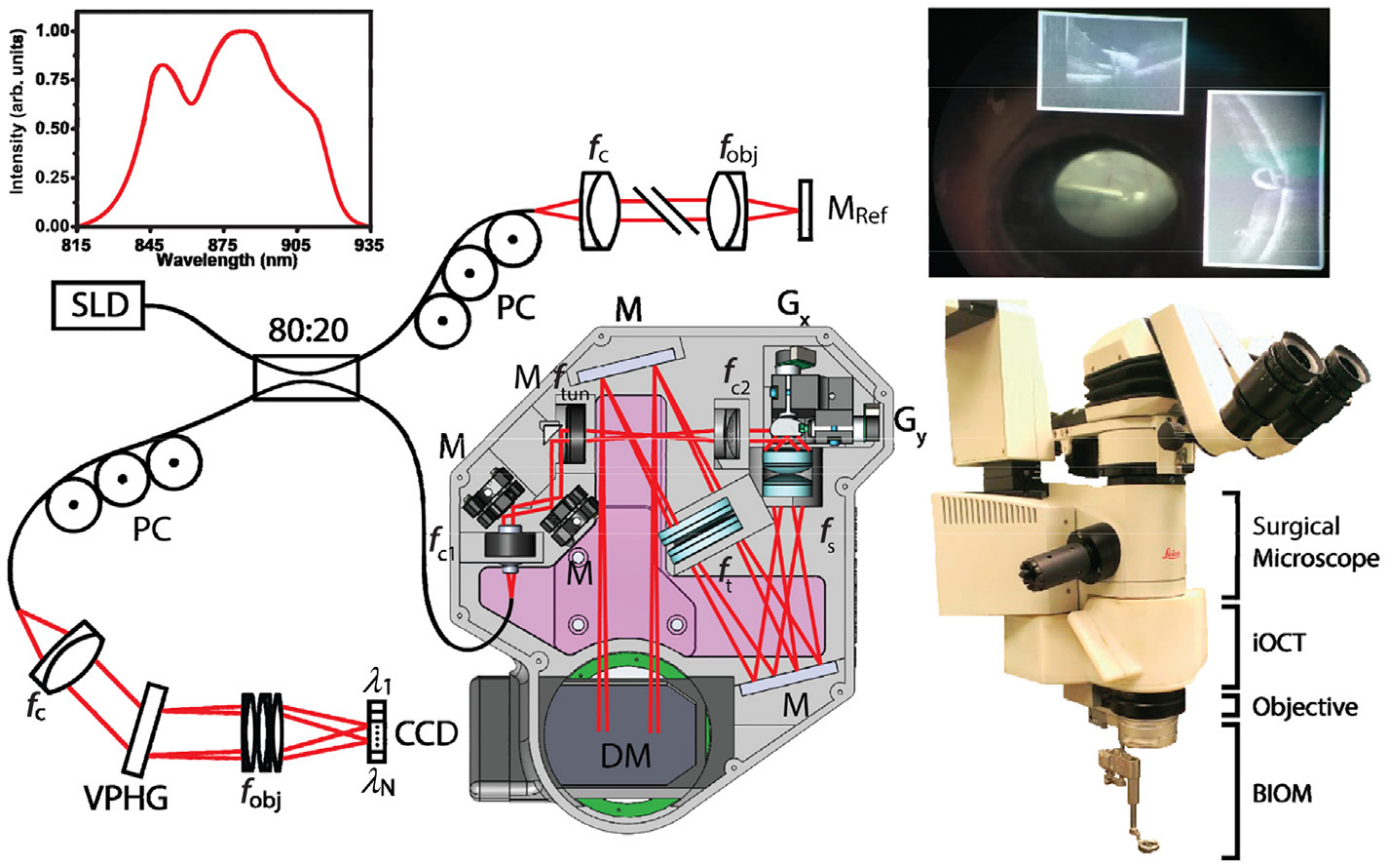
Microscope integration method of an iOCT system, the MIOCT beam is coupled into the microscope prior to the object lens but after the zoom.^23^

### 3.7 Intraoperative Optical Coherence Tomography

OCT is a noncontact and minimally invasive imaging modality that measures the scattered light from tissue.^275^^,^^276^ It is able to provide submillimeter-scale spatial resolution as well as subsurface information,^51^^,^^275^ which is vital for structural evaluation and instrument positioning during surgical procedures. Because OCT is based on optical communication technology, it is also more cost-effective.^275^ Compared with other tomographic imaging modalities, such as MRI and CT, OCT has much better intraoperative performance, such as higher resolution, no danger of ionizing radiation, and more compatibility with surgical instruments.^275^^,^^277^

OCT was first demonstrated for cross-sectional retinal imaging by Fujimoto’s team at Massachusetts Institute of Technology.^276^ Since the first perioperative study of OCT on epiretinal membranes,^278^ OCT has been proved to be capable of structural assessment, not only for the pathological structures but also surgically induced alternations.^279^^–^^285^ Nowadays, OCT is commonly used in ocular surgeries, since it can provide high-resolution images of tissue layers in both anterior and posterior segment that are poorly visualized with other conventional imaging modalities.^23^ Specifically, OCT has become critical for the diagnosis and management of ocular pathology^80^ and have been used widely in ophthalmic surgeries for the visualization of keratoplasty,^37^^,^^42^^,^^286^^–^^289^ epiretinal membranes,^90^^,^^290^ macular holes,^38^^,^^39^^,^^90^^,^^290^^,^^291^ retinal detachments,^38^^,^^80^^,^^292^ and vitreomacular traction.^38^^,^^39^^,^^291^^,^^293^ Moreover, it can also be used for breast cancer imaging,^277^ brain tumor imaging,^294^ nerve and microvascular biopsy,^279^ and otolaryngology,^51^^,^^295^^–^^298^ especially during tumor resection when noneffective evaluation of subsurface structure would cause improper margin assessment.^275^ Over the years, this technique has advanced with the improvement of laser sources, beam delivery instrument, and detection schemes,^299^ and has gradually become mature for clinical use. The integration of intraoperative OCT and surgical microscope not only enables the visualization of delicate structures but also keep the surgical workflow uninterrupted.^300^

#### 3.7.1 Intraoperative OCT and its applications

The principle of OCT is basically low-coherence interferometry. It typically uses NIR low-coherence (in other words, broad-band) light source, which can be superluminescent diodes, ultrashort pulsed lasers, or supercontinuum lasers.^277^^,^^301^ The light in the OCT system is split into two arms, namely the reference arm and the sample arm. After the light being reflected from tissue, the light beams from two arms form an interference pattern only if the difference of distances they have traveled is less than a coherence length. To take advantage of this interference, scanning is needed to obtain the location of the microstructure of the sample. According to the domain in which scanning is fulfilled, OCT is classified as time-domain OCT (TD-OCT), and frequency-domain OCT (FD-OCT) also known as spectral-domain OCT (SD-OCT). In TD-OCT, the path length of the reference arm changes with time, while in FD-OCT (SD-OCT) the broadband interference is acquired with a spectral variation using either a spectrally scanning light source or a dispersive detector. The reflective profile from a single axial depth scan is called A-scan. To achieve a 2D visualization of the FOV, a lateral combination of a series of A-scans is required for a cross-sectional tomography (B-scan).

Intraoperative OCT has three types of devices: handheld OCT (HHOCT), needle-based probes, and microscope-integrated OCT (MIOCT).^275^^,^^286^^,^^302^ When using HHOCT devices, surgeons have to remove the surgical microscope to place the HHOCT device over the patient’s eye. Needle-based probes overcome the above problem; however, it requires an assistant to hold.^275^ Microscope integration is a key advancement of OCT. Since this technology is now available integrated to ophthalmic surgical microscopes with an HD display to view intraoperative OCT images, it is possible for surgeons to give real-time feedback during the surgery. The integration achieves increased stability that handheld devices do not possess. Moreover, the X-Y-Z-foot-pedal control enabled by the microscope foot pedal facilitates rapid imaging with enhanced image reproducibility.^303^ Considering the fast scanning time of MIOCT, the minimal surgical pause is needed to switch between the surgical microscope and other OCT devices during the procedure, which greatly facilitates a smooth workflow. Furthermore, MIOCT would benefit procedures (1) with a reduced anterior chamber view, (2) where transparent or very thin structures are not readily visible with a surgical microscope, (3) that need to be monitored, and (4) where the intraoperative projection of preoperative imaging data is preferred.^300^

The early reported MIOCT systems have the limitation of an A-scan rate of <2  kHz,^296^^–^^298^^,^^304^^,^^305^ then it was improved to about 20 kHz.^306^^–^^308^ Although they achieved axial resolution as good as 7  μm^297^ and imaging depth of 2 mm,^298^ an acquisition time of 1 to 4 s was still required for a densely sampled volume. Moreover, they were incapable of real-time rendering of the volumetric data. Using a continuous high-resolution B-scan system, which consists of either two orthogonal B-scans or one single B-scan, live images could be generated of surgical procedures and maneuvers.^289^^,^^298^^,^^306^^,^^308^^–^^311^ With the outstanding computational speed of graphics processing units (GPU), live 3D OCT has been enabled for real-time volumetric data rendering.^312^^–^^314^ Four-dimensional MIOCT (4D MIOCT) system with dual-channel head-up display was reported since 2015 to be capable of live volumetric imaging through time at the speed of up to 10-μm-scale volumes per second,^78^^,^^315^^,^^316^ which is promising for ophthalmic surgery improvement.

#### 3.7.2 Integration of OCT with surgical microscope optics

The essentials of a microscope-integrated iOCT system are the microscope integration, a HUDs, and the scan control of OCT. The integration of the OCT module with a microscope is a challenging job, as it compromises the performance of either the OCT or the microscope to some extent.

In the first demonstrated MIOCT system, the optics of iOCT were separate from those of the microscope, and the MIOCT system was coupled to the surgical microscope with a dichroic mirror after the microscope objective lens, which resulted in a reduction of working distance.^275^^,^^317^ In the following designs, the MIOCT system (or part of the system) was coupled into the microscope using either a dichroic mirror or a beam splitter cube. Based on the position of the dichroic mirror (or beam splitter) in the microscope and the optics shared by MIOCT and microscope, the integration method of MIOCT can be divided into two types.

The first design couples the MIOCT system using a dichroic mirror located prior to the microscope zoom module.^296^^,^^318^^,^^319^ The optical beam of MIOCT is folded by the dichroic mirror into the path of one of the oculars, and then the merged beam goes through the zoom system and the main objective lens. Specifically, a real-time projection unit can be applied to project the generated OCT image to the other eyepiece.^319^ The MIOCT beam can be coupled from a camera port of the microscope. Therefore, the MIOCT system does not change the height and the working distance of the microscope. This design can achieve a lateral resolution of 23 to 47  μm and an FOV (lateral scan size) of 4 to 28 mm, with the microscope working distance in the range of 232 to 290 mm. The resolution and FOV are mainly limited by the diffraction, the shared magnification, and the working distance of the microscope.^296^

In the second design, the MIOCT beam is coupled into the microscope using a dichroic mirror prior to the objective lens but after the zoom module, as Fig. 20 shows.^23^^,^^108^^,^^188^^,^^309^^,^^310^^,^^318^^,^^320^^–^^327^ In this way, the MIOCT beam is decoupled from the microscope ocular path, and the objective lens is the only optic that is shared by the microscope and the MIOCT. In some designs, the OCT is integrated with the illumination module, so the OCT beam is coupled into the illuminating beam path.^108^^,^^320^^–^^322^ The other form of MIOCT is an independent unit that is assembled onto the microscope.^188^^,^^323^^,^^324^^,^^327^ Since the MIOCT does not utilize the microscope magnification anymore, independent control of beam waist diameter and beam waist position is usually added.^188^ Moreover, a telescope made of a reduction lens and a magnifier lens is applied after the microscope objective lens to magnify and provide wide-field image.^23^^,^^188^^,^^309^^,^^310^^,^^325^ Furthermore, using reflective elements and a tunable focus lens can help improve the transmission and the integration of MIOCT to the microscope.^23^^,^^310^ However, this approach increases the height of the microscope and the distance between the surgical field and microscope ocular, both of which could have an adverse effect on the microscope ergonomics.^275^

### 3.8 Hyperspectral Imaging

HSI, or multispectral imaging (MSI), is an emerging optical imaging technique, which is noncontact, noninvasive, nonionizing, and label-free.^328^ It acquires a 3D data cube, namely the hypercube, which contains a series of 2D grayscale images across a wide electromagnetic spectrum from UV to mid-infrared (MIR).^329^^,^^330^ The main difference between HSI and MSI is the number of spectral bands within the data cube. A data cube acquired by MSI usually contains 3 to 10 bands, while that acquired by HSI has tens or even hundreds of narrow spectral bands. HSI was originally developed for remote sensing, but it has been gaining attention in the medical imaging field. HSI utilizes the optical properties of human tissue such as reflectance and fluorescence, which are related to tissue microenvironment or biochemical constitutes.^330^ It can extend surgeons’ vision beyond the visible region,^331^ deliver near real-time biomarker information, and provide tissue pathophysiology through spectral characteristics.^330^ Therefore, it is promising for disease diagnosis and surgical guidance.^328^^,^^330^^,^^332^

One important application of HSI in the medical field is to extract the spectral signature of the recorded fluorescence signal to improve the accuracy of disease detection or differentiation and intraoperative metastatic diagnosis.^333^ On the one hand, HSI can be used to differentiate multiple distinct yet spectrally overlaid emissions of different dyes via spectral decomposition. On the other hand, it can be used to compute the quantitative estimates of fluorophore concentration through the processing of the recorded signal, so it can increase the system sensitivity to the fluorophore, enhance the contrast of fluorescence image, and improve the accuracy of disease detection or tissue differentiation.^333^^–^^335^

Meanwhile, various studies have revealed the potential of HSI as a dye-free imaging method to provide surgical guidance.^332^^,^^336^^–^^352^ The heterogeneous composition of biological tissue results in the spatial variations of optical properties, yielding distinct spectral signatures of tissue reflectance. With appropriate data processing methods, HSI alone is able to serve as an intraoperative tool to help surgeons visualize the surgical bed under the blood, facilitate residual tumor detection, monitor the tissue oxygen saturation, and enable the visualization of the anatomy of vasculature and organs.^330^ Without the requirement of any injection of contrast or dye, it can be used on-demand as many times as needed during surgery.

HSI can be classified into four types according to its acquisition mode, namely point-scanning, spectral scanning (wavelength-scanning), line-scanning (push-broom), and snapshot.^353^^,^^354^ The point-scanning mode is to scan the object point-by-point, which is slow and not commonly used anymore. Line-scanning mode is for the camera to scan the object along one spatial axis, and each time it acquires a complete spectrum for each pixel in a row of pixels. Line-scanning hyperspectral cameras have a good spatial and spectral resolution; however, it requires relative motion between the camera and the object (patient). The spectral-scanning method steps through the wavelength range and captures one grayscale image of the whole FOV at each step. Snapshot hyperspectral camera has filters integrated on the imaging sensor, and it is able to capture the spatial and spectral information simultaneously. The resolution of hyperspectral images depends on the imaging system, but generally, the spectral-scanning method achieves better spatial and spectral resolution than snapshot, while the snapshot method has a fast, video-rate acquisition speed. Both methods are applicable for adaptation with surgical microscopes.

Snapshot hyperspectral camera is small in size and light-weighted, and it can be easily adapted to the surgical microscope through a video adapter. Pichette et al.^45^ mounted a 16-band snapshot camera on the side port of a surgical microscope for intraoperative hemodynamic response assessment. The camera covered a 481- to 632-nm range with an image size of 256×512, and it was operated at 20 frame per second (fps) during the intraoperative imaging.

**Fig. 21:**
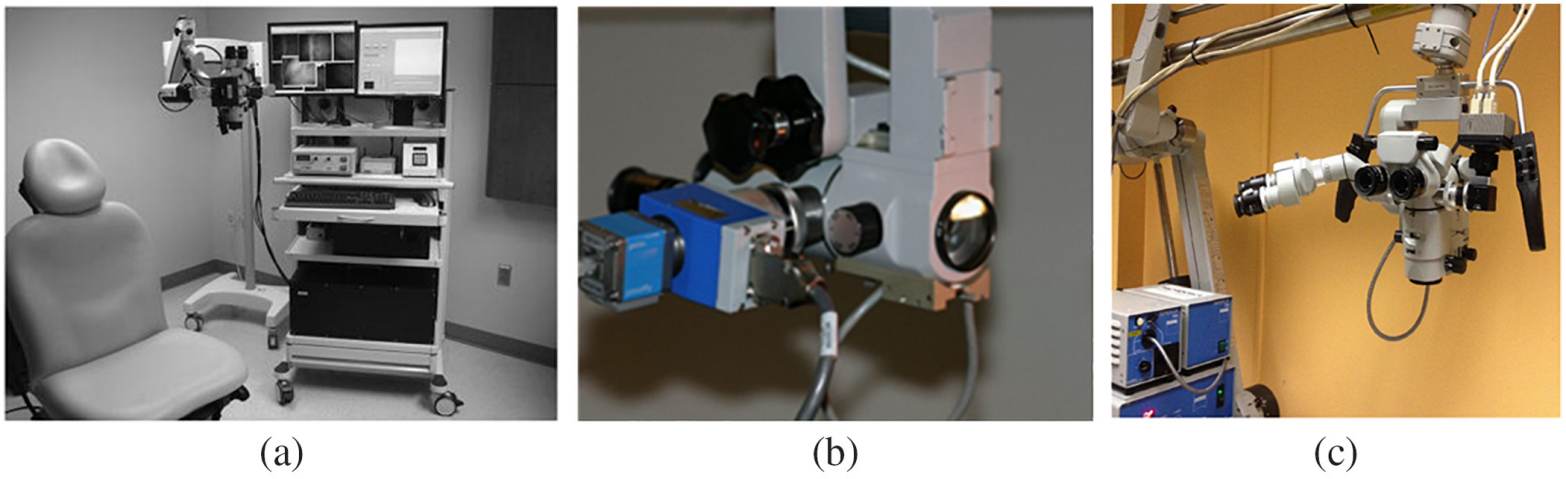
Surgical microscopes with HSI systems: (a) spectral-scanning HSI using filter wheel,^358^ (b) spectral-scanning HSI using tunable filter,^183^ and (c) snapshot hyperspectral camera.^45^

Spectral scanning HSI systems are made of a monochromatic sensor and a spectral-scanning component, which can be either electrical tunable filter^46^^,^^183^^,^^355^^,^^356^ or filter wheel.^43^^,^^48^^,^^357^^,^^358^ When a tunable filter is used, the imaging system (tunable filter and monochromatic sensor) is mounted on one of the microscope optical ports via a video adapter. Van Brakel et al.^183^ fitted a tunable filter and a high-resolution camera onto a surgical microscope for the observation of oral mucosa. The system could capture hypercube within 30 s with an image size of 1392×1024 and a wavelength range of 440 to 720 nm. Martin et al.^46^ developed a similar system for *in vivo* larynx imaging, which acquired images with 30 bands within 390 to 680 nm. For a filter wheel, it can be placed after the illuminator or in front of the imaging sensor. Roblyer et al.^358^ developed a spectral-scanning multispectral fluorescence surgical microscope using two four-filter wheels for oral neoplasia detection. The first illumination filter wheel was placed after the external mercury arc lamp to provide narrow-band illumination, and the second emitting filter wheel was mounted on the microscope optical port in front of the CCD camera to select autofluorescence signal. Wisotzky et al.^47^ employed a digital surgical microscope and a filter wheel with 19 band-pass filters for HSI within 400 to 700 nm. The wheel was located after the light source. Specific wavelength bands were selected by the filters, then the filtered light was transmitted to the microscope optics through a fiber guide to illuminate the surgical site. For comparison, the tunable filter is faster and easier for adaptation than a filter wheel, and it is capable of scanning more spectral bands. However, a filter wheel can provide higher resolution in some cases.^43^ Figure 21 shows three surgical microscopes adapted with different kinds of HSI systems.

### 3.9 Photoacoustic Microscopy

PAM, also named optoacoustic imaging, is a promising optical imaging method, which combines laser irradiation and acoustic wave to reveal subsurface structural, functional, and molecular information at microscale in real-time.^44^^,^^359^ The tissue being irradiated by nanosecond-range laser pulses absorbs the light and has a local temperature rise. The heat induces the thermal elastic expansion namely the photoacoustic effect and generates an acoustic wave, which is then received by an ultrasound detector.^359^^,^^360^ With some data processing methods, the distribution of light absorption can be obtained, which reflects the distribution of reflectors.^361^ During surgery, PAM can be helpful for the observation of fine structures under surface, such as microvascular mapping^362^ and ocular structure.^363^

**Fig. 22:**
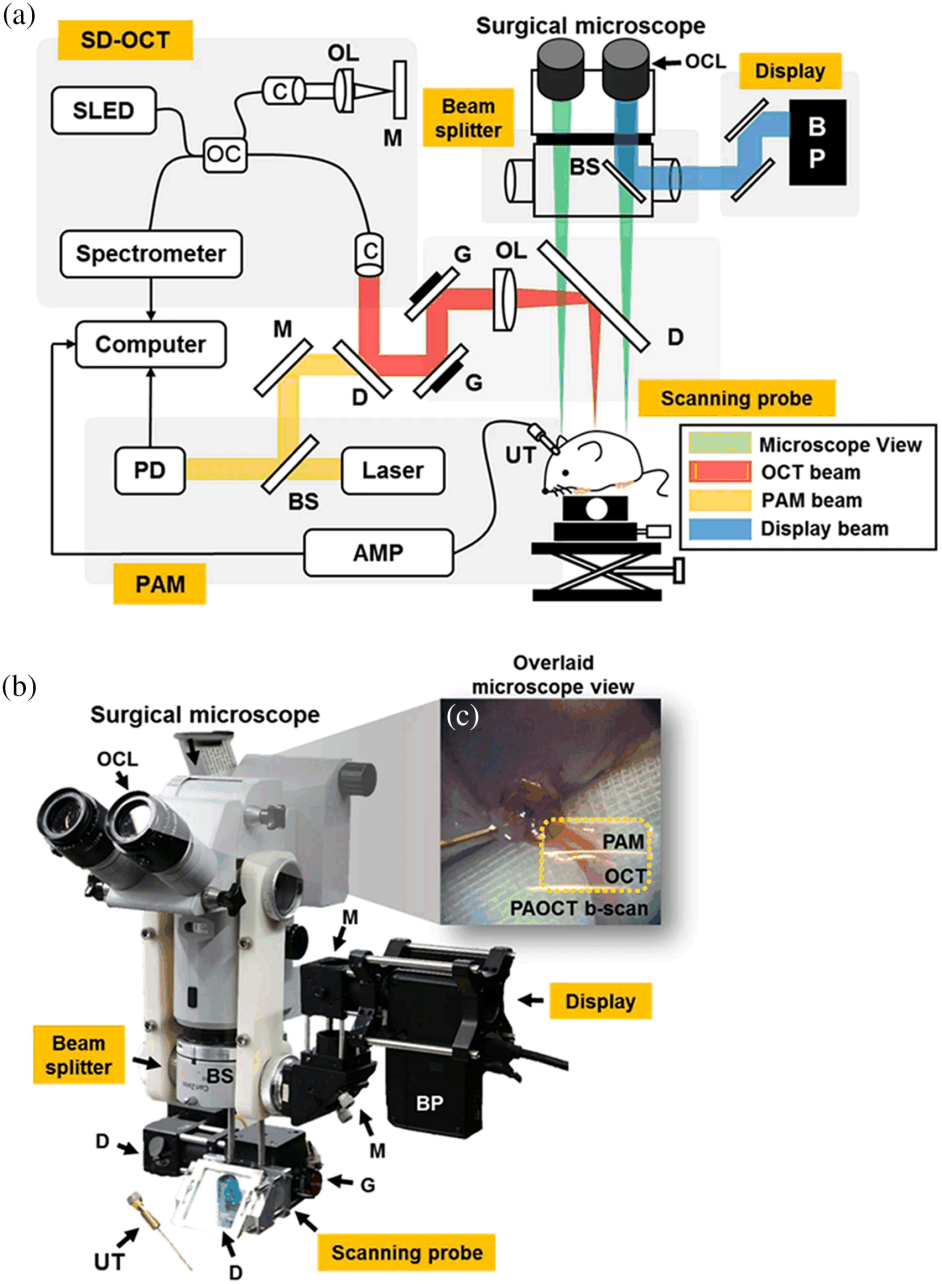
The NIR-VISPAOCT system and the FOV in ocular overlaid with PAM and OCT images. (a) schematic of NIR-VISPAOCT. (b) Photograph of the NIR-VISPAOCT probe. (c) Surgical microscope image overlaid with B-scan PAM and OCT images. SLED, superluminescent diode; C, collimator; M, mirror; G, galvanometer; OL, objective lens; OC, optical coupler; BS, beam splitter; AMP, amplifier; UT, ultrasound transducer; BP, beam projector; OCL, ocular lens; PD, photodiode; D, dichromic mirror; OCT, optical coherence tomography; and PAM, photoacoustic microscope.^366^

Han and Lee^44^^,^^364^^,^^365^ combined a commercial surgical microscope with a laser scanning PAM system for surgical guidance in *ex vivo* and *in vivo* studies. The PAM system shared the same optical path with the microscope, thus both the photoacoustic image and the white-light microscopic image were obtained simultaneously. The PAM scanning system was mounted under the microscope objective lens, consisting of two galvo scanners, an objective lens, and a beam splitter. The photoacoustic signal was detected by a needle-type transducer, which facilitates the application in the real surgery environment. In the first version, a laser of 532 nm was applied, and an axial resolution of 131  μm and a lateral resolution of 17  μm were achieved. The maximum penetration depth tested in a living mouse thigh was ∼900  μm. However, a portion of the green laser was projected to the oculars, which appears as a green spot in the microscopic images and disturbed the surgeon’s vision. Afterward, they adopted a 1064-nm pulsed laser and achieved an axial resolution of 65  μm and a lateral resolution of 41  μm. The invisible laser resolved the sight interrupt problem and laser safety concern for surgeons. Moreover, the 2D cross-sectional B-scan PAM images were projected onto the microscope view plan for AR. One year later, Lee et al.^366^ improved the aforementioned system by combining OCT and PAM altogether with a conventional surgical microscope. The presented near-infrared virtual intraoperative photoacoustic optical coherence tomography (NIR-VISPAOCT) system is shown in Fig. 22. The NIR-VISPAOCT system utilized both light absorption and scattering, and it was able to provide real-time information about tumor margins and tissue structure, as well as a magnified view of the region of interest. In an *in vivo* mouse melanoma resection surgery, the highly scattering skin layers were clearly shown in the OCT images, while the light-absorbing melanoma was very obvious in the PAM images. The surgical instruments were clearly seen in both images. The PAM system had an axial and lateral resolution of 63 and 35  μm, and those of the OCT system were 7.8 and 23  μm. By synchronizing both imaging systems, a real-time display with 23 fps was achieved for the B-scan PAOCT images.

### 3.10 Laser Speckle Contrast Imaging

LSCI, also named laser speckle imaging, laser speckle perfusion imaging, or laser speckle contrast analysis,^367^^–^^369^ is a technique that can be used for real-time assessment of perfusion. It is based on a phenomenon, where the backscattered light from a scattering medium that is illuminated by coherent light forms a random interference pattern, namely the speckle pattern. The movement of the scattering particles inside the medium causes fluctuations in the speckle pattern, which may result in blurring of speckle images.^370^^–^^372^ When illuminating tissue with a coherent laser and acquiring images of the tissue with adequate exposure time, the movement of red blood cells can cause fluctuation in speckle patterns, thus the blurring of the images can be related to the blood flow.^373^ This blurring of the recorded pattern is used to calculate the speckle contrast, which is useful for the quantitative analysis of blood flow. The speckle contrast of each pixel can be calculated in the spatial domain or the temporal domain. The spatial contrast of a pixel location is calculated using the intensity of all pixels in a defined neighborhood in one frame, while the temporal contrast is calculated using the same pixel in multiple frames in a time window.^374^ LSCI is noninvasive, relatively simple, and cost-effective.^370^ Most importantly, compared with the single-point laser Doppler flowmetry and the laser Doppler imaging that is relatively slow, LSCI is fast and full-field, providing a 2D perfusion map without scanning.^375^ Therefore, it can be very useful for intraoperative and postoperative visualization of tissue perfusion and measurement of blood flow velocity for retina,^376^ brain,^368^^,^^377^ skin microvasculature,^375^ liver,^378^ large intestine,^379^^,^^380^ and oral cavity.^381^^,^^382^

Richards et al.^383^ combined LSCI with a surgical microscope by attaching a laser adapter to the bottom of the microscope head and connecting a camera to one of the side ports. The laser adapter contained a laser diode of 660-nm wavelength and a curved mirror to diffuse the laser light. The system could achieve an FOV of ∼2×1.5  cm with the maximum zoom of the microscope and a frame rate of 100 fps with an exposure time of 5 ms. Moreover, the laser adapter and the camera, which were attached to the microscope preoperatively, did not interfere with sterile draping or normal use of the microscope. In this study, the system was used to image the cortical area of 10 patients. LSCI images were recorded for 10 to 15 min for each patient. The patient’s electrocardiogram (ECG) waveform, as well as camera exposure signal, were recorded simultaneously during the image acquisition. With a retrospective motion correction by ECG filtering and image registration, the system achieved satisfying accuracy and sensitivity and could facilitate clear visualization of small cerebral blood flow (CBF) changes.

**Fig. 23:**
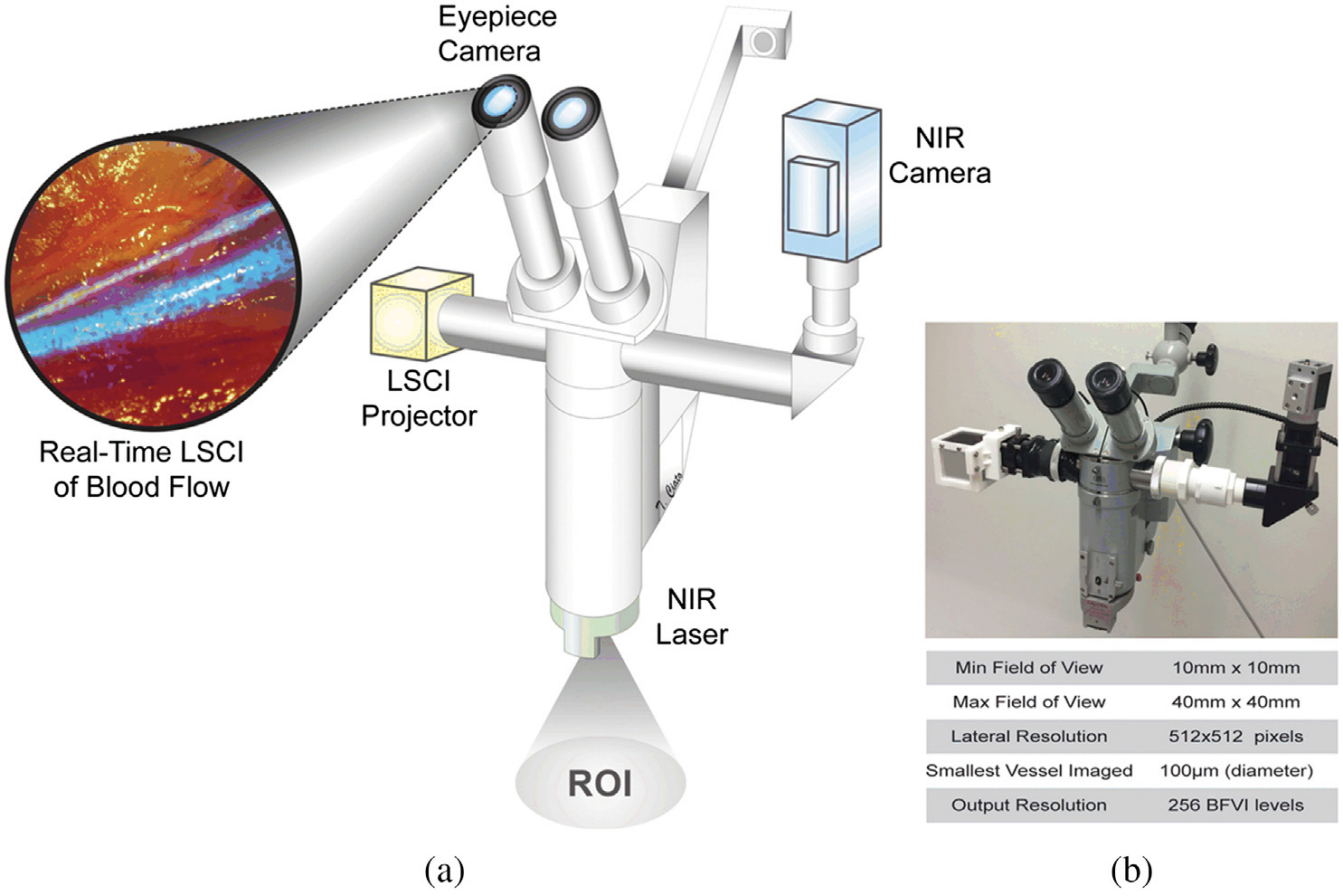
SurgeON system schematic and specifications. (a) As shown in the schematic, the SurgeON System comprises a surgical microscope modified to include the following key components: an NIR laser source irradiates the target ROI images which are then captured by the NIR camera. This camera is connected to a computer via MATLAB environment where these acquired laser speckle data are processed in real-time and a video-feed of the resulting blood flow information is transferred to the LSCI projector to be seen by the operator through the microscope eyepiece. (b) The SurgeON System has imaging specifications that are suitable for neurosurgery.^384^

Mangraviti et al.^384^ developed the SurgeON system, which could continuously monitor the CBF and display the high-resolution LSCI images directly onto the eyepiece of the surgical microscope, as shown in Fig. 23. The components that were attached to the surgical microscope include a laser adapter with a laser source of 830-nm wavelength, a custom NIR camera module, and an LSCI projection module. The system had a lateral resolution of 512×512 and a circular FOV with a diameter of 10 to 40 mm, which is determined by the microscope zoom. The LSCI images captured by the NIR camera were processed in a GPU-based computer in real-time to estimate the blood flow velocity index (BFVI) of each pixel, and then the generated BFVI images were projected onto the microscope eyepiece. The frame rate of the system was continuously >60  Hz. The results of a series of preclinical studies show that the SurgeON system could enable the detection of acute perfusion changes as well as temporal response patterns and degrees of flow changes in various microvascular settings. Furthermore, by comparing LSCI with ICG videoangiography, this study demonstrated that the dye-free LSCI could not only provide more information such as the CBF variation but also guide the surgery in real-time.

## 4 Clinical Applications

A surgical microscope is able to provide a clear, bright, and magnified view of the surgical site, and its integrated or adapted advanced technologies can greatly facilitate surgical navigation. Moreover, the use of a surgical microscope could potentially reduce trauma^26^^,^^385^ and hospitalization time.^14^ Therefore, surgical microscopes have been widely used in various surgical fields, such as brain tumor resection,^31^^,^^260^ thyroidectomy,^64^ retinal detachment,^38^ root canal therapy,^91^ and lymphaticovenular anastomosis (LVA).^86^ In the following section, we will discuss the applications of the surgical microscope in neuro and spine surgery, ENT surgery, ophthalmic surgery, dentistry, and plastic surgery.

### 4.1 Neurosurgery and Spine Surgery

Visualization of fine texture in neuro and spine surgery is crucial. A bright, clear, and magnified view of the surgical site is essential especially when surgeons need to deal with the small vessels, the nerves proximal to the brain and spine cord, and the narrow cavities.^9^^,^^10^ The microscope can be applied in many neuro and spine surgeries, such as neuromuscular neurotization,^386^ parotidectomy,^387^ and dura surgery.^388^ The high magnification provided by the microscope, sometimes combined with intraoperative imaging methods, can enable total resection of tumor,^389^ safe and precise manipulate of vessels,^390^ and minimally invasive operation of the spinal cord.^25^ AR and microscope-integrated fluorescence modules are widely adopted in neuro and spine surgeries to aid visualization and provide the intraoperative diagnosis. For example, the AR navigation system could facilitate complicated spine keyhole surgeries,^50^ sodium fluorescein-guidance can improve the total removal of brain tumor,^109^ and the ICG videoangiography is able to assist visualization of small vessels during aneurysm surgery.^391^ In this section, we will discuss how surgical microscopes are applied in neuro and spine surgeries, mainly the neuro-oncology and neurovascular microsurgeries.

#### 4.1.1 Neuro-oncology

Surgical microscopes can aid the visualization of tumor margin during resection surgery and help achieve total resection of the tumor.^27^^,^^109^^,^^389^ It has been widely used for the surgical treatment of primary and metastatic brain tumors, as well as spinal tumors.

##### Brain tumor resection

Surgical microscope is essential for the resection of brain tumors, such as glioma,^52^^,^^260^^,^^272^ meningioma,^31^ intracranial aneurysm (IA),^57^^,^^391^^,^^392^ supratentorial tumor, pineal tumor, infratentorial tumor,^261^ metastasis tumor,^393^ and pituitary tumor.^394^ Compared with gross-total resection, microscopic total resection has better local control of tumor, and potentially decrease the risk of local recurrence.^395^ Because of the macroscopically unclear tumor margin, imaging techniques are usually required to locate the tumor, such as intraoperative 5-ALA fluorescence or AR with MRI images. A neuronavigation system combined with a surgical microscope can also offer great help. A surgical microscope in brain tumor resection not only offers clear and bright visualization as a routinely used instrument but also works as a framework that facilitates imaging-guided resections.

**Fig. 24:**
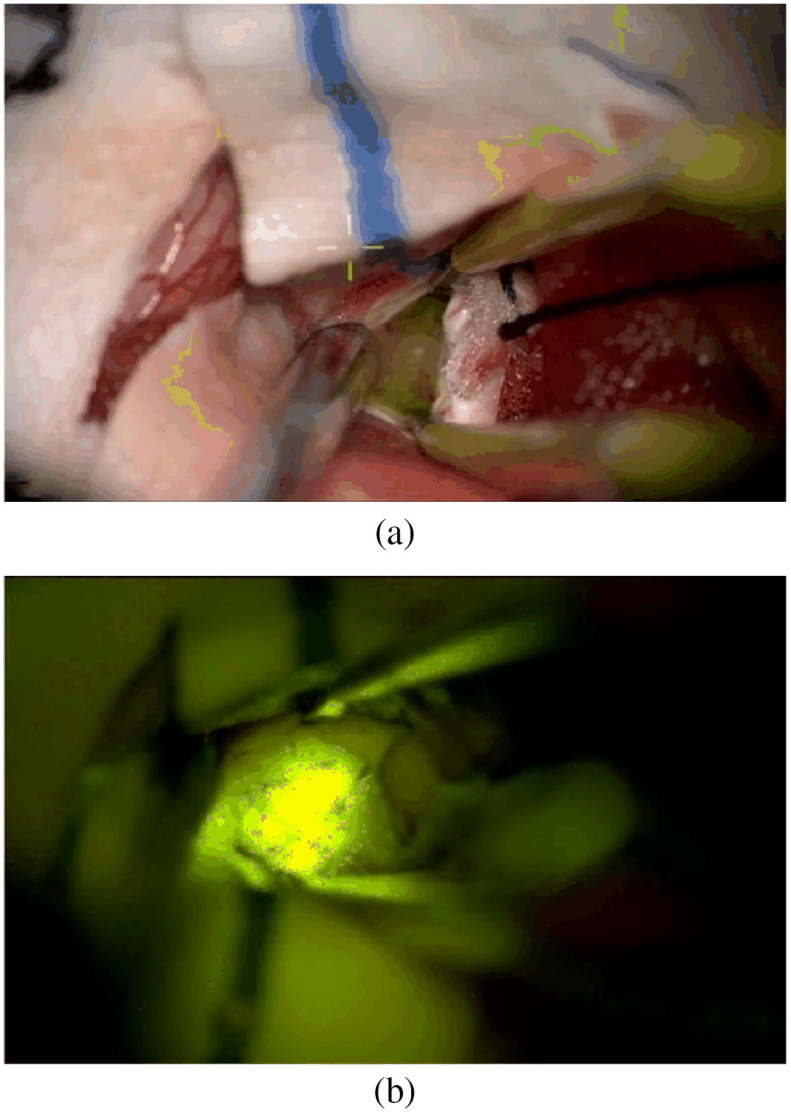
Intraoperative view of fluorescein-guided pediatric brain tumor surgery: (a) under microscope white light illumination and (b) fluorescein enhancement under YELLOW 560 filter.^29^

Kabuto et al.^6^ produced two filters for the excitation and emission of fluorescein and fitted them with a Zeiss surgical microscope for the detection of glioma during resection. In the *in vivo* study on rats, the tumors were stained with clear yellowish-green color, the adjacent brain was weakly stained later than the tumor, and the normal brain was not stained. In the clinical study of patients, the glioblastomas that were enhanced in MR images were stained with a yellowish-green color, the peripheral area where invaded glioma cells existed was stained later and more weakly, and the normal brain was not stained at all. Kuroiwa et al.^271^ developed a fluorescein surgical microscope with a BP450-490 filter and a Kodak Wratten No. 12 filter for malignant glioma resection. They tested the system on 10 malignant glioma patients. The results show that the system could enable adequate removal of the tumor within a short time. Later in 2016, they performed surgery on 30 cases of malignant glioma resection with their fluorescein surgical microscope,^272^ and the operative finding coincided with historical ones, which proved the fluorescein surgical microscope a useful aid in glioma surgery. Similarly, Stummer et al.^247^ employed a Zeiss OPMI CS-NC surgical microscope for 5-ALA-induced PpIX fluorescence observation in over 50 glioma resections. The xenon light source had strong light intensity, which yielded high tumor fluorescence. The electromagnetic filter switcher enabled the easy introduction of the emission filter without disturbing the surgical flow. Vital tumor regions and tumor-normal boarder was easily recognized with the help of microscope-mounted fluorescence. Acerbi et al.^396^ performed surgeries for grade IV gliomas in 15 patients using the Zeiss OPMI PENTERO microscope with BLUE 400 or YELLOW 560 for visualization. 5-ALA-guided resection of gliomas using BLUE 400, which is a fluorescence kit of Zeiss with a maximum excitation of 494 nm and maximum emission at 521 nm, was performed in the first several cases. Then, another fluorescence kit YELLOW 560, which excites in 460 to 500 nm and observes in 540 to 690 nm, was employed in the rest of the patients. Both kits allowed good visualization of tumor, but YELLOW 560 offered improved visualization of the nonfluorescent normal tissue in the fluorescence mode, as well as a better quality of video reproduction. The contrast-enhanced tumor was completely removed in 75% of the patients, and the remaining patients had a mean tumor resection of 90.5%. Twenty biopsies at the tumor-normal margin in five patients showed a high sensitivity (91%) and specificity (100%) of fluorescein tumor identification. Richter et al.^274^ combined a Leica M720 OH5 surgical microscope with a handheld fluorescence-spectroscopy probe (HHF-probe), which detected the 5-ALA-PpIX fluorescence for tumor identification and FGR. The system was evaluated in 18 operations on 16 patients with suspected high-grade gliomas, and the HHF-probe was compared with FL400, which is a commercial fluorescence module of Leica. Results showed better sensitivity of the probe than a normal FGR surgical microscope, especially with low fluorescence ratios, which is beneficial for complete tumor removal. White et al.^397^ developed a microscopic navigation system combined with the Viewsite Brain Access System (VBAS) technique for deep brain lesions and employed this system in high-grade glioma surgery. After craniotomy, the microscope with the navigation system was registered to the position and placed over the exposed surgical site. The crosshairs seen through microscope eyepieces were used to target the deep lesion, and the VBAS was carefully inserted into the brain for a smooth deep tumor dissection with minimal associated morbidity. Göker and Kırış^29^ evaluated the safety and feasibility of sodium fluorescein-guided surgery with Zeiss OPMI PENTERO 900 surgical microscope and YELLOW 560 filter in pediatric brain tumor surgery. The 23 pediatric patients had different intracranial pathologies, including seven gliomas, six supratentorial tumors, four intraventricular tumors, two infratentorial tumors, two pineal tumors, one clivus tumor, and one tumor with supra and infratentorial extensions in the current series. The fluorescence was helpful for tumor identification in 20 cases, and total resection was achieved in 11 cases. Figure 24 shows the tumor differentiation with YELLOW 560.

**Fig. 25:**
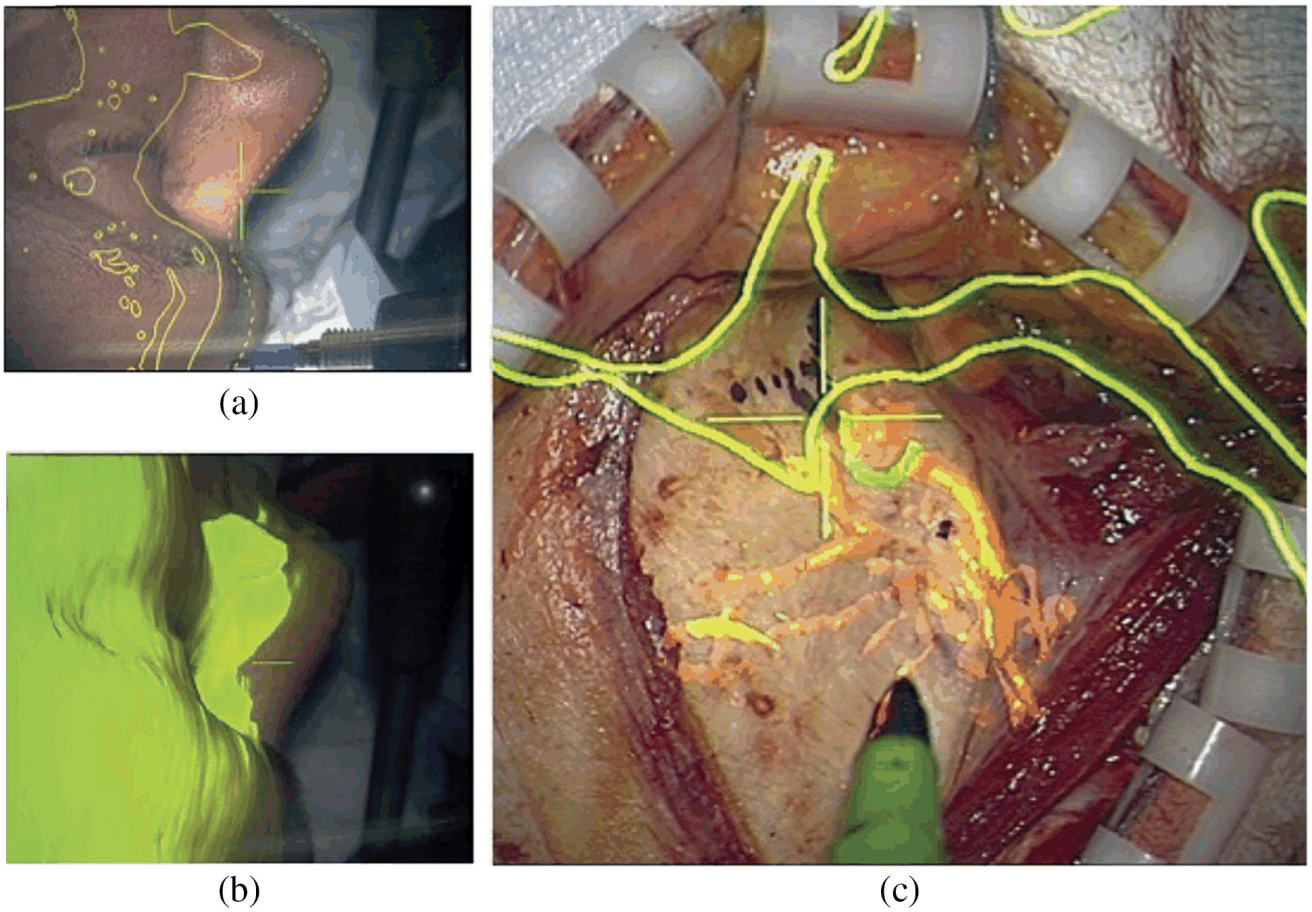
AR aneurysm surgery with image injection of the patient’s head: (a) image injection of the patient’s head in 2D, (b) image injection of the patient’s head in 3D, and (c) image injection of a right middle cerebral artery bifurcation aneurysm in 3D and of the underlying bony sphenoid ridge in 2D.^57^

**Fig. 26:**
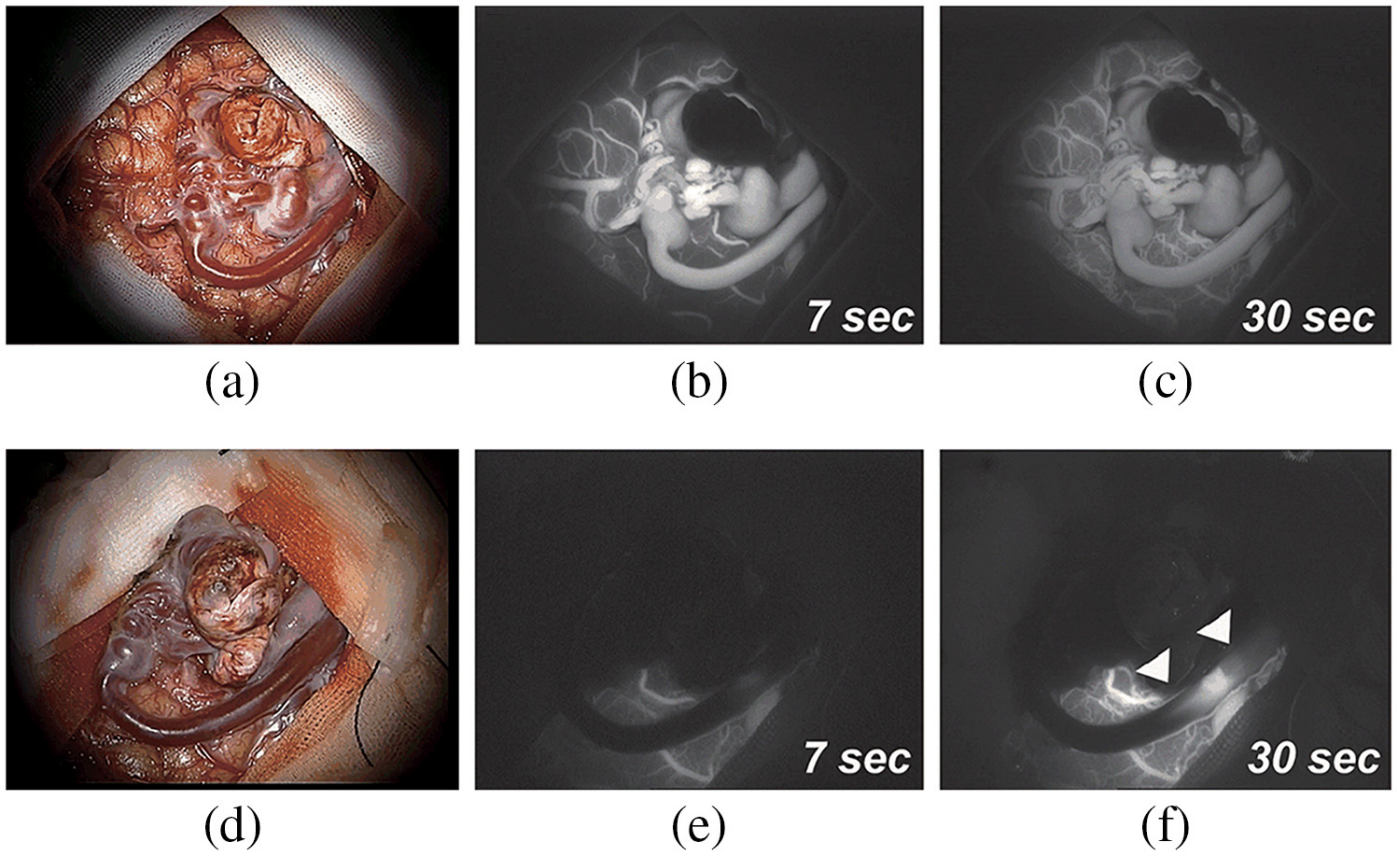
Intraoperative ICG videoangiography during arteriovenous malformation surgery. (a, d) Surgical view and (b, c, e, f) ICG videoangiography at the time ofoperation. Intraoperative ICG videoangiography disclosed the arteriovenous malformation clearly (b, 7 seconds after cerebral circulation was visualized by ICG; c, 30 seconds after that). After detection of the nidus, ICG videoangiography indicated no filling of the nidus and to-and-fro filling of the drainer (e, 7 seconds after cerebral circulation was visualized by ICG; f, 30 seconds after that; arrows indicate the drainer).

**Fig. 27:**
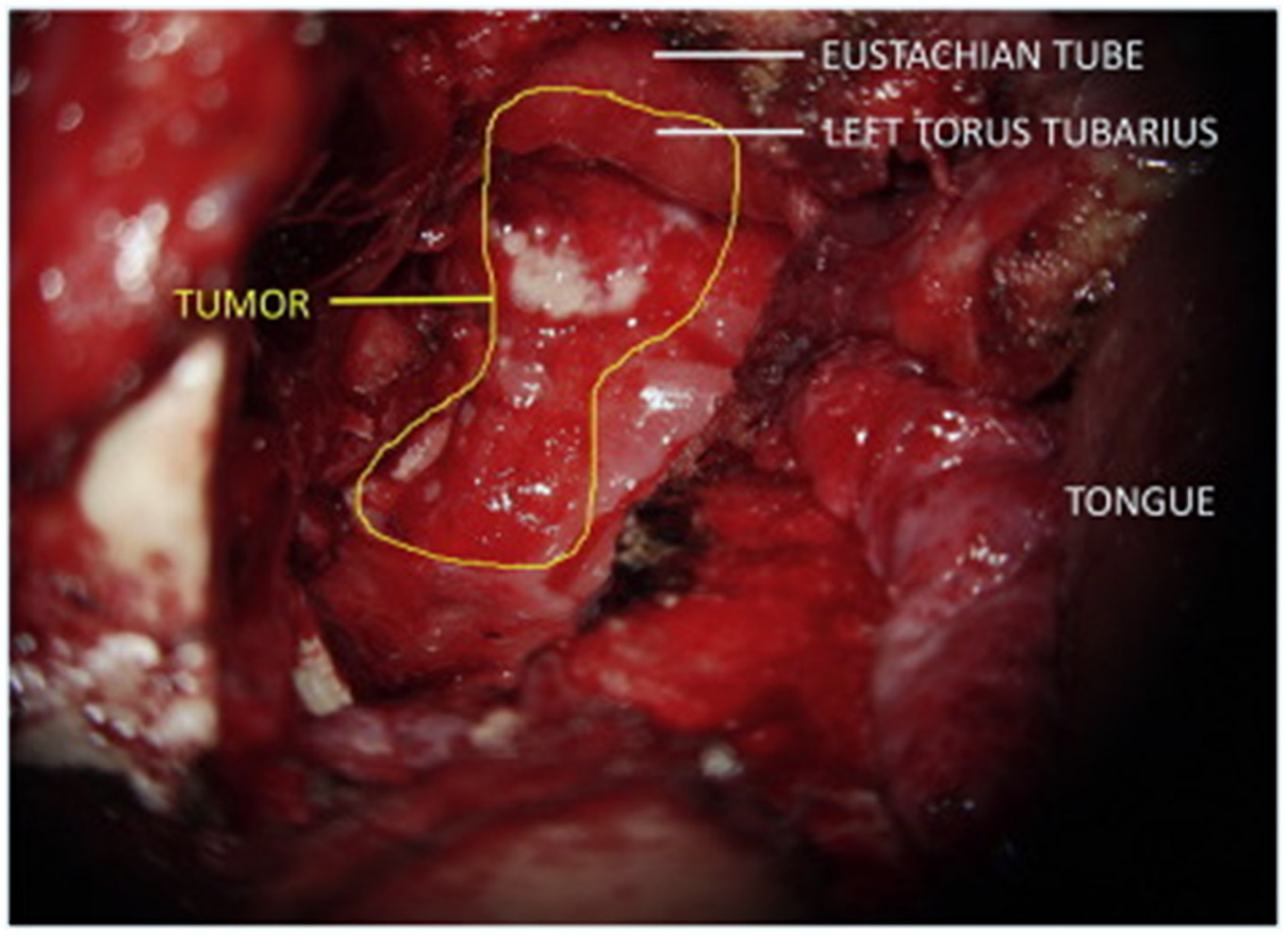
Direct view of the nasopharyngeal tumor under a surgical microscope.^89^

**Fig. 28:**
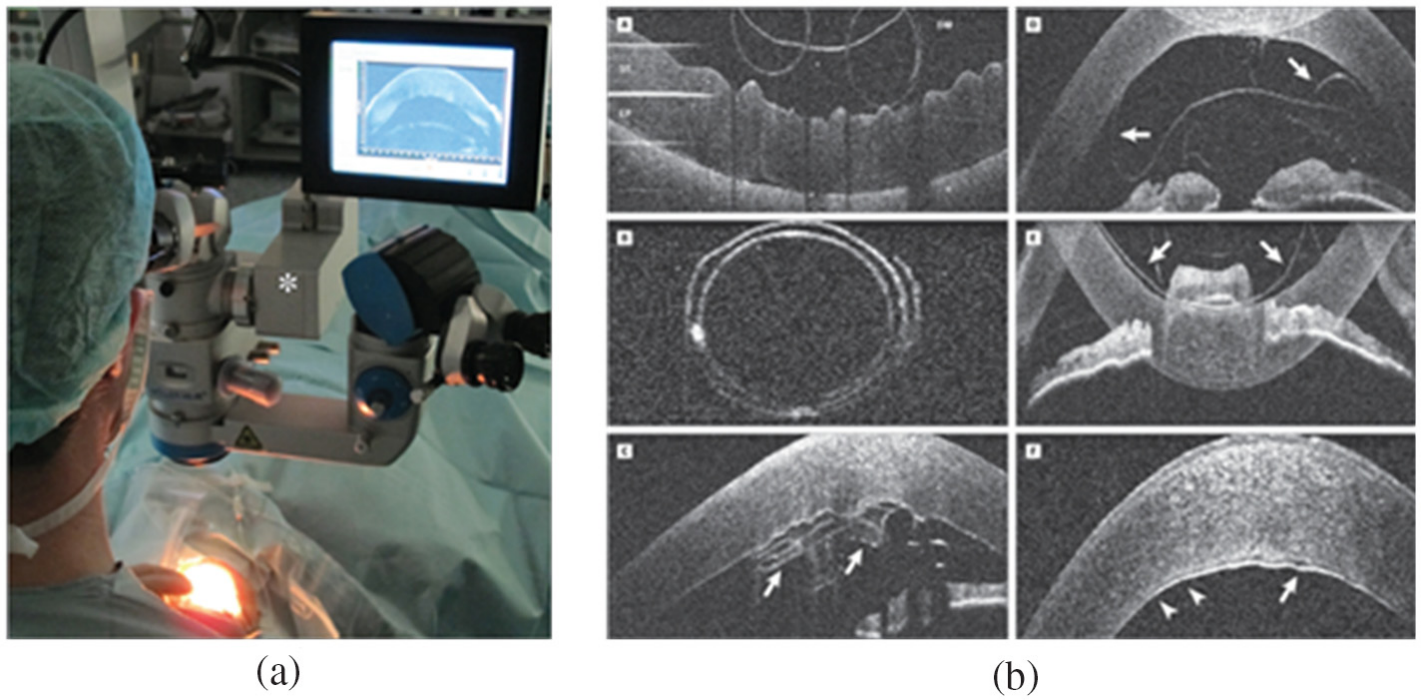
Microscope-mounted intraoperative OCT in ophthalmic surgery: (a) MMOCT setup in posterior lamellar keratoplasty and (b) iOCT improves visualization of all the procedures in descemet membrane endothelial keratoplasty.^449^

**Fig. 29:**
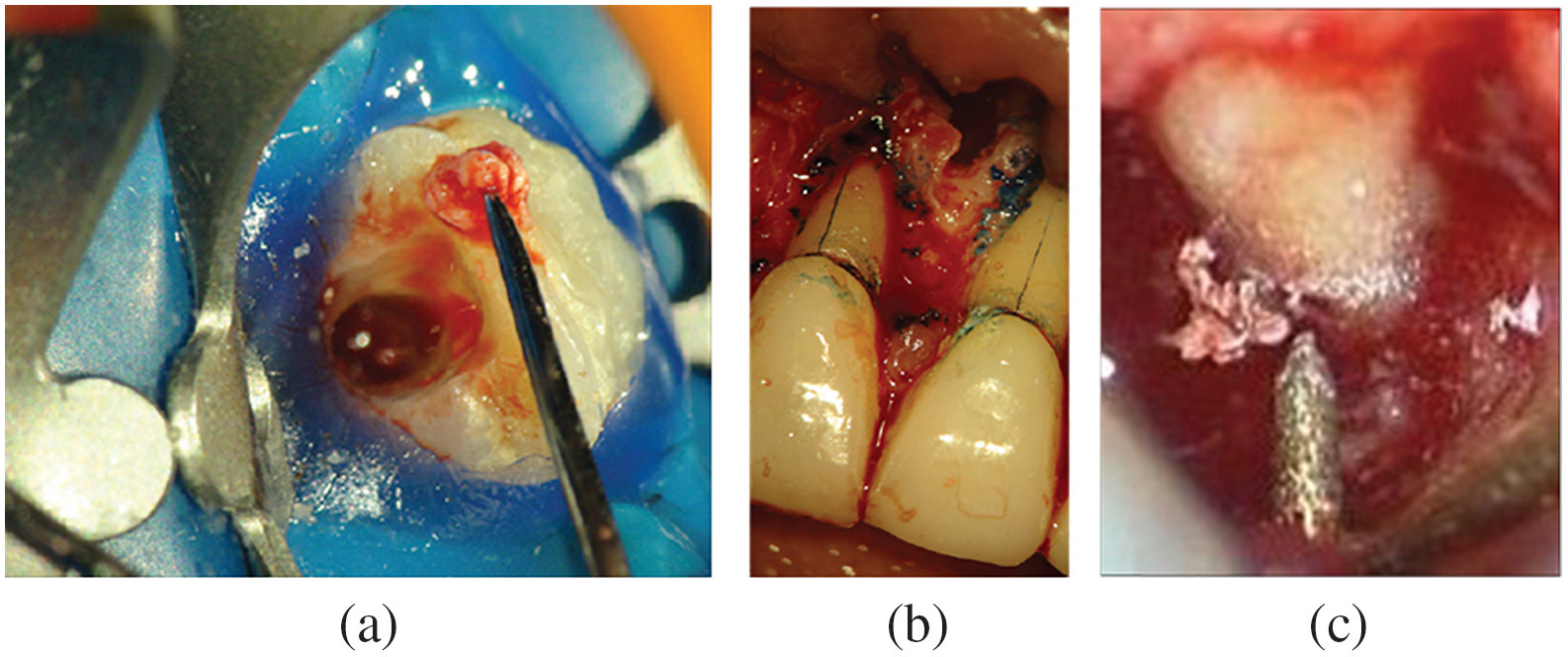
Applications of the DSM in endodontics: (a) removal of the pulp tissue, (b) vertical root fracture detected at 10× magnification with dye, and (c) root end preparation in apicoectomy 16× magnification.^73^

Microscope-integrated fluorescence-guided surgery was also applied for metastatic brain tumor resection. Okuda et al.^393^ performed 38 metastatic brain tumor resections with the aid of yellow fluorescence for visualization, and the results showed the potential to decrease the recurrence rate. Schebesch et al.^109^ used a surgical microscope with YELLOW 560 for the resection of 35 malignant brain tumors including gliomas, cerebral metastases, nonmalignant astrogliosis, and postradiation necrosis. The fluorescence signal was accessible very fast and was helpful to improve visualization of tumor margin in 28 cases. It was noted that the fluorescence effect was the most obvious in grade III and IV glioma without previous treatment. Hamamcıoğlu et al.^27^ employed YELLOW 560 in 30 surgeries of 28 patients with high-grade or metastatic brain tumors. Sodium-fluorescein was found helpful in 29 of 30 operations, and a total resection was achieved in 23 out of 29 operations. Furthermore, Kim et al.^390^ applied 32 times of intraoperative ICG videoangiography to brain tumor surgery in 23 patients, who had brain tumors including meningioma, metastasis, glioma, and hemangioblastoma. A Zeiss surgical microscope was used with the INFRARED 800 fluorescence module, which is designed for excitation in the wavelength range of 700 to 780 nm and fluorescence visualization in 820 to 900 nm. Benefiting from the high image quality and spatial resolution provided by the microscope, blood vessels were visualized with the ICG videoangiography, and important information about blood patency, blood direction, and small perforating arteries were obtained. It was proven to be extremely useful for decreasing operative morbidity and unexpected deterioration by ensuring safe manipulation of tumor-related and normal brain vessels.

Pituitary tumors happen in the pituitary gland, and transsphenoidal surgery is the most common way to remove the tumor.^398^ Fatemi et al.^399^ described their 10-year experience of direct endonasal transsphenoidal approach to the sella with the operating microscope. They had 881 operations in 812 patients with mainly pituitary adenoma, but also Rathke’s cleft cyst, craniopharyngioma, parasellar meningioma, chordoma, and others. The microscope was used for visualization of the nostril during the nasal portion and sphenoidotomy with a hand speculum, the cavernous sinus contralateral to the nostril of approach during adenoma removal, and the residual tumor with angled ring curettes. Besides, the microscope and the handheld speculum were used to explore the nasal cavities for nasal closure. The microscope could provide an effective and minimally invasive route for tumor removal. However, there were times when the microscope offered suboptimal visualization, and then endoscopic assistance could be used. The same group also investigated endonasal microscopic removal of clival chordomas,^400^ which is a skull-based tumor. The microscope was used with frameless surgical navigation in eight cases and endoscopic assistance in four cases for visualization. In addition, the microscope and the speculum were used in the direct endonasal approach, which yielded excellent access to the clivus, medial cavernous sinus, and intradural space anterior to the brainstem. The tumor removal operation using the microscope was proven to be safe and effective with gross total removal or near-total removal in 86% of patients.

##### Spine tumors

It is reported that during decompression of the spinal cord and nerve root in anterior cervical spine approaches, surgical microscopes with good illumination and magnification are helpful for surgeons to avoid complications.^25^^,^^401^ Microscopes can aid dural defects repairing and the safe use of drill and Kerrison rongeurs in bony decompression.^25^ Besides the surgical outcome, the use of microscopes in spinal surgeries also benefits surgeons’ spinal and neck health, as well as allows cosurgeons to provide meaningful assistance.^25^ Nishio et al.^402^ performed surgery of spinal cord gliomas including spinal cord astrocytic and ependymal tumors, which count for 65% of intramedullary tumors. Xu et al.^403^ performed microscopic keyhole surgery to remove thoracic spinal meningioma with a Leica M525 F40 surgical microscope. Yu et al.^404^ investigated the less invasive hemilaminectomy and hemisemilaminectomy for intraspinal extramedullary tumors of the cervical spine with a surgical microscope. Acerbi et al.^389^ reported the first experience of fluorescein-guided intramedullary spinal cord tumor (IMSCT) resection employing a Zeiss Pentero 900 microscope with the YELLOW 560 fluorescence module on 11 patients. The tumors included ependymomas, hemangioblastomas, astrocytoma, pilocytic astrocytoma, and rosette-forming glioneuronal tumors. Good fluorescent staining was obtained in nine patients, except for the astrocytoma and the glioneuronal tumor. The results have shown the feasibility of using microscope-integrated sodium-fluorescence to view the IMSCTs intraoperatively and to help achieve gross total resection.

#### 4.1.2 Neurovascular surgery

The surgical microscope is also very useful in surgeries for neurovascular diseases, such as aneurysms and arteriovenous malformation.^32^^,^^34^^,^^231^^,^^405^ The high magnification of the microscope enables safe and confident manipulation of vessels smaller than 3 mm.^406^ Microscope-integrated fluorescence modules are commonly used tools in these applications, as they are able to aid intraoperative visualization of the blood vessels and the patency of blood flow in both arteries and veins. For example, ICG videoangiography can be used for intraoperative diagnosis of sigmoid sinus thrombosis during vestibular schwannoma removal,^407^ and sodium fluorescein can be helpful for solitary brain abscess removal.^408^

##### Aneurysms

IA is a hazardous cerebral vascular disease that causes a high risk of mortality. The key to a successful aneurysm surgery is a complete occlusion of the aneurysm while maintaining the normal circulation.^87^ Dashti et al.^87^ performed intraoperative ICG videoangiography (ICG-VA) during microneurosurgical clipping of 239 IAs in 190 patients with a Zeiss OPMI Pentero microscope. The microscope-integrated ICG-VA with the magnification of microscope enabled assessment of the blood flow in the cerebral vasculature. All aneurysm domes were completely occluded in this study, with an unexpected neck residual rate of 6% and an unexpected branch occlusion rate of 6%. Rey-Dios and Cohen-Gadol^135^ used the Zeiss Pentero 900 microscope with the YELLOW 560 module to confirm aneurysm exclusion from the circulation after exposing and ligating the aneurysm. The fluorescent angiogram was able to demonstrate patency of the parent vessel and surrounding perforators as well as occlusion of the aneurysm after 20 s of fluorescein injection. De Oliveira et al.^392^ utilized ICG-VA in 60 patients with aneurysms for analyzing the presence and involvement of perforating arteries. Wang et al.^391^ employed a surgical microscope (Zeiss Pentero or Leica OH4) with the ICG-VA module on 129 patients harboring 152 IAs. They assessed the cerebral circulation in real time, and the postoperative angiographic results coincided with the intraoperative findings. Murai et al.^266^ employed a Zeiss Pentero 800 with the integrated ICG-VA module to confirm the patency of anterior communicating artery (AcomA) in five cases of aneurysm surgeries, and the surgeons have got high-quality view under the microscope in all patients. Lane et al.^409^ analyzed two microscope-integrated videoangiography techniques, namely the ICG videoangiography and fluorescein videoangiogrphy (FL-VA), for clip ligation of complex cerebral aneurysms. All 22 patients underwent both FL-VA followed by ICG-VA using the surgical microscope with YELLOW 560 and INFRARED 800 modules. The performance of the two techniques differed in patients. Compared with ICG-VA, FL-VA provided more details of perforating arteries and distal branches as well as superior image quality for the assessment of aneurysm obliteration, and it offered the advantage of real-time manipulation of the vessels. ICG-VA, on the other hand, offered better visualization of posterior communicating artery aneurysm occlusion, and it was more practical for repeat usage.

Cabrilo et al.^57^^,^^231^ reported their experience of using AR in cerebral aneurysm surgery and cerebral arteriovenous malformation surgery. They used 3D image data sets (angio-MRI, angio-CT, and 3D DSA) to create virtual segmentations, which were injected intraoperatively into the eyepieces of the surgical microscope and assisted the performance of surgical approach and optimal clipping, as shown in Fig. 25. Furthermore, Matsumura et al.^123^ described using an operating microscope system (Mitaka MM50/YOH or MM51/YOH) with higher magnifying power to improve a high-quality microsurgical clipping method for cerebral aneurysm. With the high magnification (more than 30× at any working distance, maximum 62×), the distinctiveness of the object could be as high as 7  μm. The system was applied for microsurgical clipping of cerebral aneurysms in 11 patients, and all aneurysms were treated without complications. The microscope with high magnification was proposed to be beneficial for safe and precise operation. The system was also evaluated in extracranial-intracranial bypass surgery, where the high magnification allowed visualization and manipulation of small vessels.^410^

##### Arteriovenous malformation

Fukuda et al.^34^ performed seven cerebral arteriovenous malformation surgeries with Zeiss Pentero surgical microscope and FLOW 800 for ICG videoangiography. By assessing the transit time at different phases and the fluorescence intensity, they have concluded that FLOW 800 analysis with ICG-VA is feasible for a more safely and convincingly resection of AVM. Similarly, Takagi et al.^405^ performed 27 ICG videoangiography procedures in 11 patients with FLOW 800 and obtained an improved visualization especially of the nides and superficial drains, as well as the flow changes after clipping or coagulating of feeders, as shown in Fig. 26. Killory et al.^265^ evaluated microscope-integrated ICG angiography intraoperatively, and the results showed less favor of ICG for deep-seated lesions and AVM vessels under surface. Rey-Dios and and Cohen-Gadol^135^ investigated using Zeiss Pentero 900 and YELLOW 560 in AVM surgery. Fluorescence appeared after 20 s of fluorescein administration, demonstrating the angioarchitecture of the AVM. The lack of cortical arterial feeders was confirmed with the first injection, and the presence of additional arterial feeders was recognized with a second fluorescein injection.

##### Arteriovenous fistulas

Killory et al.^411^ employed an NIR video-integrated surgical microscope with integrated ICG fluorescent angiography function in three cases of spinal dural arteriovenous fistulae (dAVF) surgeries, where the intradural vein draining the fistula was rapidly and clearly distinguished from the artery or uninvolved medullary vein. Hanggi et al.^412^ also evaluated microscope-integrated intraoperative ICG-VA for dAVF and AVM. Out of 46 ICG-VAs, 41 investigations had excellent image quality and spatial resolution to analyze different structures, and additional information was obtained in two cases to change the surgical strategy.

##### Cavernous malformation

Ungersböck et al.^413^ performed surgery on CMs using an operating microscope with frameless stereotactic guidance. They have reported advantages of using frameless stereotactic localization as sufficient accuracy, utility, ease of integration, and detailed anatomical information. Endo et al.^414^ investigated ICG videoangiography in eight cases of intramedullary CM surgeries, where CMs were seen as avascular areas and venous structures were delineated on ICG videoangiography. The results have shown the potential for ICG to assist safe and complete removal of CMs.

##### Moyamoya disease

It is a cerebrovascular disorder caused by blocked arteries at the base of the brain. Yabumoto et al.^415^ reported a surgery of moyamoya disease associated with multiple IAs. A surgical microscope was used after a right frontotemporal craniotomy to recognize a few abnormal vascular networks consisting of small arteries and veins. Then, the aneurysms were clipped and unruptured to recover the blood flow to moyamoya vessels.

##### Stroke

Stroke happens when the blood flow gets a sudden interruption due to obstruction or rupture of the arteries supplying the brain. Kruijff et al.^416^ reported an operation for stroke by joining an artery in the scalp to one on the surface of the brain using an operating microscope and microinstruments. The microscope was used to examine the cortex, prepare the end of the donor artery, and join the arteries. Magnification of 16× to 25× was used for making an oval opening of the donor artery, and high magnification of 25× to 40× was for the anastomosis. The blood flow was then redirected from the artery in the scalp into the arteries that supply the brain.

### 4.2 ENT

Otorhinolaryngology surgery, in other words, ENT surgery, is another surgical field where surgical microscopes are used. Because of the deep cavities and subtle structures existing in these areas, magnification and bright illumination are vital for the surgical outcome. The microscope also offers a variable working distance that is appropriate to the depth of approaches in the ENT surgeries.^387^ Despite that endoscopes sometimes can offer a better view of the narrow operating field,^417^^,^^418^ binocular surgical microscopes provide depth perception and the possibility of bimanual manipulation of tissue.^7^^,^^88^ Furthermore, with the operations becoming more complicated and sophisticated, AR and various imaging modalities are combined with surgical microscopes for surgeons to conduct image-guided surgery.^238^

#### 4.2.1 Otology

Surgical microscope was first applied in aural surgery^20^ in 1921, and it has then been utilized for a large portion of otology operations,^26^ including diagnosis of the tumor in middle ear,^419^ dissection of the temporal bone,^420^ examination of the drum position in atelectatic ears,^421^ and laser surgery for chronic ear disease and otosclerosis treatment.^422^ Its main advantage is the improved visualization, which results in other benefits including precise surgical manipulation, smaller instruments, and less trauma.^26^ It enabled the discovery of the minute structures in the middle and inner ear and offered the possibility to master the complicated surgical anatomy of the temporal bone.^26^^,^^423^

Nowadays, new technologies combined with surgical microscopes are being evaluated for ear surgery to aid the visualization of ear structures. Lankenau and Pau^296^^,^^424^ investigated using a surgical microscope with microscope-mounted OCT (MMOCT) for anatomical orientation in cochlear implant surgery. The system was able to reveal the cochlear morphology without opening the enveloping membranes. The OCT images supplied information about inner ear structures, showing the potential of providing an orientation guide to precisely localize the scala tympani before opening the fluid-filled inner ear. Cho et al.^295^ developed an AR system combining OCT with a surgical microscope. The system was able to simultaneously acquire *in vivo* microscopic and OCT images of middle-ear and inner-ear microstructures. AR technique was utilized to show both images overlaid in the left eyepiece. The system was tested on a mouse and was able to show monolayers of each microstructure in real time.

#### 4.2.2 Rhinology

The development of surgical microscopes has opened new ways for functional approaches and less radical surgery of rhinology.^425^ The nasal fossa and paranasal sinus, where the narrow port of entry, deep situation, and complicated anatomy exist, are ideal areas for microsurgery.^426^ The use of a surgical microscope in nasal surgery was evaluated and proposed to be an alternative to the traditional endoscopic method.^88^ The surgical outcome and the feedback from patients proved that microscopic surgery was well tolerable and superior to other surgical procedures. With the aid of an autostatic speculum, surgeons were able to work bimanually, which was the main advantage. In addition, the widefield stereoscopic vision, magnified operating field, excellent illumination, as well as adequate exposure of nasal and paranasal cavities, allow a more panoramic view and additional precision with good healthy tissue preservation and less surgical complication. Amedee et al. performed microscopic endonasal surgery for the treatment of paranasal sinus^427^ and chronic sinusitis with polyps.^428^ In addition to the aforementioned advantages, they also mentioned the easy control of operative hemorrhage with appropriate tools as well as the outstanding opportunity for resident teaching. Teatine et al.^429^ performed transnasal sinusectomy with either a microscope technique or a combined microscopic and endoscopic technique. The surgical microscope was used in ethmoidectomy and to open the sphenoid sinus and the antral window, while the endoscope was used for cleaning and drainage. Surgeries carried out with the microscope technique had minor complications, and the combination technique resulted in trivial complications. Testa et al.^430^ coupled a carbon dioxide laser with a surgical microscope for turbinate surgery. The laser was sighted through the microscope and cut the obstructive rhinitis tissue with a small wound. Dietrich et al.^431^ employed a surgical microscope instead of an endoscope in endonasal dacryocystorhinostomy, due to its advantage of increased precision by allowing bimanually work while drilling bone from the frontal process of the maxilla. Kumar et al.^5^ performed preauricular sinus surgery using an operating microscope. The magnification of microscope, used along with other instruments, was helpful to provide a bloodless field and enable precise dissection without any epithelial breach.

#### 4.2.3 Laryngology

Surgical microscopes can aid visualization during laryngeal surgery, for example, Hazarika et al.^432^ used a surgical microscope in KTP laser-assisted cricopharyngeal for observation of posterior muscular bar. Welham et al.^433^ used a surgical microscope in graft implantation surgery for the treatment of vocal scar and pathologic sulcus vocalis. After exposing the true vocal fold superior surface, the microscope was used in all subsequent procedures, including the incision, a sharp dissection, and graft material implantation. Various imaging techniques have also been evaluated for laryngeal applications. Just et al.^298^ employed MMOCT intraoperatively in the human larynx. Images of the true vocal folds were acquired, where surgeons identified basement membrane, gelatinous fluid, and regions with remaining fluid chambers. It was noted that MMOCT could describe the microanatomy of the healthy larynx and benign lesions, as well as enable early detection of microinvasive carcinoma. Martin et al.^46^ developed a hyperspectral surgical microscope and captured hyperspectral images of *in vivo* human laryngeal mucosa of five patients. With the image classification algorithms, they achieved automatic detection of the hemorrhagic polyp and leukoplakia in the vocal cord and showed the potential of this system to provide noncontact optical biopsy.

#### 4.2.4 Head and neck surgery

Surgical microscopes can be used for the resection of various head and neck tumors, as well as the head and neck endocrine surgeries for thyroid and parathyroid. The advantages of the surgical microscope for head and neck surgeries, except for the magnification, illumination, and stereoscopic view, lie in the safe manipulation of the to-be-resected tissue while achieving good preservation of the normal tissue or nerve.

##### Head and neck cancer

Surgical microscope has been utilized in the surgical treatment of various head and neck cancers, such as the resection of nasopharyngeal tumor,^89^ oropharyngeal tumor,^434^ parotid tumor,^387^ and the submucosal microdissection of laryngeal chondrosarcoma.^435^ The use of the surgical microscope provides superior visualization of the surgical site, which results in an ideal tumor dissection with satisfying preservation of healthy tissue.^435^

Jang et al.^89^ studied the feasibility of performing open nasopharyngectomy with an operating microscope (NOM) via the maxillary swing approach for the resection of nasopharyngeal tumors. After exposing the posterior nasal space, the microscope was positioned directly over the surgical site, of which surgeons could have a direct view, as Fig. 27 shows. The use of the microscope aided better visualization of the tumor margin and critical structures, thus enabled adequate resection margin and fewer surgical complications.

Transoral surgery is an attractive approach for the treatment of oropharyngeal cancer.^434^ Surgical microscopes can be coupled with ${\mathrm{CO}}_{2}$ laser for transoral laser microsurgery,^7^^,^^434^ which is a minimally invasive option for complete primary tumor resection and maximal preservation of healthy tissue.^436^ Because the transoral method can only reach the small oropharyngeal tumors in the tonsil, soft palate and posterior pharyngeal wall, surgical microscopes in this situation can provide necessary magnification for excellent direct visualization of the exposed surgical site.^7^^,^^436^ Surgeons therefore can discern clearly between the healthy tissue and tumor at the perimeter of the resection bed, which enables precise manipulation and excision of tumor.^437^

Surgery at parotid involves peripheral nerves. Magnification is essential for the identification of exposed nerve fascicles.^438^ Carta et al.^387^ reported performing microscope-assisted parotidectomy for parotid tumor resection with the aid of intraoperative nerve monitoring. The surgical microscope was utilized during facial nerve dissection, where the terminal branches of the facial nerve were finely discriminated from the vessels and the salivary ducts. It resulted in a fine dissection and reduced postoperative facial nerve dysfunction rate. Rigante et al.^438^ used a surgical microscope during the intracapsular enucleation for the treatment of intraparotid facial nerve schwannoma (IFNS), which resulted in good nerve functionality preservation.

##### Head and neck endocrine surgery

Microscope-assisted thyroidectomy has been reported as a reliable, safe, and potentially advantageous method.^439^^,^^440^ Using the surgical microscope in thyroidectomy can aid visualization of recurrent laryngeal nerves (RLNs), as well as facilitate diligent dissection, which results in significantly lower rates of transient hypocalcemia and reduced rate of RLN injury than conventional thyroidectomy.^441^^,^^442^ It could also prevent inadvertent injury to adjacent structures during crucial dissection steps, notably around parathyroid glands and their vascular pedicles.^440^^,^^443^ Davidson et al.^64^ evaluated the safety and efficiency of adopting a surgical microscope in thyroidectomy. The superior magnification and illumination were more than adequate to replace the surgical loupes and headlight. Although the operating time of using the surgical microscope in the malignant cases was longer, they did not observe any significant difference in operating time in the benign cases.^64^^,^^440^ Nielsen et al.^444^ were able to expose the nerve in its full length and remove all thyroid tissue with no residual by using the microscope. Besides, the microscope could bring ergonomic benefits, which helped surgeons maintain an upright posture with a neutral cervical position.

### 4.3 Ophthalmology

Human eyes are sophisticated, thus ophthalmic surgery requires delicate work, which has brought high standard technical requirements for ophthalmic surgical microscopes. The high magnification of the microscope provides surgeons with better visualization and more exact surgical operation of the small structures and the incision.^121^^,^^445^ For example, in femtosecond laser keratotomy, surgical microscopes can offer high magnification to observe the anterior chamber,^446^ center the eye^447^ for incision, and check the position of cut.^448^ In ophthalmic surgery, illumination is critical, not only to provide the best visualization with low light intensity but also to create red reflex, which is a specific reddish reflection of light that helps throughout cataract procedures. In addition, OCT is widely used in ophthalmic procedures for subsurface visualization. It provides cross-sectional images that overcome the deficiency visibility of the delicate tissue with the microscope itself due to its *en face* view. With the optional magnifications of the microscope, iOCT can acquire images of different lateral sizes depending on the need of target.^449^ Ophthalmic microscopes are often equipped with intraoperative OCT functions so that no cessation of procedures will be required for imaging. Ophthalmic surgical microscopes and MIOCT systems have been actively investigated in various applications, such as cataract intraocular implantation,^450^ corneal penetrating keratoplasty,^37^ and vitreoretinal macular hole repair.^90^ Here, we discuss two big applications of ophthalmic surgical microscope and MIOCT, namely corneal surgery and vitreoretinal surgery.

#### 4.3.1 Corneal surgery

Ophthalmic surgeries of the anterior segment of the eye require clear visualization of the delicate structures such as the iris and cornea. Besides, safe surgical manipulations such as incision and unfolding demand real-time image guidance. Geerling et al.^305^ coupled a commercial OCT system with a surgical microscope (Hi-R 900, Moller-Wedel) and tested it for intraoperative visualization of sclera and cornea during lamellar keratoplasty and trabeculectomy. The anterior segment structures such as cornea, conjunctiva, sclera, and iris were seen through the microscope-mounted OCT. During a trabeculectomy, the thickness of the scleral flap could be measured with the MMOCT; and during deep lamellar keratoplasty, the MMOCT was able to show the abnormal cornea and the lamellar interface. Steven et al.^172^^,^^449^ employed a commercial microscope-mounted SDOCT system (iOCT, OptoMedical Technologies GmbH) with a surgical microscope (Hi-R 900 A NIR, Moeller-Wedel), as shown in Fig. 28(a), and evaluated the system in 26 patients under-going Descemet’s membrane endothelial keratoplasty (DMEK) and 6 patients undergoing deep anterior lamellar keratoplasty (DALK). All procedures in DMEK were benefited by the MMOCT, particularly the visualization of crucial steps such as graft rolling, unfolding, and orientation, as shown in Fig. 28(b). In DSLK, iOCT was helpful to verify trephination depth and needle insertion for identification of the accurate preparation depth, thus to assist in manual trephination. Furthermore, Siebelmann et al.^58^ mounted an SDOCT camera (iOCT, OptoMedical Technologies GmbH, Lübeck, Germany) on a Hi-R Neo 900A NIR surgical microscope (Haag-Streit Surgical GmbH, Wedel, Germany) and evaluated the MMOCT in two Boston keratoprosthesis (KPro) surgery cases.^58^ All parts of the prosthesis and the interfaces between corneal graft and implantation were well visualized. Moreover, the iOCT revealed two gaps that were not visible with a microscope alone.

Since microscope-integrated iOCT systems became commercially available, several studies have been evaluating MIOCT in different ophthalmic applications. Cost et al.^288^ employed a Zeiss surgical microscope and RESCAN 700 for iOCT imaging in eight cases of DMEK surgeries during the host and donor tissue preparation, graft orientation, graft apposition, and tissue interface fluid dynamics. Pasricha et al.^289^ reported using RESCAN 700 in two cases of Descemet’s stripping automated endothelial keratoplasty (DSAEK). The MIOCT helped surgeons to see the structures past the anterior cornea that was negligible with standard microscope illumination. In addition, surgical maneuvers such as graft insertion, unfolding, tamponade, and attachment were clearly visualized. Kobayashi et al.^37^ used RESCAN 700 for rapid visualization and rough evaluation of the donor tissue for penetrating keratoplasty (PK), precut DSAEK, and prestripped DMEK donor tissue. Sharma et al.^451^ used RESCAN 700 for the management of Descemet’s membrane detachment after DALK. In addition, direct visualization through the microscope was utilized when surgeons injected gas and monitored the height of the detached Descemet’s membrane. Eguchi et al.^42^ performed PK and DALK and used Zeiss Lumera 700 microscope with RESCAN 700 to detect iris incarceration and iridocorneal adhesions intraoperatively. Furthermore, Sharma reported a new surgical technique as MIOCT-guided small-incision lenticule extraction. During the surgery, MIOCT showed the location and thickness of the lenticule, revealed the relationship of the anterior-posterior lamellar planes, and helped surgeons achieve the desired plane of dissection by tracking the instrument tip in the intraoperative images.

#### 4.3.2 Vitreoretinal surgery

It is considered routine to use the surgical microscope in modern vitreous and retinal surgeries.^452^ For the posterior segment, the ophthalmic surgical microscope, as well as MIOCT, is useful to visualize the morphological changes of the retina, for which typical clinical applications include scleral wound, proliferative retinopathy, vitreomacular traction, myopic foveoschisis, macular hole, retinopathy of prematurity, and retinal detachment.^38^

Zhang et al.^452^ investigated the feasibility of performing scleral bucklings for retinal detachment with a surgical microscope instead of the routinely used ophthalmoscope. The clear, upright image with controllable magnification enabled easy observation and examination of retinal breaks and degenerations. In the total 342 cases of surgeries performed under the surgical microscope, surgeons found 39 eyes where additional or new retinal breaks were found. Moreover, it was said that the routine use of surgical microscopes could help surgeons protect the vortex and prevent inadvertent scleral perforation. Fleming et al.^9^ developed an AR surgical microscope system for retinal surgery, which fused preoperative fundus image and OCT image with intraoperative video to increase targeting accuracy. Ray et al.^90^ mounted a handheld SDOCT device, i.e. the Bioptigen system (Bioptigen, Inc; InVivoVue Clinic v1.2), onto an ophthalmic surgical microscope. The system was used to image the macular hole and the epiretinal membrane of 25 eyes in 24 patients who underwent macular surgery. It captured images pre-incision, post-hyaloid elevation, post-residual epiretinal membrane peel, and post-internal limiting membrane peel. After image analysis, the intraoperative change of macular hole dimension and retinal thickness that were caused by surgical manipulation could be measured. Grewal et al.^453^ performed an Argus II implantation with a prototype microscope-integrated SDOCT system. The system was used for the visualization of preretinal tack placement, retinal tack placement, and postretinal tack placement. It was said that MIOCT allowed confirmation of array position placement and confirmed the adequacy of tacking in securing the array. Chen et al.^454^ performed intraoperative MIOCT angiography on a 2-year old kid who underwent vitrectomy and a 7-month-old infant who was diagnosed with familial exudative vitreoretinopathy. Jayadev et al.^38^ and Kumar et al.^455^ reported using RESCAN 700 as a surgical tool to image surgical maneuvers during vitreoretinal surgery. For example, it has the capability to image the anatomical configuration of the scleral wound, determine residual traction membranes in proliferative retinopathy, assess the strength of vitreomacular adhesions in vitreomacular traction, avoid iatrogenic break of myopic retinae in myopic foveoschisis, and avoid inadvertent nerve fiber break by providing real-time imaging of membrane peeling in the cases of macular holes. For retinal detachment, the MIOCT can detect residual subretinal fluid and completion of fluid air exchange. As for retinopathy of prematurity, it is able to assist surgeons to determine the extent of horizontal and anteroposterior tractional elements.

A group at Duke University and Duke University Medical Center has done massive work on microscope-mounted OCT imaging. They have demonstrated the use of a surgical microscope with a microscope-mounted SD-OCT prototype for the visualization of vitreoretinal surgical procedures,^307^ including retinal effects of surgical contact, primary static surgical steps,^306^ and surgical instrument as well as their intraoperative motions.^308^ In the study of retinal imaging,^306^ the MMOCT system acquired *in vivo* human retinal SDOCT images for observation of the posterior pole. Retinal architecture as well as macula and optic nerve could be well visualized in the B-scans. Nonmetallic instruments were better visualized than metallic ones in the B-scans. Intraoperative manipulations of the retina as well as the retina effects were successfully imaged. The PIONEER study^456^^,^^457^ evaluated a microscope-mounted portable SDOCT system for both anterior and posterior segment surgeries. DSAEK and vitrectomy with membrane peeling were the most common surgeries performed for anterior and posterior segments, respectively. During pars plana vitrectomy surgery, iOCT could help reveal epiretinal membrane, macular edema, posterior hyaloidal traction, and retinal detachment. It was noted that in 48% of lamellar keratoplasty cases and 43% membrane peeling cases, surgeons were informed of surgical decision making by the MMOCT, and their understanding of underlying tissue configurations was altered. In addition, the microscope afforded iOCT axial stability as well as good control of the X-Y-Z translation movement by a foot pedal.

Furthermore, in a 3-year DISCOVER study,^36^^,^^290^ an MIOCT was used in ophthalmic surgeries of both anterior and posterior segments. Among three commercially available prototype MIOCT systems, namely Zeiss RESCAN 700, Leica EnFocus, and Cole Eye iOCT, the RESCAN 700 MIOCT system was evaluated with a Zeiss Lumera 700 surgical microscope by multiple surgeons in 244 eyes with anterior segment cases and 593 eyes with posterior segment cases. The Z-tracking and focus control of RESCAN 700 provided good image quality and stability. For imaging of the anterior segment, a standard microscope viewing system was used, while for the posterior segment, the RESIGHT lens system or a contact lens was used. The images were captured to evaluate corneal incision, scleral closure, phacoemulsification groove depth, intraocular lens position, epiretinal membrane, vitreomacular traction, and hyaloid release with triamcinolone and completeness of peel in the macular hole. It was reported that the iOCT information impacted surgeons’ decision making in 43.4% of anterior segment surgeries and altered surgical decision making during the procedure in 29.2% of posterior segment surgeries.

### 4.4 Dentistry

The adoption of magnification in dentistry was slower than that in neurology and otolaryngology until the improvement of ergonomics and treatment outcome was commonly recognized. DSMs being the most important revolution in dentistry,^72^^,^^130^^,^^458^ have greatly improved dentists’ differentiation ability, comfort, and reliability of diagnosis therapy.^11^^,^^73^^,^^130^^,^^459^^,^^460^ Meanwhile, it made the procedures minimally invasive, less painful, and less trauma to patients.^130^ Moreover, it enables easy digital documentation and the use of finer instruments^76^^,^^126^ and achieves superior esthetic outcomes.^73^ The high magnification (20× to 25×) of a DSM can provide over 10 times the information as that of a 3X loupe,^126^ and the distinct coaxial illumination brought by surgical microscopes is superb solutions for deep cavities and shadows.^130^^,^^459^ A microscope with a straight tube binocular can facilitate clinicians to look down the axial plane of the root in maxillary teeth and up the axial plane of the root in mandibular teeth, providing direct vision regardless of the position of patient.^130^ Two surveys indicate that the accessibility and use of DSM by endodontists increased from 52% in 1999 to 90% in 2008.^459^ Applications of surgical microscopes in dentistry include diagnosis, nonsurgical and surgical endodontics,^114^^,^^130^^,^^461^ as well as periodontics.^122^ In nonsurgical procedures, microscopes aid easy observation and management; while for surgical endodontics, it allows thorough examination, enhances resection, and allows easy preparation.

#### 4.4.1 Endodontics

DSM can be employed diagnostically for multiple applications, such as visualizing the root canal system in fine detail, examining dental caries and insufficient crown margins, assessing marginal integrity of restorations, detecting cracks or microfractures, locating canal orifices, observing sub-gingival defects, and observing complex anatomical situations, etc.^11^^,^^72^^,^^114^^,^^130^^,^^460^^,^^462^ High magnifications of DSM is beneficial in nonsurgical procedures for identification of canal bifurcations, identification and removal of canal obliteration and calcifications, and obturation.^11^^,^^130^^,^^181^^,^^459^ The color and texture of dentin become apparent under the microscope, guiding practitioners to root canal orifices or allowing safer dentin removal.^114^^,^^130^^,^^459^ Magnification allows endodontics to better visualize pulp chamber^463^^,^^464^ and canal orifices and improves the efficiency of instrumentation.^76^^,^^130^ Moreover, surgical microscopes can help with the retrieval of broken instruments in the canal and pinpoint precisely.^72^^,^^75^

For surgical endodontics, DSM has become one of the state-of-art landmarks and an invaluable instrument,^11^^,^^130^ especially in difficult treatments where high-level magnification is needed, such as root-end preparations and root-end fillings.^74^^,^^465^ It is predominately recommended for osteotomy, curettage, apicoectomy, apical preparation, and retro filling.^130^ Other endodontic microsurgery steps carried out under various magnifications including root-end resection and inspection of resected surface, detection of apical perforation, microsurgical flap preparation, and microsurgical suturing were also greatly enhanced by the microscopic approaches.^130^ In addition, the microscope is helpful in endodontic retreatment procedures to identify and remove leftover filling materials, and in nonsurgical perforation repair procedure to place the perforation repair material more precisely.^459^^,^^460^ The goal of utilizing a microscope in surgical endodontics is to achieve the highest possible precision as well as the maximum protection to healthy tissue.^130^ Figure 29 shows some of the important applications of a dental surgical microscope. DSM and microscopic approaches have completely transformed surgical endodontics from a blindfold work to a vision-based work.^74^^,^^182^^,^^459^ The advantages are rapid wound healing, fewer postoperative pain, and better prognosis. In addition, two reviews of surgical endodontic outcomes^466^^,^^467^ showed that the utilization of microscopes is associated with significantly better outcomes than loupes.

During root canal therapy, microscopes can greatly aid caries removal, calcification removal,^91^ canal orifices localization,^464^^,^^468^ and internal resorption treatment with magnification and illumination.^459^ In a study of 184 cases of necrotic teeth with chronic periapical lesions, the impact of the DSM was evaluated with two groups (using a microscope and conservative), and the microscope was proved to be helpful with a significant statistical difference of outcomes (p=0.0078). In addition, it was reported to be extremely useful in removing obstacles from the pulp chamber or root canal system and locating calcified or missed canals.^458^ Wu et al.^91^ evaluated the clinical application of the surgical microscope in the management of root canal therapy of 546 root canals. A Zeiss OPMI pico microscope, as well as a rubber dam and an ultrasonic instrument, was used in the therapy. Under the microscope, the calcified canal orifices were located, and the colors of the normal chamber floor and the calcified dentine were identified, which were the first key aspects in the treatment. It also aided the detection of perforation and instrument separation. With the help of the microscope, 33 out of 40 root orifices that were not detected with naked eyes were successfully detected. Karapinar-Kazandag et al.^469^ assessed the ability to detect and negotiate the accessory mesial canals in mandibular molars using either a surgical microscope or loupes. Comparing with loupes, the use of the microscope increased the number of detected accessory mesial canals by 6% in the second molar and 2% in the first molar, while comparing with no magnification, the number increased by 17% in the first molar and 5% in the second molar. The microscope also benefited the negotiation of accessory mesial canals with an 8% increase in the second molar and 2% in the first. Mitsuhashi et al.^470^ investigated the marginal adaptation and microleakage of MTA root fillings under unaided vision, dental loupes, and the surgical microscope. They reported that using the microscope left no gap between the MTA and dentin at any magnification, while a clear gap was revealed in the unaided vision group at 5× to 50× magnification and in the loupe group at 50× magnification. In addition, the filling procedure using microscope was able to block the dye penetration, which was significantly different from the deep penetration using unaided vision.

In a case study of 50 apicoectomies, DSM facilitated each phase of endodontics surgery, and the postoperative evaluation showed a reduced incidence of symptoms and less postoperative pain in the cases treated with microscope.^471^ Two surveys of apical surgery using DSM reported 96.8% healing within 1 year, and 91.5% healing in the following 5 to 7 years, well beyond the success rate of conventional apicoectomys.^472^ In endodontic retreatment, especially the critical phase of removing the filling material, the surgical microscope can give a direct view of the root canal thus aid the removal of silver cone.^473^ High magnification of the microscope is also essential in laser dentistry. During noncontact soft tissue ablation, it provides accurate visualization of laser-tissue interaction to eliminate the risk for accidental interaction of laser and tooth; and for hard tissue laser ablation, it replaces the tactile method and helps determine complete decay removal.^126^

#### 4.4.2 Periodontics

In a well-established periodontal microsurgical practice, 70% to 80% of the typical periodontal microsurgeries can be done at a magnification of 10× to 20× with a surgical microscope, while the rest can be done with a surgical loupe.^153^ Surgical microscopes may represent a useful tool when particular esthetic demands require maximum precision.^474^ The use of surgical microscopes in periodontal microsurgery^475^ can lead to cleaner incision, closer wound apposition, reduced morbidity, reduced hemorrhage, and less trauma at the surgical site. The magnification reveals the fact that many gentle maneuvers with the naked eye turn out to be gross crushing and tearing of the delicate tissue,^475^^,^^476^ and also adds precision to the repositioning of tissue by allowing smaller needles and sutures.^477^ Meanwhile, it enables fast wound healing and less inflammation.^122^^,^^478^

Applications of the surgical microscope in periodontal therapy include root surface debridement,^104^ implant surgery,^479^ minimally invasive periodontal regeneration,^480^ and mucogingival surgery.^474^ Visual access to the root surface is determinant for the success of periodontal therapy, and it is more efficient under higher magnification for the removal of residual calculus as well as achieving a clean and smooth root surface.^481^ In implant therapy, high magnification provided by the microscope can help the implant site development and placement, achieving higher precision.^73^^,^^475^ The surgical microscope is beneficial at different stages of implant treatment from implant placement to implant recovery. It also brings more precision to the peri-implantitis management^153^ and aids indirect visualization of the sinus membrane in the sinus life procedure.^153^^,^^475^ The use of the surgical microscope in periodontal regenerative surgery can increase the capacity to manipulate the soft tissue, resulting in an improved potential for primary closure of the wound to 92% of microsurgery from 70% of regular surgery.^482^ It also allows a smaller incision to gain surgical access and debride the periodontal defect prior to placing the graft.^483^ Mucogingival surgery is also called periodontal plastic surgery.^484^ For some procedures in periodontal plastic surgery that can be very difficult with naked eyes, such as papilla reconstruction, the DSM can turn it into an easy one by increasing visibility, eliminating unnecessary incision, and facilitating access.^475^^,^^485^ Besides, it enables minimally invasive techniques that can minimize trauma, allow primary wound closure, and improve facial esthetics.^486^

The use of the surgical microscope has been actively evaluated in root coverage procedure, which is a type of mucogingival surgery for the treatment of gingival recession. It enables excellent visualization and an atraumatic surgical approach in root coverage procedures, which results in satisfying cosmetic outcomes such as better searing and marginal profile.^474^ When the procedure is combined with coronal flaps, the microscope also makes the advancement of flaps easier and tension free.^476^^,^^481^^,^^487^ Bittencourt et al.^92^ compared surgical outcomes of root coverage procedure with subepithelial connective tissue graft with or without a surgical microscope. The test group using the microscope significantly outperformed regarding the average percentage of root coverage, complete root coverage, and patient esthetics satisfaction. Thankkappan et al.^488^ performed root coverage procedures using subepithelial connective tissue graft and collagen membrane with a surgical microscope. The 6× magnification delivered precision and visual acuity, which guaranteed precise control of the gingiva. Burkhardt and Lang^489^ employed a Zeiss OPMI Pro magis DSM for recession coverage at 15× magnification, while the graft and palatal closure ahead of it were performed with a prism loupe at 5×. Meanwhile, surgical microscope OPMI FR was used for fluorescence angiography. The microscopic approach was compared in the study with a conventional macrosurgical approach. The results showed that the microscope improved visual and tactile approach, and it enabled the use of finer instruments and suture materials, which reduced tissue damage. In addition, the magnification was able to help split a flap in a well-defined thickness, which resulted in decreased vessel injury. The angiography revealed a better vascularization of microscopic approach than the macrosurgical one.

### 4.5 Plastic and Reconstructive Surgery

Plastic and reconstructive surgery is another big area where the surgical microscope is employed. The surgical microscope does not necessarily offer significantly better surgical outcomes in every plastic application,^93^^,^^490^^,^^491^ but it stands out with some advantages that other methods do not possess. Actually, the high and varied magnification and the excellent illumination provided by the microscope are critical and beneficial in many cases, such as the optimal alignment of nerve ends,^492^ the precise reconstruction of uterine tube,^81^ and fallopian tube,^493^ as well as the minimally invasive excision of small structures such as ingrown toenails.^85^ Besides, other features including the share of view with assistant and staff team, recording, and position of comfort can also facilitate a smooth surgical workflow.^117^^,^^494^ In this section, we will discuss several applications of the surgical microscope in plastic and reconstructive surgeries, including craniofacial surgery, reconstructive microsurgery, and dental plastic surgery.

#### 4.5.1 Craniofacial surgery

Craniofacial surgery involves the congenital or acquired deformities of the skull, head, face, neck, jaws, and the associated structures, such as craniosynostosis, facial fractures, and cleft palate.^495^ The use of a microscope enables minimally invasive incision, clear visualization of the complicated and delicate surgical site, as well as precise operation. Here, we discuss several applications of the surgical microscope in craniofacial surgeries.

##### Craniosynostosis

The use of a surgical microscope in a minimally invasive approach to craniosynostosis provides excellent visualization, illumination, and control of the surgical field, which potentially results in shorter operative time, hospitalization, and less blood loss.^496^ Teichgraeber et al.^93^ tested a microscopic approach and an open approach on 67 infants with craniosynostosis. A surgical microscope was used on 40 infants to perform appropriate synostectomy after placing incisions over the premature suture in the microscopic approach, while a coronal incision with cranial vault reconstruction was used in the open approach. Although the surgical outcomes of both approaches were equal, the microscopic approach resulted in less operative time (108 versus 210 min), less blood loss (75 versus 220 mL), and shorter hospital stay (2 versus 4 days). Later, Teichgraeber et al.^497^ reported his long-term comparison of the microscopic minimally invasive approach and the open approach for nonsyndromic craniosynostosis on 180 consecutive patients. The microscopic approach had equal surgical outcomes with the open approach and remained to be the treatment method for sagittal lambdoidal craniosynostosis, while for unicoronal or metopic patients it showed less favor.

##### Congenital defect

Kumar et al.^5^ reported their surgical treatment method of preauricular sinuses using a surgical microscope and evaluated the effect of the microscope on surgical outcomes. After incisions were used to expose the surgical site, magnification of the surgical microscope was employed for visualization during the blood removal and the meticulous ramification dissection. The magnification was useful for precise dissection to avoid any epithelial breach, which may lead to recurrence in preauricular sinus excision. Park and Diaz^498^ described a surgical approach to repair pediatric orbital floor fractures. The surgical microscope was brought into the field midway because of the suboptimal depth of vision and the inability to use two hands provided by the endoscope. The major role of the microscope was to provide excellent visualization of the entire orbital floor defect and to facilitate placement of a polyethylene implant with great ease. The authors also anticipated the advantages of the microscopic technique such as enhanced cosmesis and better exposure of orbital fracture without increased global retraction. Cleft palate repair and rerepair, which involve a deep and small surgical site, often require the use of a surgical microscope to aid visualization.^499^ Sommerlad^494^^,^^500^ performed palate repair and rerepair surgeries with radical dissection and retropositioning using a surgical microscope. The microscope allowed surgeons to perform a more precise dissection of the velar musculature and radical retropositioning of the levator palati muscle. In addition, the high magnification, which enabled visualization of the deep nasal mucosa and small vessels, facilitated the dissection of muscles off the nasal mucosa while preserving the submucosal vessels. Kato et al.^117^ performed 18 cleft lip operations and palatoplasty using either loupes or microscopy. The results show that microscope is more helpful than loupes in delicate operations such as cleft surgery because of the clearly observable surgical field, ensured precise procedures, comfortable positions, as well as the convenient share of view with the team provided by the microscope, without significantly increased preoperative preparation time or operating time. Furthermore, Mehendale et al.^501^ used the microscope for direct evaluation of the velar muscles in the surgery of velopharyngeal incompetence. The anatomy of velar muscles, which was assessed with the microscope, was used to make a final decision whether radical dissection and retropositioning or Hynes pharyngoplasty would be performed.

#### 4.5.2 Microsurgery

Surgical microscope has a great impact on reconstructive microsurgery. Its applications include hand surgery,^502^ head and neck reconstruction using free tissue transfer,^14^ nerve repair,^492^ anastomosis and duct reconstruction,^503^^,^^504^ lymphatic reconstruction,^86^ etc. The microscope can not only aid visualization and precise operation but also reduce surgical complications.

##### Hand microsurgery

High magnification provided by microscope such as 10× or more is necessary for hand surgery to see the tissue structures. It was said that the microsurgical techniques with the use of a microscope could yield a markedly improved success rate than loupes in the anastomoses of vessels smaller than 3.5 mm.^502^ It also has an important role for flexor tendon repair,^505^ nerve repair,^492^ finger replantation, and toe transplantation.

##### Nerve repair

Surgical microscope can offer high magnification for surgeons to see the nerve ends and to perform neurorrhaphy with suture for peripheral nerve repair. In a study of digital nerve repair,^506^ 45 patients were operated on using a microscope with a magnification of 14×. Normal two-point discrimination was achieved in 9 cases, 6 to 10 mm discrimination in 22 cases, and 11 to 15 mm discrimination with protective sensation in 18 cases. A review of six studies about cadaveric digital nerve repair^492^ concluded that the use of the microscope produces superior quality repair than loupes (60% versus 29%). In addition, the superiority of a microscope lies in the easier access to fascicles when digital nerves that are not expediently repaired lead to scarring and neuroma formation. Furthermore, the surgical microscope is helpful for the treatment of nerve palsy. Slooff^507^ performed intraplexal and/or extraplexal neurotizations with the aid of a surgical microscope on the brachial plexus, the weakness of which can lead to difficulty in the motion of arm.^508^ Geissler et al.^509^ operated on anterior interosseous nerve palsy, which was a complication of closed bone forearm fracture. A surgical microscope was used to open the epineurium longitudinally, to remove the bone spike perforating the median filter.

##### Finger replantation and toe transplantation

Surgical microscope can aid visualization of vessels and nerves during replantation and transplantation. Tamai et al.^510^ performed replantation of a completely amputated little finger in a 20-month-old child. The surgical microscope was used to observe the amputated part and to identify the volar digital artery, after which a fine blunt needle was inserted to the cut end of the artery for repair. Chen in Shanghai performed finger reattachment with both loupes (351 cases) and a surgical microscope (106 cases). The success rate of using loupe was 51% while that of using the microscope was 91.5%.^502^ Ohmori and Harii^511^ transplanted a second toe to an index finger using microvascular anastomosis. The microscope was used for anastomoses of the vessels and nerves, as well as the approximation of nerve with sutures after revascularization. The operation was successful in both function and cosmetic ways.

##### Head and neck reconstruction

Reconstruction operation of the head and neck area is usually required due to oncologic resection of head and neck cancer. Surgical microscope has been widely used during reconstruction to facilitate visualization and ensure the patency of microvascular anastomoses. Ross et al.^14^ did a 7-year retrospective review of 151 consecutive microvascular-free tissue transfer in head and neck reconstruction where they compared the use of a surgical microscope (84 cases) and loupes (67 cases). The vessels involved in reconstruction included arteries (external carotid artery, superior thyroid artery, the high branch of the external carotid artery) and veins (internal jugular vein, facial vein, external jugular vein, temporal vein), and the reconstructed sites included oral cavity, mandible, tongue, pharynx, maxilla or orbita, temporal bone, and parotid. Although the surgical microscope did not yield a significantly better outcome than loupes, it did result in a 97.6% success rate and a shorter hospitalization time.

##### Lymphatic reconstruction

Yamamoto et al.^86^ performed 40 cases of LVA on 12 patients with lower extremity lymphedema using a surgical microscope (OME-9000, Olympus) equipped with infrared illumination for intraoperative microscopic ICG lymphography guidance. The ICG guidance was used in 24 LVAs, where lymphatic vessels were enhanced in 22 cases. In addition, ICG guidance significantly shortened the time for detection and dissection of lymphatic vessels. In another study of 11 microsurgical free flaps, microscope-integrated ICG angiography was performed to visualize the flow over microanastomoses and to assess the transit time of blood flow between arterial and venous anastomosis.^267^ It was reported that the ICG imaging and its integration into the surgical microscope allow an “angiographic patency test” and provide a new level of safety in flap design.

##### Liver transplantation

Hepatic artery complication and biliary complication are two of the major concerns during living donor liver transplantation (LDLT). The high magnification provided by the surgical microscope is helpful for the reconstruction of vessels and small ducts, which can reduce complications during liver transplantation.

##### Hepatic artery reconstruction

Surgical microscope was found helpful with arterial anastomosis and to decrease hepatic artery thrombosis (HAT) during liver transplantation. In a 20-year study of liver transplants reported by Bade et al.,^512^ 252 transplants were performed without microsurgery, and in 23 cases microscope was used for microsurgical hepatic artery reconstruction. The group using the microscope had a lower incidence of HAT, a lower retransplantation rate, and increased 1-year survival. Guarrera et al.^94^ used a surgical microscope with a magnification of 12× to 16× for hepatic artery anastomosis in 14 segmental liver transplantations of children with no cases of HAT happened. Lee et al.^504^ reviewed their 325 LDLT using a surgical microscope (Leica M520 F40) with a magnification of 10× to 15× from 2008 to 2015. The microscope assistance enabled risk-free hepatic artery anastomoses, which yielded 84.8% 1-year survival, 75.2% 5-year survival, and only one episode of HAT. They indicated the advantage of using the surgical microscope beyond performing actual anastomosis as to survey for risk factors that can cause the HAT. Besides, the microscope assistance surpasses surgical loupe because it can avoid iatrogenic tearing of the vessel wall or compression of the vessel lumen. Seda-Neto et al.^513^ used Zeiss OPMI PENTERO 900 and OPMI Vario S88 surgical microscopes in the majority of 656 cases of pediatric liver transplantation for microvascular anastomosis. Each operation of arterial anastomosis took 20 to 30 min under 10× magnification. In addition, Zuo et al.^514^ reported 83 end-to-end hepatic artery anastomoses in 77 LDLT using an operating microscope, achieving a 1-year survival rate of 94% and a 5-year survival rate of 90%, with only two cases of HAT.

##### Biliary reconstruction

Surgical microscope used in biliary reconstruction can potentially reduce the incidence of biliary complications associated with LDLT. Yan et al.^503^ performed 32 LDLTs, where a surgical microscope was used for nine cholangiojejunostomies in small hepatic ducts of four donors. The diameter of the graft hepatic duct was smaller than 2 mm in seven cases and 2 to 3 mm in two instances. Magnification of 10× provided by the microscope was continuously used in these nine biliary reconstructions. The application of microsurgical technique with the microscope ensured the stability of the small operative field under high magnification and smooth handling of the ducts. Although the operating time using the microscope was almost twice that using loupes, it was concluded that the surgical microscope can help reduce the incidence of biliary complications of LDLT.

## 5 Discussion and Future Directions

Although first invented for otolaryngology, the surgical microscopes started to thrive after being introduced into neurosurgery. Compared with the early models, modern surgical microscopes have developed tremendously, with a large range of magnification options, stable and bright illuminations, well-designed optics and longer working distances, stabilized stands with good maneuverability and ergonomics, high-resolution visualization options, and especially the integration with various imaging modalities and AR technology that can facilitate image-guided surgery. The robotic feature added to the newest model is a new milestone in the development of surgical microscopes. Meanwhile, all-digital visualization offers additional freedom of movement and unlocks many new potential technologies. Nowadays, the surgical microscope has become a fundamental tool in many fields, including neuro and spine surgery, otolaryngology, ophthalmology, endodontics, and plastic and reconstruction surgery. It provides surgeons with clear, bright, and stabilized visualization of the surgical field and reveals more details of the delicate structures with its high magnification and integrated imaging modalities. Microsurgery with the operating microscope allows small incision, less muscle dissection, and less trauma. Decreased length of hospital stay and better cosmetic outcomes improve patients’ satisfaction after surgery.

Despite many advantages, there still exist some challenges for surgical microscopes. The fact of being cumbersome is the main limitation of the surgical microscope. Due to the large volume and heavy weight, the transportation of the surgical microscopes between ORs and the position of the microscope in the OR without interrupting surgery are of great concern. In addition, some studies indicated that setting up the microscope and repositioning during the surgery might prolong the duration of procedures.^25^^,^^73^ Fortunately, designs of modern surgical microscopes have improved the maneuverability to a great degree. Optics carrier and binocular tubes are capable of a wide range of tilt and rotation, enabling surgeons to view with convenient and comfortable postures. The integrated intraoperative imaging modules eliminated the interruption of surgical workflow since there is no need to remove the surgical microscope to capture images. Autofocusing is developed and added to the microscope so the surgeons can operate with any position and stay in focus easier and faster than before. With the high-precision motorized mechanics, moving the microscope becomes weightless; with the new robotic positioning features, surgeons can visualize the anatomy from different angles and no longer need to spend lots of time looking for the right position. Furthermore, a lightweight head-mount microscope, namely the Varioscope, is an option when mobility and maneuverability are required.^25^ It has switchable magnifications, autofocusing, and foot panel control.^515^ The adaptation with AR by Birkfellner^219^ empowered it for surgical guidance. It is a less complicated instrument so it has a shorter set-up time. Its good mobility enables the view of some structures that are hard to access with a common microscope.^73^ However, because of the absence of supporting structure, it has less stability than the common surgical microscope, thus it needs refocusing more often.

Another challenge of the surgical microscope is the skin burn caused by high-power illumination. To provide a bright visualization, especially when there are multiple viewers or cameras, the light source has to emit strong light, which may burn patients’ tissue. Skin burn can happen in various surgeries including ENT surgery,^148^^,^^516^ hand surgery,^517^ and plastic surgery,^518^ but ENT was the majority of all cases. Most cases happened in procedures that last for 3 h or more. Ophthalmic surgical microscope has also been reported to cause phototoxicity of the ocular surface and tear film,^519^^,^^520^ which results in decreased functionality of ocular cells. Light management has been developed in new models regarding spot size and working distance so the intensity of light that comes out of the light source changes with the magnification and the working distance of the surgical microscope. However, there are still several cases^516^^,^^518^^,^^521^ of skin burn being reported even using the new microscope models. Therefore, light management techniques need to be improved. The new robotic visualization system only uses one camera to capture the whole surgical field, and all observers can visualize the field through monitors. In this system, the reflected light no longer needs to be split to separate light paths for different observers. Hence, the intensity of illumination can be greatly reduced. Nevertheless, it is still worth exploring the appropriate level of illumination through clinical studies, which will serve as guidance for microscope users.

The high cost is also a big challenge for surgical microscopes to be commonly used. The price of a surgical microscope can be hundreds of thousands of dollars, depending on different configurations and features. The utilization of surgical microscopes in procedures is associated with added cost.^73^ Therefore, it is used in hospitals more than clinics considering financial capability. Nevertheless, the surgical microscope is a lifelong investment. It can benefit many procedures with various attractive features, meanwhile aid resident training and documentation. The enabled microsurgical techniques, improved surgical outcomes, and decreased hospital stay make it an invaluable tool.

Other challenges of using a surgical microscope include the potential intraoperative contamination,^25^^,^^522^ the steep learning curve,^73^^,^^459^ and the required extra skill and training.^11^ Some studies say that the surgical microscope is a potential source of infection in spinal surgery,^25^ while others say the opposite.^523^ It may depend on different surgical techniques, operations, and draping options. It is also very important for the surgical microscope to have a safe and convenient draping design. As for the learning curve and skill training, situations might change with the increased adoption of surgical microscope. The microscope has been reported to offer great assistance for resident training, enabled by its lateral observation ports, HD display, and high-resolution documentation. When it is adopted in more applications with its value being affirmed, hopefully it will provide more chances for education and get increased exposure to training.

For the future development of the surgical microscope, one important direction is to improve its modularity and adaptability.^130^ The requirement for the microscope changes with the users’ needs and the evolution of surgical techniques. The microscope should be ready for more sophisticated features to be added. The adaptation of fluorescence imaging filters and iOCT systems to surgical microscopes, which evolved from preliminary studies to commercially available modules, have proved their value in various intraoperative applications. The benefits of optical imaging lie in that they are minimally invasive and nonionizing.^360^ The next step is to utilize the adaptability of the microscope and investigate more advanced technologies, especially more optical imaging modalities, for surgical guidance.

Photoacoustic imaging, HSI, and LSCI are three promising imaging modalities that have been evaluated with surgical microscopes in a few studies. The depth-resolved photoacoustic image can not only provide subsurface information of the tissue as OCT but also reveal different structures that might be less visible in OCT images. However, its current adaptation with the microscope reduces the working distance. HSI acquires the spatial and spectral information of the tissue simultaneously; it not only aids intraoperative detection of tissue disorders and cancer but also provides surgical guidance such as dynamic oxygen saturation measurement and narrow-band visualization. LSCI, as a simple and low-cost method, can obtain the 2D perfusion map of the whole field and provide detailed spatiotemporal dynamics of blood flow changes in real-time, hence it can serve as an intraoperative blood flow monitoring tool. HSI and LSCI are two imaging modalities that are particularly advantageous to be integrated into the surgical flow through the surgical microscope. Although fluorescence imaging is the current most popular intraoperative imaging modality for tumor delineation and vasculature visualization, it requires a certain administration time of the injected dye, and it is liable to false positive findings due to incomplete clearness of the dye at end of the surgery. Besides, the interpretation of the brightness of fluorescence can also affect the diagnosis. HSI has been vastly evaluated with fluorescence imaging to improve its detection sensitivity and accuracy. However, it is worth noting that HSI alone is capable of serving as an intraoperative tool for various applications including tumor detection and microvascular visualization. Compared with fluorescence imaging, both HSI and LSCI are noncontact and label-free, not requiring any injection of contrast or dye. Hence, they can be used on-demand at any time during the surgery. Despite different principles, both technologies have a very simple system and relatively fast imaging speed. With well-developed image processing methods, they can provide a quantitative and dynamic assessment of the full field in real-time. Therefore, the integration of HSI and LSCI with the surgical microscope adds very little complexity to the system, takes minimal effort for physicians to adopt, and provides more information.

Polarization imaging is another imaging technique that is anticipated to be evaluated with the surgical microscope. Its implementation is simple and low-cost by applying two polarizers in front of the light source and detecting array. Polarization imaging can be used together with white light, fluorescence imaging, or even OCT to improve visualization and tissue differentiation during the surgery. For example, polarized light imaging was able to provide greater reliability for intraoperative nerve identification than normal visual inspection,^524^ dye-enhanced fluorescence polarization imaging could aid intraoperative delineation of cancer,^525^^–^^527^ and polarization-sensitive OCT as an extension function of OCT could yield higher contrast between different tissue types as well as aid the diagnosis of cancer.^526^^,^^528^ In addition to polarization imaging, the use of the endoscopic probe in the state-of-art robotic visualization system has revealed opportunities for integration of some imaging modalities that are not as easily adapted with conventional surgical microscopes, such as two-photon excited fluorescence, confocal microscopy, and Raman spectroscopy. The combination of the abovementioned imaging methods with surgical microscopes can reveal more information about biomarkers and anatomical structures than what can be seen from a merely magnified white-light image with minimal interruption of the surgical workflow. Furthermore, structured light was evaluated with a fluorescence-guided surgical microscope by Rodriguez for 3D shape reconstruction of the brain.^529^ The obtained 3D surface profile was used for light intensity correction of the autofluorescence signals, but with future development, this method is also potential to add depth perception in AR and aid accurate localization of anatomical structures.

Another expected future improvement of surgical microscopes is the capability of displaying the procedures. The high-end optics, high-resolution recording, and HD display have opened up tremendous abilities to share the information with trainees and colleagues, either during surgery or at a conference. It not only benefits education but also helps with the discussion for technique improvement. The 3D display and stereo headset even added depth perception to the shared view. Nowadays, the internet plays an important role in information sharing, and it opens up the possibility of live streaming of the procedures.^76^ Combining the documentation ability of surgical microscopes with live streaming and other advanced communication technologies, procedures become viewable in real-time through smartphones, computers, headsets, and large screens in a lecture room. A large team will be able to observe the operations and offer more assistance and feedback for the surgery. This might also be a great promotion of remote surgery, which allows the expertise of specialized surgeons to be available to patients worldwide. Although robotic surgery also enables the surgeon to operate remotely through a robot system, the expense to purchase and run the robot system is a huge drawback of robot-assisted remote surgery. Current AR-assisted platforms have made remote surgery much more cost-effective, but only with laptops or tablets, it is hard to conduct microsurgeries that require high magnifications. The integration of the surgical microscope with advanced communication technologies and AR-assisted platforms will definitely push the limit of remote surgery and bring a lot of change to the operating room.

Finally, surgical microscopes are expected to be adopted in more applications. Despite the fact that the surgical microscope is valued in various surgery types including neurosurgery, ENT, ophthalmology, dentistry, and plastic surgery, it might be more popular in some subspecialties and less embraced in others. For example, the neurosurgical microscope is commonly used by neurosurgeons in intradural, intramedullary, and extramedullary surgeries, but is less employed by orthopedic spine surgeons.^25^ The magnification of microscopes has brought a revolution in endodontics, while other fields in dentistry have not used it as much. Possible reasons include lack of exposure in training, low rates of utilization in general procedures, and less emphasis on microsurgical techniques. With the intrinsic capability of providing good visualization and the added imaging modules, the role of surgical microscopes in more clinical applications is waiting to be revealed. For instance, DSMs can be further evaluated for oral examination and periodontal surgery. The use of ophthalmic surgical microscopes can be more studied in cataract and glaucoma procedures. If integrated with HSI, surgical microscopes have the potential to aid the observation of seizures and larynx disorders.^355^^,^^530^ In some ENT surgeries, surgeons prefer an endoscope because it offers visualization of structures that are less visible under a microscope. Since integrated with iOCT, surgical microscopes have gained a better capability to reveal minute structures in the ear and larynx, which may increase the popularity of surgical microscopes in ENT surgery in the future. In addition, the endoscopic tool in the new robotic visualization system is a solution to this concern—surgeons are able to visualize the full field and the deep structure simultaneously in a picture-in-picture view. This is opening opportunities for surgical microscopes to be employed in more ENT surgeries.

In conclusion, the surgical microscope is a powerful tool that can offer optional magnifications, bright illumination, and clear visualization. It has been used in different types of surgeries and has improved surgical outcomes as well as surgeons’ ergonomics. It is anticipated that the integration of surgical microscopes with state-of-the-art optical imaging technologies will change the clinical practice in the operating room and benefit patients.
